# Impact of ambient gases on the mechanism of [Cs_8_Nb_6_O_19_]-promoted nerve-agent decomposition†
†Dedicated to the memory of Prof. Keiji Morokuma.
‡
‡Electronic supplementary information (ESI) available: (1) The calculated transition states, intermediates and products of the GB hydrolysis and their important geometry parameters (in Å) for X = SO_2_, (2) the calculated adsorption energies (in kcal mol^–1^) of NO_2_ radicals to Cs_8_Nb_6_O_19_, (3) Cartesian coordinates for all reported structures in xyz format. (structure.xyz). See DOI: 10.1039/c7sc04997h


**DOI:** 10.1039/c7sc04997h

**Published:** 2018-01-08

**Authors:** Alexey L. Kaledin, Darren M. Driscoll, Diego Troya, Daniel L. Collins-Wildman, Craig L. Hill, John R. Morris, Djamaladdin G. Musaev

**Affiliations:** a C. L. Emerson Center for Scientific Computation and Department of Chemistry , Emory University , Atlanta , Georgia 30322 , USA . Email: dmusaev@emory.edu; b Department of Chemistry , Virginia Tech , Blacksburg , Virginia , 24061 , USA . Email: jmorris@vt.edu; c Department of Chemistry , Emory University , Atlanta , Georgia 30322 , USA . Email: chill@emory.edu

## Abstract

The impact of ambient gas molecules (X), NO_2_, CO_2_ and SO_2_ on the structure, stability and decontamination activity of Cs_8_Nb_6_O_19_ polyoxometalate was studied computationally and experimentally. It was found that Cs_8_Nb_6_O_19_ absorbs these molecules more strongly than it adsorbs water and Sarin (GB) and that these interactions hinder nerve agent decontamination. The impacts of diamagnetic CO_2_ and SO_2_ molecules on polyoxoniobate Cs_8_Nb_6_O_19_ were fundamentally different from that of NO_2_ radical. At ambient temperatures, weak coordination of the first NO_2_ radical to Cs_8_Nb_6_O_19_ conferred partial radical character on the polyoxoniobate and promoted stronger coordination of the second NO_2_ adsorbent to form a stable diamagnetic Cs_8_Nb_6_O_19_/(NO_2_)_2_ species. Moreover, at low temperatures, NO_2_ radicals formed stable dinitrogen tetraoxide (N_2_O_4_) that weakly interacted with Cs_8_Nb_6_O_19_. It was found that both in the absence and presence of ambient gas molecules, GB decontamination by the Cs_8_Nb_6_O_19_ species proceeds *via* general base hydrolysis involving: (a) the adsorption of water and the nerve agent on Cs_8_Nb_6_O_19_/(X), (b) concerted hydrolysis of a water molecule on a basic oxygen atom of the polyoxoniobate and nucleophilic addition of the nascent OH group to the phosphorus center of Sarin, and (c) rapid reorganization of the formed pentacoordinated-phosphorus intermediate, followed by dissociation of either HF or isopropanol and formation of POM-bound isopropyl methyl phosphonic acid (i-MPA) or methyl phosphonofluoridic acid (MPFA), respectively. The presence of the ambient gas molecules increases the energy of the intermediate stationary points relative to the asymptote of the reactants and slightly increases the hydrolysis barrier. These changes closely correlate with the Cs_8_Nb_6_O_19_–X complexation energy. The most energetically stable intermediates of the GB hydrolysis and decontamination reaction were found to be Cs_8_Nb_6_O_19_/X-MPFA-(i-POH) and Cs_8_Nb_6_O_19_/X-(i-MPA)-HF both in the absence and presence of ambient gas molecules. The high stability of these intermediates is due to, in part, the strong hydrogen bonding between the adsorbates and the protonated [Cs_8_Nb_6_O_19_/X/H]^+^-core. Desorption of HF or/and (i-POH) and regeneration of the catalyst required deprotonation of the [Cs_8_Nb_6_O_19_/X/H]^+^-core and protonation of the phosphonic acids i-MPA and MPFA. This catalyst regeneration is shown to be a highly endothermic process, which is the rate-limiting step of the GB hydrolysis and decontamination reaction both in the absence and presence of ambient gas molecules.

## Abstract 2

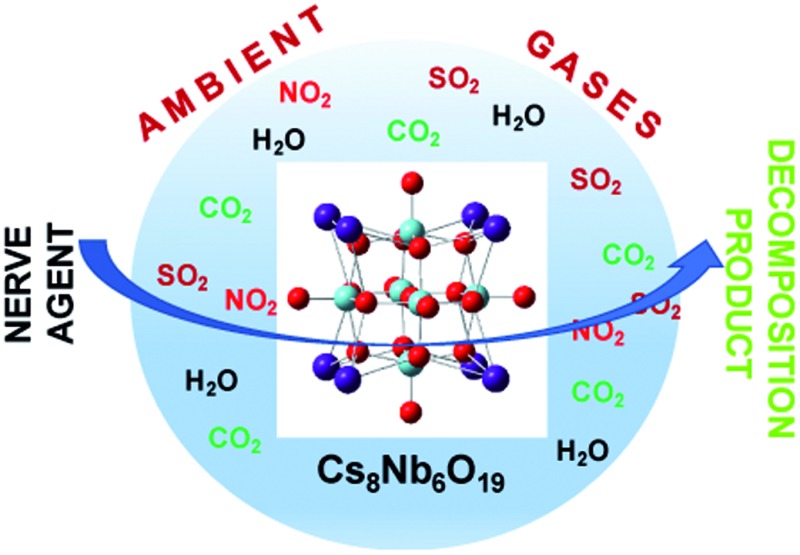
Polyoxoniobate catalyst, nerve agent decomposition, reaction mechanism, impact of ambient gases on the stability and reactivity of the polyoxoniobate.

## Introduction

The design of materials that can rapidly, fully, and catalytically decontaminate chemical warfare agents (CWAs) and other toxic compounds is an increasingly active area of research and one that presents some questions in fundamental chemistry.^1^–^5^ As suggested by enzymatic chemistry, some of the most effective strategies for CWA destruction involve catalyzed hydrolysis reactions.^4^ Specifically, it is well established that the P–X bonds (X = F, CN, SR, *etc.*) of organophosphorus (OP) nerve agents rapidly inactivate acetylcholinesterase (a serine hydrolase), the enzyme that facilitates hydrolysis of the neurotransmitter acetylcholine in the nervous system. This inactivation occurs through rapid nucleophilic addition and irreversible binding of the serine OH to the phosphorus atom of the nerve agent. Thus, an atomistic/molecular level understanding of the hydrolysis of OP compounds by nucleophilic addition and other processes may lead to the development of more effective materials and catalysts for nerve agent decontamination. Ongoing research efforts have identified several organic and inorganic materials, including metal–organic frameworks (MOFs, especially UiO-66, NU-1000 and MOF-808),^5^–^10^ polyoxometalates (POMs),^11^–^14^ MOF/POM hybrid materials,^15^,^16^ zirconium hydroxide,^17^ zeolites,^18^ and organic polymers,^19^ as effective OP hydrolysis materials.

Recently, the use of POMs to catalyze nerve agent decontamination has attracted wide attention, in part because POMs are molecular representations of metal oxides and are thus far more amenable than the latter to extensive synthetic compositional alteration and characterization at the molecular level.^20^,^21^ Polyoxoniobates (PONbs), including [Nb_6_O_19_]^8–^, are effective OP nerve agent hydrolysis compounds because their high negative charge densities (negative charge per polyanion oxygen) render them highly basic and nucleophilic. Thus, it is not surprising that the synthesis and in-depth analysis of the structures and reactivities of various (alkali and organic) salts of PONbs continue to be the focus of extensive studies.^22^,^23^ These studies show that the structures and, consequently, the catalytic activities of these materials for nerve agent decontamination depend on many factors, including (but not limited to) the nature of the counter-cation, the pH of the solution, the aggregate state (powder or solid-state material) of the catalyst, the real-time environmental conditions, and the nature and concentration of ambient gas molecules.

**Scheme 1:**
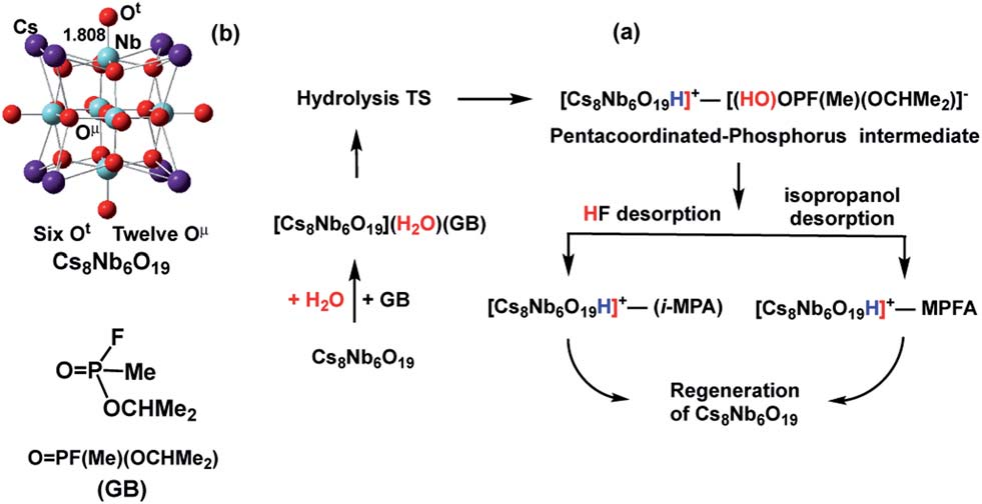
Schematics of: (a) the mechanism of Sarin (GB) hydrolysis (reported in ref. 24) promoted by the Cs_8_Nb_6_O_19_ polyoxoniobate and (b) the Cs_8_Nb_6_O_19_ polyoxoniobate.

Earlier research on Lindqvist hexaniobate alkali salts (M_8_Nb_6_O_19_, M = Li, K, Cs) reported rapid hydrolysis of the OP agent Sarin (GB, propan-2-yl methylphosphonofluoridate, see Scheme 1) both in aqueous solution and at the gas–surface interface.^11^ Small-angle X-ray scattering (SAXS) measurements showed aggregation of the OP compounds on the polyoxoniobate (PONb), which led to the suggestion that the reaction follows a general base hydrolysis mechanism.^12^ Our subsequent computational study on the mechanism of decomposition of GB by a Cs-salt of PONb, Cs_8_Nb_6_O_19_ (or CsPONb), confirmed the general base hydrolysis mechanism of this reaction at the gas–surface interface.^24^

Briefly, we have found that GB degradation by Cs_8_Nb_6_O_19_ includes the following elementary steps (see Scheme 1): (a) the adsorption of water and the nerve agent on the Cs_8_Nb_6_O_19_ species, (b) concerted dissociation of the adsorbed water molecule on a basic oxygen atom of the polyoxoniobate and nucleophilic addition of the nascent OH group to the phosphorus center of the nerve agent, (c) rapid reorganization of the resulting pentacoordinated phosphorus intermediate by dissociation of either HF or isopropanol, and formation of POM-bound isopropyl methyl phosphonic acid (i-MPA) or methyl phosphonofluoridic acid (MPFA), respectively. The calculations showed that the phosphonic acids i-MPA and MPFA are strongly bound to the protonated [Cs_8_Nb_6_O_19_H]^+^-core through hydrogen bonds and electrostatic interactions with the Cs counter-ions, suggesting that full catalyst regeneration may require additional treatment and depends on the nature of the counter-cations as well as the real-time (ambient) experimental conditions.

Although PONb catalysts have been shown to react with CWAs, the chemistry has yet to be characterized in the presence of ambient gases (for example, NO_2_, CO_2_ and SO_2_), which may affect the stabilities, structural motifs, and activities of the PONb catalysts. Because this issue is vital to the application of PONbs as decontamination catalysts in real conditions, this paper probes the impact of the common battlefield contaminants NO_2_, CO_2_ and SO_2_ on the structure, stability and decontamination activity of the exemplary PONb species Cs_8_Nb_6_O_19_. This study addresses in depth the effects of these ambient gases on the structures of the catalysts and the base hydrolysis mechanism for Sarin degradation using density functional theory (DFT) calculations and infrared (IR) spectroscopy.

## Results and discussion

### A. Structure of Cs_8_Nb_6_O_19_ in the presence of gaseous CO_2_, NO_2_ and SO_2_

Here, we divide our discussion into two subsections. First, we discuss the interaction of diamagnetic CO_2_ and SO_2_ molecules with Cs_8_Nb_6_O_19_; then, we present our findings on the interactions between NO_2_ radical and Cs_8_Nb_6_O_19_.

#### A1. Structure of Cs_8_Nb_6_O_19_ in the presence of gaseous CO_2_ and SO_2_

Our calculations reveal that Cs_8_Nb_6_O_19_ very strongly binds the ambient gas molecules CO_2_ and SO_2_ at various (terminal O^t^ and/or bridging O^μ^) sites to form Cs_8_Nb_6_O_19_/X adducts. As reported in Table 1, where we present the calculated adsorption energies, there is a clear preference of adsorption of both CO_2_ and SO_2_ at the terminal oxygen site rather than at the bridging oxygen site. Indeed, the calculated enthalpies and free energies of adsorption (presented as Δ*H*/Δ*G*) of Cs_8_Nb_6_O_19_ + X → Cs_8_Nb_6_O_19_/X upon coordination of X to the O^t^ and O^μ^ sites are –29.0/–23.2 and –16.7/–10.8 kcal mol^–1^ for X = CO_2_ and –47.6/–40.3 and –34.1/–26.6 kcal mol^–1^ for X = SO_2_, respectively.

**Table 1:** Adsorption energies (total electronic *E*, enthalpy *H* and Gibbs free energy *G* in kcal mol^–1^) defined as energy differences of the complex Cs_8_Nb_6_O_19_/X and its two separated fragments, Cs_8_Nb_6_O_19_ + X, where X = CO_2_, NO_2_ and SO_2_, as shown in the leftmost column. The superscripts “t” and “μ” indicate the position where the molecule is adsorbed on Cs_8_Nb_6_O_19_; Cs^N^ indicates NO_2_ adsorption to two Cs counter-cations in a symmetric manner

	Δ*E*	Δ*H*	Δ*G*
O^t^: Cs_8_Nb_6_O_19_ + CO_2_	–29.1	–29.0	–23.2
O^μ^: Cs_8_Nb_6_O_19_ + CO_2_	–16.8	–16.7	–10.8
O^t^: Cs_8_Nb_6_O_19_ + SO_2_	–48.2	–47.6	–40.3
O^μ^: Cs_8_Nb_6_O_19_ + SO_2_	–34.7	–34.1	–26.6
Cs^N^: Cs_8_Nb_6_O_19_ + NO_2_	–21.7	–22.2	–22.1
O^t^: Cs_8_Nb_6_O_19_ + NO_2_	–18.8	–18.7	–13.9
Cs^N^ and O^t^: Cs_8_Nb_6_O_19_ + 2NO_2_	–77.4	–74.6	–58.0
Cs_8_Nb_6_O_19_/CO_2_(O^t^) + H_2_O	–24.2	–22.4	–11.9
Cs_8_Nb_6_O_19_/SO_2_(O^t^) + H_2_O	–22.7	–21.0	–9.7
Cs_8_Nb_6_O_19_ + H_2_O	–24.6	–23.6	–17.3
Cs_8_Nb_6_O_19_/H_2_O + GB	–18.8	–17.4	–4.2
Cs_8_Nb_6_O_19_/H_2_O/CO_2_(O^t^) + GB	–18.0	–17.4	–2.4
Cs_8_Nb_6_O_19_/H_2_O/SO_2_(O^t^) + GB	–17.4	–16.4	–3.7

**Fig. 1:**
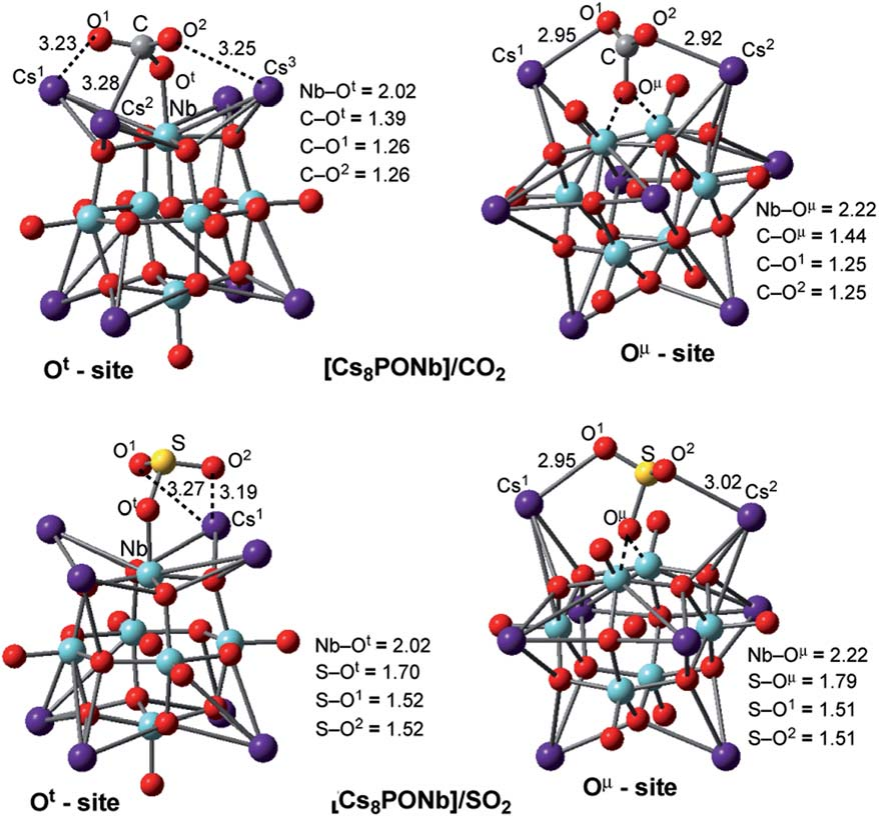
Adsorption of the ambient gas molecules CO_2_ and SO_2_ on Cs_8_Nb_6_O_19_ at the O^t^ sites (left) and bridging sites (right) with their important geometry parameters (in Å).

The factors that impact the strength of the Cs_8_Nb_6_O_19_/X bonding and site-preference of absorption were analyzed by investigating the resulting geometries of these complexes. From Fig. 1, where we present the most important geometries of the Cs_8_Nb_6_O_19_/X(O^t^) and Cs_8_Nb_6_O_19_/X(O^μ^) isomeric species for X = CO_2_ and SO_2_ (for full geometries of these species, see the ESI‡), we make the following conclusions:

(a) The CO_2_ molecule in Cs_8_Nb_6_O_19_/CO_2_(O^t^) is bound to Cs_8_Nb_6_O_19_*via* multiple interactions, including two (C=O)_CO_2__···Cs interactions and one C–O^t^ interaction. The long calculated bond distances for the Cs–O^1^, Cs–O^2^ and Cs–C interactions (3.23, 3.25 and 3.28 Å, respectively) indicate they are relatively weak. In contrast, the C–O^t^ interaction is stronger, with a bond distance of 1.39 Å. As a result of this strong interaction, the Nb–O^t^ distance is elongated from 1.80 Å to 2.02 Å (see Fig. 1). Thus, while the interactions with the Cs cations provide additional stability to the complex Cs_8_Nb_6_O_19_/CO_2_(O^t^), the primary interaction occurs between the C atom of CO_2_ and the terminal oxygen atom of Cs_8_Nb_6_O_19_.

In the Cs_8_Nb_6_O_19_/CO_2_(O^μ^) isomer, where CO_2_ interacts with a bridging oxygen, the C–O^μ^ bond distance is longer (calculated to be 1.44 Å) than the C–O^t^ bond distance in Cs_8_Nb_6_O_19_/CO_2_(O^t^), while the Cs–O^1^ and Cs–O^2^ interactions are slightly stronger (based on the calculated Cs–O^1^ and Cs–O^2^ distances). The above presented geometry parameters of Cs_8_Nb_6_O_19_/CO_2_(O^t^) and Cs_8_PONb/CO_2_(O^μ^) not only explain the calculated energy difference between these species, but are also consistent with the amounts of charge transfer from Cs_8_Nb_6_O_19_ to CO_2_: in the Cs_8_Nb_6_O_19_/CO_2_(O^t^) and Cs_8_Nb_6_O_19_/CO_2_(O^μ^) complexes, almost 0.8 |*e*| and 0.4 |*e*| negative charge is transferred from Cs_8_Nb_6_O_19_ to CO_2_, respectively. Furthermore, the putative CO_3_-group in Cs_8_Nb_6_O_19_/CO_2_(O^t^) has a total negative charge of 1.52 |*e*|.

**Fig. 2:**
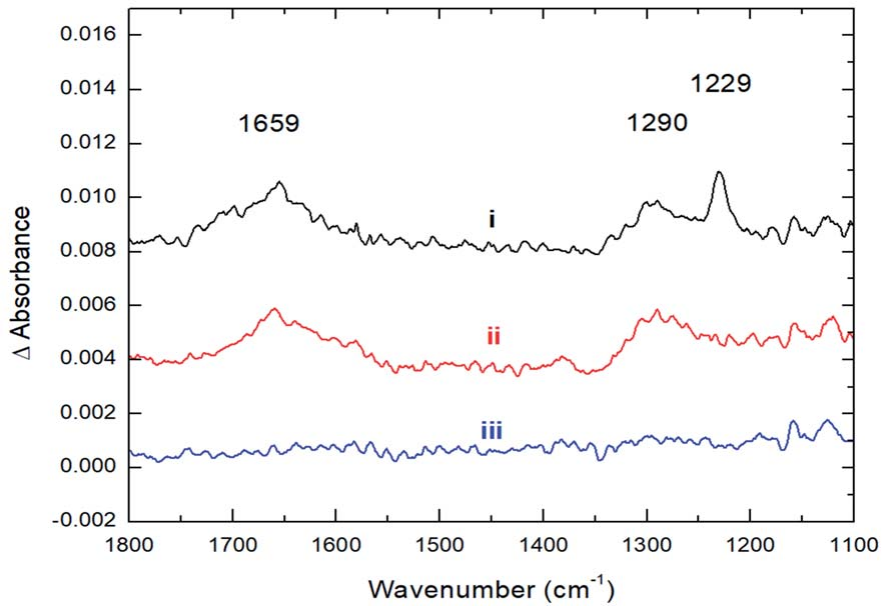
Infrared spectra recorded during and after the adsorption of CO_2_ onto Cs_8_Nb_6_O_19_ at 300 K. (i) Adsorption of 100 mTorr of CO_2_. (ii) After CO_2_ evacuation. (iii) After CO_2_ evacuation and thermal treatment at 423 K.

The strong coordination of CO_2_ to Cs_8_Nb_6_O_19_ is also supported by IR spectroscopic studies, where the adsorption of CO_2_ onto Cs_8_Nb_6_O_19_ was investigated by recording the infrared spectra before, during, and after exposure of Cs_8_Nb_6_O_19_ to a constant flux of CO_2_. Upon adsorption of CO_2_ on Cs_8_Nb_6_O_19_, three prominent features appeared in the infrared spectrum (Fig. 2, i) at 1659 cm^–1^, 1290 cm^–1^ and 1229 cm^–1^. Interestingly, we observed no feature in the infrared spectrum around 2300 cm^–1^, which would be associated with a linear CO_2_ molecule bound to the POM surface. The 1229 cm^–1^ and 1290 cm^–1^ bands are consistent with the IR-inactive symmetric stretch from the gas-phase (*ν*_1_(C–O)), which becomes IR active upon binding to the POM. The 1659 cm^–1^ band is likely related to the antisymmetric *ν*_3_(C–O) stretch of CO_2_/CO_3_ (occurs at 2349 cm^–1^ in the gas-phase^25^ and significantly redshifts upon adsorption). The presence of both the *ν*_1_ and *ν*_3_ bands in the infrared spectrum indicates a bent structure of the adsorbate at the surface. Upon evacuation of CO_2_ from the chamber, the 1229 cm^–1^ spectroscopic feature diminishes, indicating that this band corresponds to the vibrational motion of weakly bound CO_2_ species on the surface (Fig. 2, ii); however, the two other features (1659 and 1290 cm^–1^) persist until annealing the Cs_8_Nb_6_O_19_ sample at 423 K (Fig. 2, iii). The elevated temperature required to fully remove the CO_2_ suggests that the molecules responsible for these infrared bands are strongly bound to Cs_8_Nb_6_O_19_. The experimentally observed IR features are in full agreement with the DFT calculations (harmonic, un-scaled). Indeed, the two prominent IR active features at 1290 and 1659 cm^–1^, which are ascribed to the bending and asymmetric CO stretch motions of a bent CO_2_ molecule, are calculated to be 1316 and 1317 cm^–1^ (for the bend) and 1702 and 1719 cm^–1^ (for the asymmetric stretch) in Cs_8_Nb_6_O_19_/CO_2_(O^t^) and Cs_8_Nb_6_O_19_/CO_2_(O^μ^), respectively. Thus, the above presented experimental and computational analysis shows that a large fraction of the adsorbed CO_2_ strongly binds to Cs_8_Nb_6_O_19_. Once at the surface, the molecule adopts a bent geometry, which activates the *ν*_1_ vibrational motion toward absorption of infrared radiation.

(b) The geometric features of Cs_8_Nb_6_O_19_/SO_2_(O^t^) and Cs_8_Nb_6_O_19_/SO_2_(O^μ^) adducts are similar to those of their CO_2_ analogs: among the interactions, the strongest are the S–O^t^ and S–O^μ^ interactions, with 1.70 and 1.79 Å bond distances, respectively. These geometry parameters are consistent with the greater stability of the Cs_8_Nb_6_O_19_/SO_2_(O^t^) isomer. Furthermore, comparison of the calculated Cs_8_Nb_6_O_19_–X binding energies shows that SO_2_ interacts with Cs_8_Nb_6_O_19_ much more strongly, by nearly a factor of two, than CO_2_. However, both molecules clearly persist on the POM at ambient and well above ambient temperatures.

Further calculations showed that single Cs_8_Nb_6_O_19_ species can bind several CO_2_ and SO_2_ molecules. As seen in Table 2, where we have summarized the adsorption energies as a function of the number of molecules adsorbed at the six O^t^ sites, there is a pronounced monotonic convergence of the electronic and enthalpy binding energies up to *n* = 6. The free energies, on the other hand, reveal thermodynamic instability for CO_2_ adsorption at larger values of *n*, suggesting that only the Cs_8_Nb_6_O_19_/(CO_2_)_*n*_ species with *n* ≤ 4 are viable. The Cs_8_Nb_6_O_19_/(SO_2_)_*n*_ species may still be stable for *n* > 6.

**Table 2:** Adsorption of *n*(X) (X = CO_2_ and SO_2_ and *n* = 1 to 6) on Cs_8_Nb_6_O_19_ at its O^t^-sites, reported as incremental Δ*Z*_*n*,*n*–1_(Cs_8_Nb_6_O_19_/X) = *Z*_*n*_(Cs_8_Nb_6_O_19_/X) – Z_*n*–1_(Cs_8_Nb_6_O_19_/X) – *Z*(X) energies (where *Z* = *E*, *H* or *G*) (in kcal mol^–1^)

*n*	X = CO_2_			X = SO_2_		
	*Z* = *E*	*Z* = *H*	*Z* = *G*	*Z* = *E*	*Z* = *H*	*Z* = *G*
1	–29.1	–29.0	–23.2	–48.2	–47.6	–40.3
2	–22.9	–21.8	–11.2	–43.3	–42.4	–27.6
3	–19.6	–18.9	–9.6	–38.8	–37.1	–27.3
4	–13.3	–12.5	–0.7	–34.2	–32.9	–19.8
5	–7.3	–6.6	4.4	–26.9	–25.9	–14.9
6	–2.2	–2.0	6.9	–29.0	–27.9	–16.9

#### A2. Structure of Cs_8_Nb_6_O_19_ in the presence of NO_2_ radicals

As one might expect, the interaction of Cs_8_Nb_6_O_19_ with NO_2_ radicals is conceptually different than those discussed above for the diamagnetic CO_2_ and SO_2_ molecules. Indeed, at first, in the gas-phase and at low temperatures, NO_2_ radicals are in equilibrium with dinitrogen tetraoxide, N_2_O_4_, while higher temperatures shift the equilibrium towards nitrogen dioxide.^26^

**Fig. 3:**
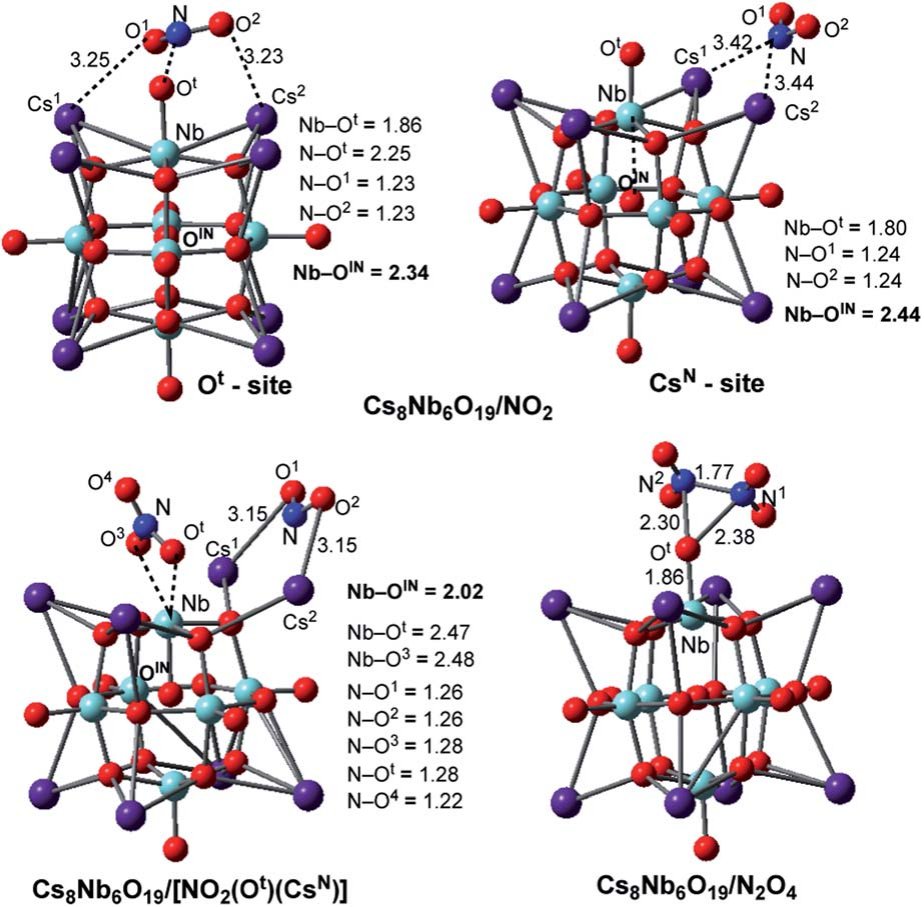
The calculated most energetically stable structures of NO_2_ and N_2_O_4_ adsorbed on Cs_8_Nb_6_O_19_, with their important geometry parameters (in Å).

The calculations show that N_2_O_4_ is planar, with an N–N bond distance of 1.85 Å, which is significantly longer than the average N–N single bond length of 1.45 Å; this species has a dimerization energy of Δ*E*/Δ*H*/Δ*G* = 19.8/17.3/5.6 kcal mol^–1^. Unlike NO_2_, N_2_O_4_ is diamagnetic and coordinates to the Cs_8_Nb_6_O_19_ catalyst (see Fig. 3) with a Cs_8_Nb_6_O_19_–N_2_O_4_ interaction energy of Δ*E*/Δ*H*/Δ*G* = –25.3/–25.3/–18.5 kcal mol^–1^. Secondly, as shown in Table 1 and Fig. 3, the “free” NO_2_ radical (at ambient temperature) can interact with polyoxoniobate to form the [Cs_8_Nb_6_O_19_]/NO_2_ adduct. This adduct exists in two energetically stable isomeric forms, Cs_8_Nb_6_O_19_/NO_2_(O^t^) and Cs_8_Nb_6_O_19_/NO_2_(Cs^N^); the most favorable form is Cs_8_Nb_6_O_19_/NO_2_(Cs^N^), where the N atom of NO_2_ interacts with two Cs centers (with Cs^1^–N and Cs^2^–N distances of 3.42 and 3.44 Å). To our surprise, these Cs–N interactions lead to electron transfer from Cs_8_Nb_6_O_19_ to NO_2_ in Cs_8_Nb_6_O_19_/NO_2_(Cs^N^), as evidenced by population analysis: the calculated Mulliken spin/charge is NO_2_(Cs^N^) = 0.34/–0.65 |*e*|. Thus, as a result of these interactions, almost 0.65 |*e*| charge is transferred from Cs_8_Nb_6_O_19_ to NO_2_; and, consequently, the Cs_8_Nb_6_O_19_ unit develops partial radical character. Further analysis shows that most of the unpaired spin of Cs_8_Nb_6_O_19_ is located on the internal O^IN^-center (∼0.24 |*e*|) and the bridging O^μ^ (∼0.14 |*e*|) located close to the Cs atoms coordinated to NO_2_, while the remaining spin is delocalized on all other atoms of the polyoxoniobate. Similarly, but to a lesser extent, NO_2_(O^t^) has a 0.67/–0.41 |*e*| spin/charge distribution in the Cs_8_Nb_6_O_19_/NO_2_(O^t^) isomer. The calculated Cs_8_Nb_6_O_19_–NO_2_ binding energies are Δ*H*/Δ*G* = –22.2/–22.1 and –18.7/–13.9 kcal mol^–1^ in Cs_8_Nb_6_O_19_/NO_2_(Cs^N^) and Cs_8_Nb_6_O_19_/NO_2_(O^t^), respectively.

Consequently, the acquired partial radical character of Cs_8_Nb_6_O_19_ in the Cs_8_Nb_6_O_19_/NO_2_(Cs^N^) complex significantly increases its NO_2_-affinity, enabling strong coordination of another (second) NO_2_ radical to the O^t^-site of polyoxoniobate and formation of the most thermodynamically favorable Cs_8_Nb_6_O_19_/[NO_2_(Cs^N^)NO_2_(O^t^)] singlet species. The calculated energy of the reaction Cs_8_Nb_6_O_19_/NO_2_(Cs^N^) + NO_2_ → Cs_8_Nb_6_O_19_/[NO_2_(Cs^N^)NO_2_(O^t^)] is Δ*E*/Δ*H*/Δ*G* = –55.8/–52.4/–35.9 kcal mol^–1^, while the energy of the Cs_8_Nb_6_O_19_/NO_2_(O^t^) + NO_2_ → Cs_8_Nb_6_O_19_/[NO_2_(Cs^N^)NO_2_(O^t^)] reaction is Δ*E*/Δ*H*/Δ*G* = –63.8/–61.5/–52.8 kcal mol^–1^ (see ESI‡ for more details). Thus, removal of NO_2_(O^t^) and/or NO_2_(Cs^N^) from Cs_8_Nb_6_O_19_/[NO_2_(Cs^N^)NO_2_(O^t^)] with two NO_2_-adsorbates requires greater energy than removing them from Cs_8_Nb_6_O_19_/NO_2_(O^t^) and/or Cs_8_Nb_6_O_19_/NO_2_(Cs^N^). The overall energy of the reaction Cs_8_Nb_6_O_19_ + 2NO_2_ → Cs_8_Nb_6_O_19_/[NO_2_(Cs^N^)NO_2_(O^t^)] is Δ*E*/Δ*H*/Δ*G* = –77.4/–74.6/–58.0 kcal mol^–1^ (see Table 1). To summarize, a relatively weak coordination of the first NO_2_ radical to Cs_8_Nb_6_O_19_ at the Cs sites promotes stronger coordination of the second NO_2_ molecule at the O^t^ site of Cs_8_Nb_6_O_19_. As a result, the coordination of the NO_2_ radicals to Cs_8_Nb_6_O_19_ is substantially stronger than that of CO_2_, yet slightly weaker than that of one SO_2_ molecule.

The reported highly stable Cs_8_Nb_6_O_19_/[NO_2_(Cs^N^)NO_2_(O^t^)] complex with two NO_2_ fragments can also be formed *via* N–N bond activation of the coordinated N_2_O_4_ molecule by the polyoxometalate catalyst. The former pathway (*i.e.* stepwise addition of two NO_2_ radicals to polyoxoniobate) may be valid at ambient temperature, while the latter process may occur at low temperature. In this paper, we did not study the N–N activation barrier; however, we found that the complex Cs_8_Nb_6_O_19_/[NO_2_(Cs^N^)NO_2_(O^t^)] (see Fig. 3) lies significantly lower in energy than the Cs_8_Nb_6_O_19_ + N_2_O_4_ dissociation limit, by Δ*E*/Δ*H*/Δ*G* = 57.6/57.3/52.4 kcal mol^–1^, and Δ*E*/Δ*H*/Δ*G* = 37.8/40.0/46.8 kcal mol^–1^ lower than the Cs_8_Nb_6_O_19_/N_2_O_4_ intermediate.

In order to better understand the factors impacting the strength of the Cs_8_Nb_6_O_19_/NO_2_ interaction, we also analyzed the geometry of the complex Cs_8_Nb_6_O_19_/[NO_2_(Cs^N^)NO_2_(O^t^)]. As seen in Fig. 3, where we present the most important geometries of the Cs_8_Nb_6_O_19_/NO_2_(O^t^), Cs_8_Nb_6_O_19_/NO_2_(Cs^N^) and Cs_8_Nb_6_O_19_/[NO_2_(Cs^N^)NO_2_(O^t^)] species (for full geometries of these species, see the ESI‡), in Cs_8_Nb_6_O_19_/[NO_2_(Cs^N^)NO_2_(O^t^)], the Nb–O^t^ bond is elongated to 2.47 Å; concurrently, the Nb–O^IN^ bonds (which are 2.34, 2.44 and 2.02 Å in these complexes, respectively) and N–O^t^ bonds (which are 1.28 and 2.25 Å in Cs_8_Nb_6_O_19_/[NO_2_(Cs^N^)NO_2_(O^t^)] and Cs_8_Nb_6_O_19_/NO_2_(O^t^), respectively) are formed. As a result, the complex Cs_8_Nb_6_O_19_/[NO_2_(Cs^N^)NO_2_(O^t^)] has one NO_3_^–*δ*^ fragment (*δ* = 0.4) and one NO_2_^–*δ*^ fragment (*δ* = 0.94) coordinated to the Cs-cations.

Above, we have shown that: (1) at ambient temperatures, a relatively weak coordination of the first NO_2_ radical to Cs_8_Nb_6_O_19_ confers partial radical character on the polyoxoniobate and promotes a stronger coordination of the second NO_2_ radical to form a stable diamagnetic Cs_8_Nb_6_O_19_/[NO_2_(Cs^N^)NO_2_(O^t^)] complex, and (2) at low temperatures, coordination of a weakly stable N_2_O_4_ molecule to Cs_8_Nb_6_O_19_ followed by facile N–N bond activation leads to the same Cs_8_Nb_6_O_19_/[NO_2_(Cs^N^)NO_2_(O^t^)] complex. Regardless of the formation mechanisms, in Cs_8_Nb_6_O_19_/[NO_2_(Cs^N^)NO_2_(O^t^)], the coordination of the NO_2_ radical positioned at O^t^ to Cs_8_Nb_6_O_19_ is substantially stronger than that of CO_2_, yet is slightly weaker than that of one SO_2_ molecule.

**Fig. 4:**
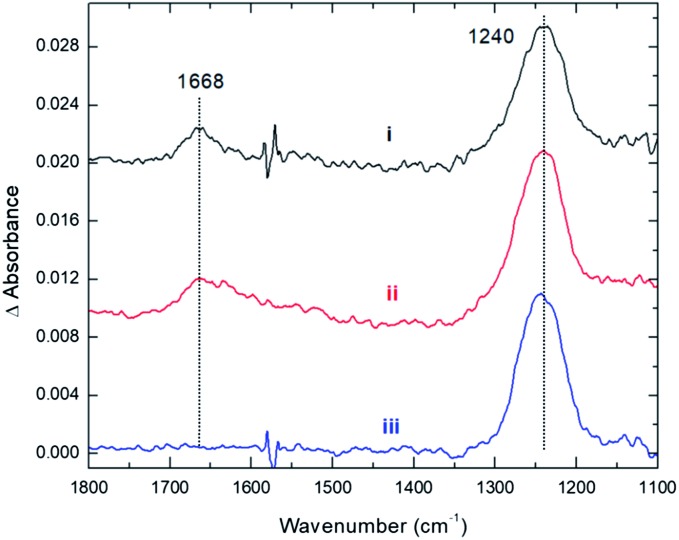
Infrared spectra recorded during and after the adsorption of NO_2_ onto Cs_8_Nb_6_O_19_ at 300 K. (i) Adsorption of 35 mTorr of NO_2_. (ii) After NO_2_ evacuation. (iii) After NO_2_ evacuation and thermal treatment at 423 K.

These conclusions from our computations are fully supported by experiments. As with CO_2_, the adsorption of NO_2_ onto Cs_8_Nb_6_O_19_ was probed with the use of infrared spectroscopy. Fig. 4 shows spectra for the interaction between NO_2_ and Cs_8_Nb_6_O_19_. The adsorption of NO_2_ on the surface resulted in two infrared vibrational features – one at 1668 cm^–1^ and another at 1240 cm^–1^ (Fig. 4, i). These features are attributed to adsorbate N–O stretches as opposed to POM motions, which appear below 1000 cm^–1^. Analysis of the calculated [Cs_8_Nb_6_O_19_]–NO_2_ complexes suggests that the 1668 cm^–1^ feature is likely due to an asymmetric NO stretch originating from the Cs_8_Nb_6_O_19_/N_2_O_4_ complex, which the calculations (unscaled) show to be at 1813 cm^–1^. For the other NO_2_ complexes, the highest frequency NO stretch is found below 1600 cm^–1^, *i.e.* at a lower frequency relative to the experimental peak. Taking into account the usual anharmonic correction, these modes will be found to be even further redshifted to lower frequencies. However, if we consider that the calculated asymmetric (strongly IR active) NO stretch of a free NO_2_ radical is 1752 cm^–1^, and the known experimental value^25^ is 1618 cm^–1^, the resulting frequency scale factor of 0.92 brings the asymmetric NO stretch of the Cs_8_Nb_6_O_19_/N_2_O_4_ complex to exactly 1668 cm^–1^. Moreover, if the measured 1668 and 1240 cm^–1^ peaks originate from the same thermal mixture of diamagnetic complexes, we expect that the 1240 cm^–1^ peak, which persists at higher temperatures (Fig. 4, iii), is due to the Cs_8_Nb_6_O_19_/[NO_2_(Cs^N^)NO_2_(O^t^)] complex, which is much more stable to thermal perturbation than Cs_8_Nb_6_O_19_/N_2_O_4_. The latter immediately transforms to Cs_8_Nb_6_O_19_/[NO_2_(Cs^N^)NO_2_(O^t^)] upon N–N activation by heating. Indeed, there is a group of IR-active NO stretching motions in the Cs_8_Nb_6_O_19_/[NO_2_(Cs^N^)NO_2_(O^t^)] complex in the 1346 to 1361 cm^–1^ (1238 to 1252 cm^–1^ scaled) region, which captures the measured 1240 cm^–1^ peak.

**Fig. 5:**
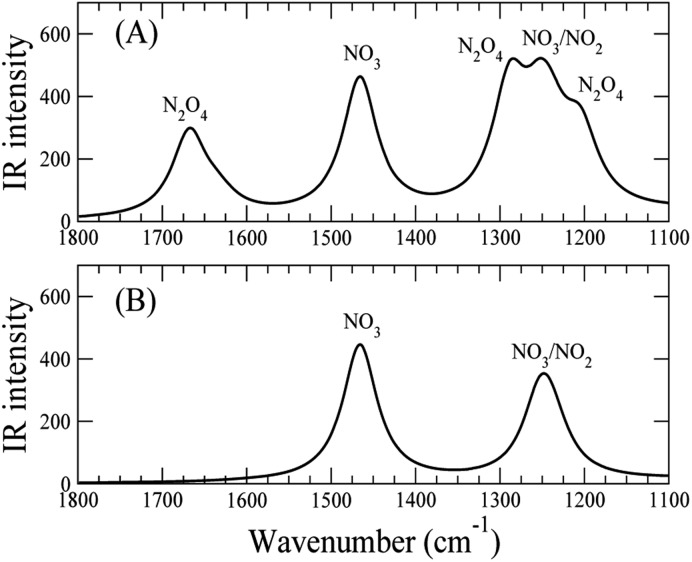
The calculated harmonic IR spectra (all in the NO stretch region), scaled by 0.92, of (A) the sum of the Cs_8_Nb_6_O_19_/N_2_O_4_ and Cs_8_Nb_6_O_19_/[NO_2_(Cs^N^)NO_2_(O^t^)] species; (B) the pure Cs_8_Nb_6_O_19_/[NO_2_(Cs^N^)NO_2_(O^t^)] species.

Thus, we present two spectra (shown in Fig. 5); one corresponds to low temperature, Fig. 5(A), which is the sum of Cs_8_Nb_6_O_19_/N_2_O_4_ and Cs_8_Nb_6_O_19_/[NO_2_(Cs^N^)NO_2_(O^t^)], and one corresponds to high temperature, Fig. 5(B), which is pure Cs_8_Nb_6_O_19_/[NO_2_(Cs^N^)NO_2_(O^t^)]. The frequency axis is scaled by the same factor of 0.92. The calculation is consistent with experiments with regard to the disappearance of the 1668 cm^–1^ peak and the persistence of the 1240 cm^–1^ peak. However, the peak at 1470 cm^–1^ (after scaling) in the calculated spectra, which is attributed to a local NO stretch of the NO_3_ unit in Cs_8_Nb_6_O_19_/[NO_2_(Cs^N^)NO_2_(O^t^)], is absent in the room-temperature experiments. This suggests that the formation of NO_3_ requires a significant amount of thermal energy.

Furthermore, experiments clearly show that NO_2_ is strongly bound to the POM, *i.e.* the 1240 cm^–1^ peak is present even after gas phase evacuation (Fig. 4, ii) and upon heating the POM to 423 K (Fig. 4, iii). In fact, thermal treatment up to 600 K was required to fully desorb the species, *i.e.* to remove NO_2_ radicals. This suggests that NO_2_ binds more strongly to the POM than CO_2_, which is consistent with the computational data for the Cs_8_Nb_6_O_19_/[NO_2_(Cs^N^)NO_2_(O^t^)] complex presented above (see Table 1 and the ESI‡).

Thus, the data presented above show that in the presence of ambient gas molecules of CO_2_, NO_2_ and SO_2_, Cs_8_Nb_6_O_19_ will absorb these molecules more strongly than the water and GB molecules required for hydrolysis of Sarin (see Table 1). This is expected to impact the hydrolysis of Sarin by Cs_8_Nb_6_O_19_ in the following two ways: first, because the ambient gas molecules coordinate to catalytically active O^t^-centers, they block these catalytically active centers, hindering water and Sarin coordination, and may alter the previously reported mechanism of Sarin hydrolysis by Cs_8_Nb_6_O_19_. Second, the interaction of an ambient gas molecule with Cs_8_Nb_6_O_19_ may change the electronic properties of the polyoxoniobate: this is expected to only impact the calculated energetics of the Sarin hydrolysis and to not significantly change the nature of the previously reported intermediates and transition state structures.

Below, we test the first hypothesis by (a) studying the full potential energy surfaces of GB hydrolysis by Cs_8_Nb_6_O_19_/X, where X = CO_2_ and SO_2_, and (b) comparing these new findings with our previous results on the same reaction in the absence of ambient gas molecules. For the sake of simplicity, we discuss in detail only the reaction mechanism (as well as the structures of the pre-reaction complexes, intermediates, transition states and products) for X = CO_2_ and compare these findings with those (previously reported) in the absence of ambient gas molecules. In addition, we briefly discuss, where appropriate, our findings for X = SO_2_ (full potential energy surfaces are available in the ESI‡). The reactivities of the NO_2_ and other radical species coordinated to Cs_8_Nb_6_O_19_ will be reported elsewhere.

### B. Hydrolysis of Sarin by Cs_8_Nb_6_O_19_/X species (where X = CO_2_ and SO_2_)

As we have shown previously^24^ and have briefly discussed above (see Scheme 1), Sarin hydrolysis by Cs_8_Nb_6_O_19_ is a multistep process, with coordination of a water molecule to the catalyst as the first step. The calculations (see Table 1) show that coordination of H_2_O to Cs_8_Nb_6_O_19_ to form Cs_8_Nb_6_O_19_/H_2_O is exothermic/exergonic by (presented as Δ*H*/Δ*G*) 23.6/17.3 kcal mol^–1^. In Cs_8_Nb_6_O_19_/H_2_O, the two hydrogens of the water molecule interact with one bridging (O^μ^) and one terminal (O^t^) oxygen atom of the polyoxometalate.

**Fig. 6:**
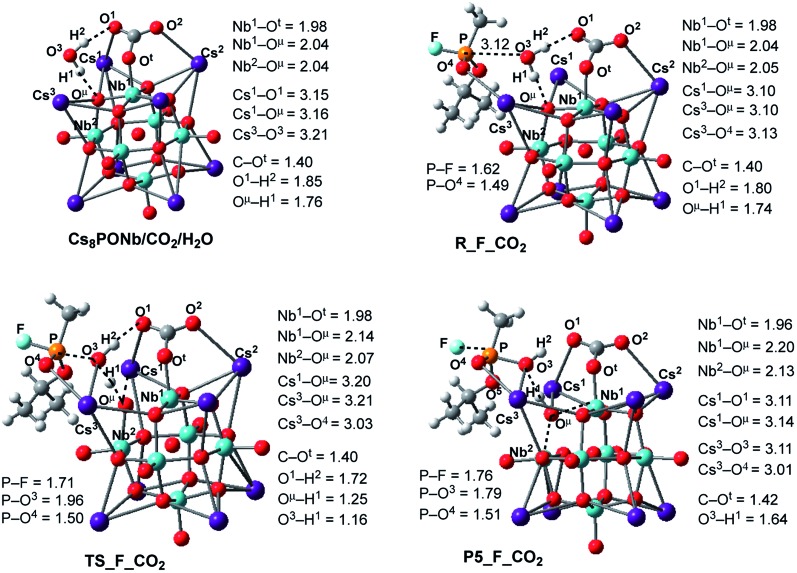
Calculated pre-reaction complexes, transition states and products of GB hydrolysis by Cs_8_Nb_6_O_19_/CO_2_ (*i.e.* the reaction Cs_8_Nb_6_O_19_/CO_2_ + H_2_O + GB → R-F_CO_2_ → TS-F_CO_2_ → P5-F_CO_2_) and their important geometry parameters (in Å). Similar structures for X = SO_2_ are presented in the ESI.‡

Water molecule coordination to the adduct formed when CO_2_ (or SO_2_) binds to the O^t^-center of Cs_8_Nb_6_O_19_ is a few kcal mol^–1^ less than that for “free” Cs_8_Nb_6_O_19_; the energies are –22.4/–11.9 and –21.0/–9.7 kcal mol^–1^ for X = CO_2_ and SO_2_, respectively. This effect is more pronounced for the Cs_8_Nb_6_O_19_/SO_2_ adduct than for Cs_8_Nb_6_O_19_/CO_2_. Furthermore, as seen in Fig. 6, the water coordination motif in Cs_8_Nb_6_O_19_/CO_2_/H_2_O is different from that in Cs_8_Nb_6_O_19_/H_2_O: because CO_2_ occupies the O^t^-position in Cs_8_Nb_6_O_19_/CO_2_, the H_2_O molecule is H-bonded to one bridging (O^μ^) and one CO (O^1^) oxygen atom (instead of O^t^). The calculated O^μ^–H^1^ and O^1^–H^2^ bond distances are 1.76 and 1.85 Å, respectively.

Following the formation of the Cs_8_Nb_6_O_19_/X/H_2_O complex, the addition of Sarin to this intermediate occurs. Previously, we examined several approaches of Sarin to Cs_8_Nb_6_O_19_ and found that nerve agent decomposition occurs when Sarin approaches the hydrated Cs_8_Nb_6_O_19_ segment with its O(sp^2^) and O(sp^3^) atoms (labeled in Fig. 6 and below as O^5^ and O^4^, respectively).^24^ The additional stabilization of the complex arises from the long-range O^4^···Cs^3^ ionic interaction. Thus, for the purposes of modeling the decomposition of Sarin in the presence of carbon and sulfur dioxide, it is sufficient to examine the most energetically favorable pathway, similar to that previously reported for the case with no ambient gas molecules. In keeping with the previously established shorthand notation, the pre-reaction complex of this reaction pathway is labeled as R–F_X.

The present calculations show that the coordination of Sarin (GB) to Cs_8_Nb_6_O_19_/X/H_2_O is exothermic by –18.0/–2.4 and –17.4/–3.8 kcal mol^–1^ for X = CO_2_ and SO_2_, respectively. Inspection of the structure of the Cs_8_Nb_6_O_19_/X/H_2_O/GB, R–F_X, intermediate reveals a non-covalently bonded [Cs_8_Nb_6_O_19_/CO_2_/H_2_O]–GB complex. For example, as seen in Fig. 6, in contrast to the coordination of GB to Cs_8_Nb_6_O_19_/H_2_O, in Cs_8_Nb_6_O_19_/CO_2_/H_2_O/GB, the nerve agent is coordinated to one of the Cs centers of the Cs_8_Nb_6_O_19_-core with its P=O^4^ double bond. Several H-bonds also exist between the GB ligand and the [Cs_8_Nb_6_O_19_/CO_2_/H_2_O]-fragment. In this complex, the calculated Cs^3^–O^4^ bond distance is 3.13 Å and the nascent P–O^3^(OH_2_) bond distance is 3.12 Å.

In the next step, hydrolysis of the coordinated water molecule occurs between the coordinated gas molecule X, the bridging oxygen (O^μ^) of the Cs_8_Nb_6_O_19_-core and the phosphorus center of Sarin. The transition state associated with this process, TS-F_CO_2_, is shown in Fig. 6 (for TS-F_SO_2_, see the ESI‡). As seen in this figure, at TS-F_CO_2_, the breaking O^3^–H^1^ bond of the water molecule is elongated to 1.16 Å, and the forming O^μ^–H^1^ bond distance becomes 1.25 Å. In addition, Nb^1^–O^μ^ and Nb^2^–O^μ^ bonds are slightly elongated and the Cs^3^–O^4^ bond is slightly shortened. Importantly, the Cs^3^-center of the Cs_8_Nb_6_O_19_ core also interacts with the oxygen (O^3^) of water and provides additional support for hydrolysis. Here, the other coordinates of interest are the P–O^3^(H_2_O) bond and the P–F bond.

**Fig. 7:**
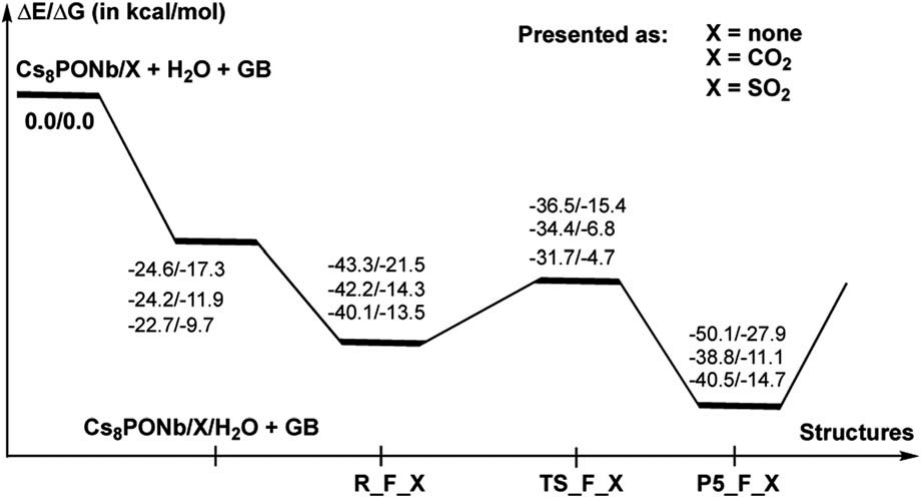
Potential energy profile of the hydrolysis of Sarin (GB) by Cs_8_Nb_6_O_19_ and Cs_8_Nb_6_O_19_/X(O^t^), where X = CO_2_ or SO_2_. Δ*E* and Δ*G* are the changes in the electronic and Gibbs free energies and are calculated relative to the reactants Cs_8_Nb_6_O_19_/X(O^t^) + H_2_O + GB.

As seen in Fig. 6, P–O^3^(H_2_O) undergoes a major reduction from 3.12 Å in R-F_CO_2_ to 1.96 Å in TS-F_CO_2_. Its P-F counterpart, located *trans* to the water-activated molecule, extends from the typical single bond in R-F_CO_2_, with an increase from 1.62 Å to 1.71 Å in TS-F_CO_2_. Similar geometry changes at the hydrolysis transition state were observed for X = SO_2_ (see the ESI‡). As seen in Fig. 7, the calculated Δ*E*/Δ*G* barrier heights relative to R-F_X are 7.8/7.5 and 8.4/8.8 kcal mol^–1^ for X = CO_2_ and SO_2_, respectively. These values are slightly larger than the values of 6.8/6.1 kcal mol^–1^ calculated for the reaction in the absence of these ambient gas molecules; this suggests that common battlefield contaminants may impair the hydrolytic decomposition of nerve agents under operational conditions.

The hydrolysis product is a pentacoordinated-phosphorus complex P5-F_X with a trigonal bipyramidal structure around the central phosphorus atom. In our previous paper, we showed that this intermediate exhibits multiple isomeric forms.^24^ Here, we discuss only the energetically most favorable form, which is directly connected to the transition state TS-F_X. For example, as seen in Fig. 6, the formed P–O^3^ bond in P5-F_CO_2_ contracts to 1.79 Å, while its P-F counterpart, located *trans* to the activated water molecule, extends to 1.76 Å. The broken O^3^–H^1^ bond is elongated to 1.64 Å and the Nb^1^–O^μ^ and Nb^2^–O^μ^ bonds are elongated to 2.20 and 2.13 Å, respectively. Concurrently, the Cs^3^–O^3^ bond of 3.11 Å is formed to provide additional stabilization to the pentacoordinated-phosphorus complex, similar to that previously reported in P5-F.^24^ Based on the calculated Mulliken charge distribution, the resulting P5-F_X complexes can be labeled as a [(GBOH^–^)–(Cs_8_Nb_6_O_19_H/X)^+^] ion-pair system.

As shown in Fig. 7, the hydrolysis of Sarin, *i.e.* the reaction Cs_8_Nb_6_O_19_/X + H_2_O + GB → R-F_X → TS-F_X → P5-F_X for X = none, CO_2_ or SO_2_, is exergonic by –50.1/–27.9, –38.8/–11.1 and –40.5/–14.7 kcal mol^–1^ (presented as Δ*H*/Δ*G*) and proceeds over energy barriers of 6.8/6.1, 7.5/7.5 and 8.4/8.8 kcal mol^–1^ (calculated relative to the pre-reaction intermediate R-F_X), respectively. The reaction R-F_X → TS-F_X → P5-F_X is exothermic for X = none and SO_2_ by 6.8/6.4 and 0.4/1.2 kcal mol^–1^, respectively, but is endothermic by 3.4/3.2 kcal mol^–1^ for X = CO_2_. These energy values allow us to conclude that the presence of ambient gas molecules increases the energies of the stationary points relative to the asymptote of the reactants. This is likely the result of a different charge distribution on Cs_8_Nb_6_O_19_/X relative to Cs_8_Nb_6_O_19_, where X acquires a net negative charge of 0.8 to 0.7 |*e*|, as we showed above. Furthermore, X coordinates to the O^t^ reactive center and disables its hydrolytic activity. The presence of ambient gases increases the hydrolysis barrier by 1.0 to 2.5 kcal mol^–1^, and this change in the energy barrier is closely associated with the Cs_8_Nb_6_O_19_–X complexation energy: the stronger the Cs_8_Nb_6_O_19_–X bond, the higher the barrier for Sarin hydrolysis.

### C. Pentacoordinated P5-F_X intermediate dissociation

Once the pentacoordinated P5-F_X species is formed, the reaction can proceed along several paths, as discussed in our previous paper.^24^ In order to evaluate the role of the catalyst in the course of the reaction, as above, here we discuss in detail only those pathways that directly involve the catalyst. These are the HF and isopropanol elimination and, ultimately, desorption pathways. The accompanying products of these paths are isopropyl methyl phosphonic acid (i-MPA) and methyl phosphonofluoridic acid (MPFA), respectively. It is evident that in order to form HF or isopropanol, protonation of the fluoride or oxygen centers of the isopropoxy ligand is required. Furthermore, in order to facilitate regeneration of the catalyst, the ultimate proton source should be the O^μ^H^1^-group of the [Cs_8_Nb_6_O_19_H/X]^+^ cation. However, these processes are expected to be very complex and may proceed *via* multiple mechanisms. One of these processes could involve any surrounding water molecules, which are expected to be present in real experimental conditions. In this mechanism, a water molecule located close to the fluoride or oxygen atoms of the iopropoxy ligand is expected to donate its proton to these groups (to form HF and/or isopropanol, respectively) and compensate by removing the proton from the O^μ^H^1^-group of the catalyst *via* a H-bonding network. This process depends on multiple factors (including, but not limited to, the concentration of water in the system and the reaction temperature) and was not studied in this paper.

**Fig. 8:**
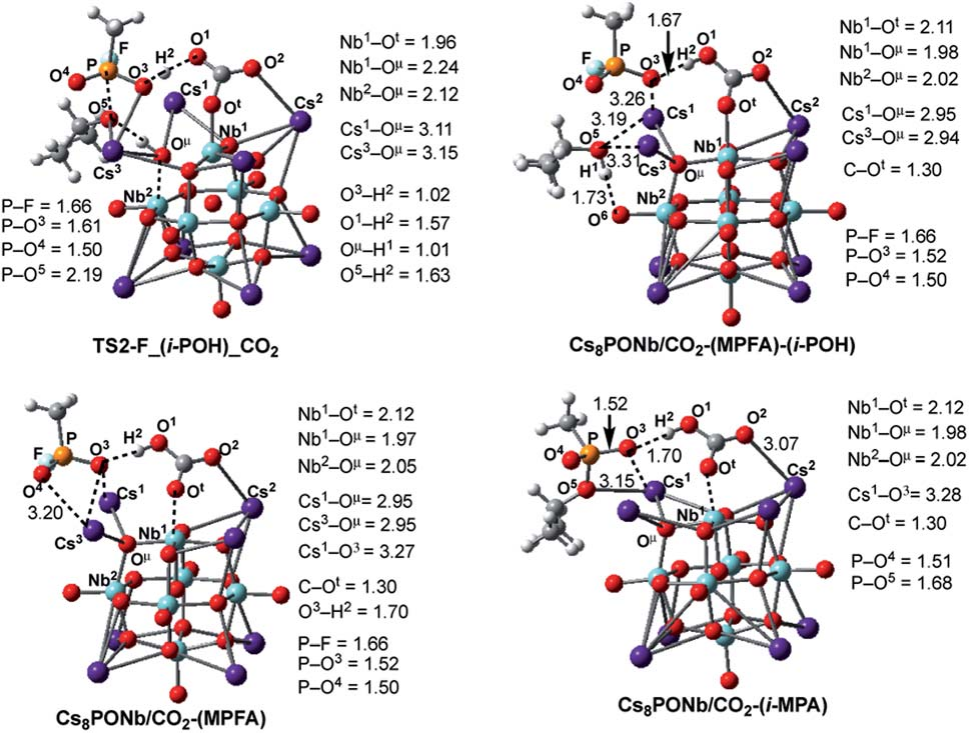
The calculated transition states, intermediates and products of the dissociation of pentacoordinated P5-F_CO_2_ (*i.e.* reactions: P5-F_CO_2_ → Cs_8_Nb_6_O_19_/CO_2_-(MPFA)-(i-POH) → Cs_8_Nb_6_O_19_/CO_2_ + MPFAH + (i-POH) and P5-F_X → Cs_8_Nb_6_O_19_/CO_2_-(MPA)-HF → Cs_8_Nb_6_O_19_/CO_2_ + MPAH + HF) with their important geometry parameters (in Å). Similar structures for X = SO_2_ are presented in the ESI.‡

**Fig. 9:**
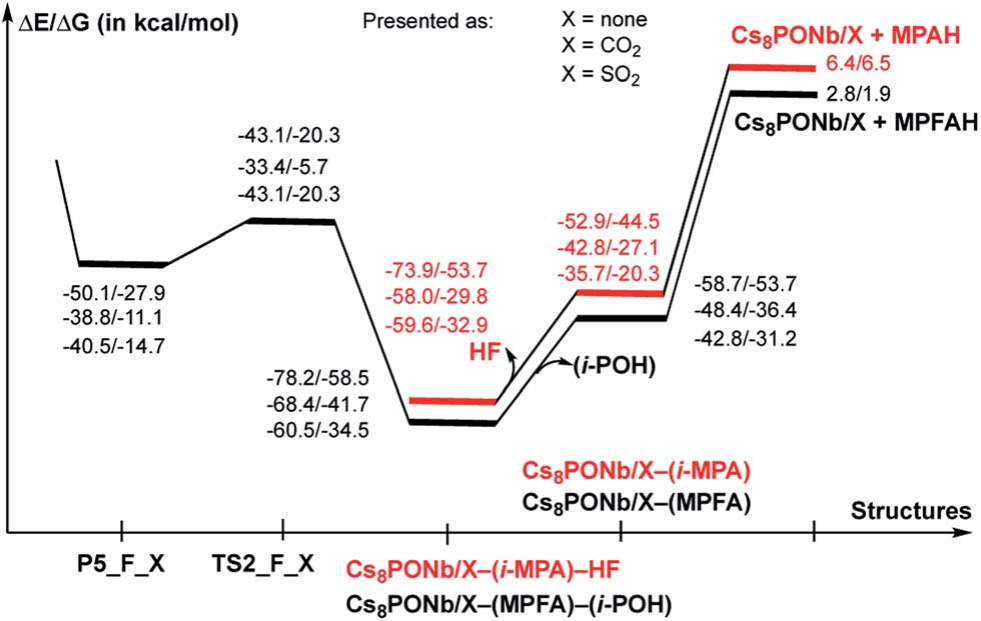
Potential energy profiles of the decomposition reaction of pentacoordinated-phosphorus intermediate P5-F_X, where X = CO_2_ and SO_2_ (*i.e.* reactions: P5-F_X → Cs_8_Nb_6_O_19_/X-(MPFA)-(i-POH) → Cs_8_Nb_6_O_19_/X + MPFAH + (i-POH) and P5-F_X → Cs_8_Nb_6_O_19_/X-(MPA)-HF → Cs_8_Nb_6_O_19_/X + MPAH + HF). Δ*E* and Δ*G* are the changes in the electronic and Gibbs free energies and are calculated relative to the reactants Cs_8_Nb_6_O_19_/X(O^t^) + H_2_O + GB.

Another possible mechanism of HF and/or isopropanol formation is direct removal of the proton from the O^μ^H^1^-group of the catalyst by the fluoride and/or isopropoxide ligands, respectively. As shown previously for the Cs_8_Nb_6_O_19_ catalyst (*i.e.*, in the absence of ambient gas molecules),^24^ these processes occur with very small energy barriers which have no contribution to the overall outcome of the decontamination reaction but lead to the most energetically stable intermediates, Cs_8_Nb_6_O_19_-(i-MPA)-HF and Cs_8_Nb_6_O_19_-(MPFA)-(i-POH), respectively (see Fig. 8). Here, we performed an extensive search to locate the transition states TS2-F_HF_X and TS2-F_(i-POH)_X that lead to either HF and i-MPA or isopropanol (i-POH) and MPFA from the most stable pentacoordinated intermediate P5-F_X (where X = CO_2_ or SO_2_). Ultimately, we were able to locate only the TS2-F_(i-POH)_X transition state (see Fig. 8). The search for the HF formation transition state TS2-F_HF_X was unsuccessful and always led to either the Cs_8_Nb_6_O_19_/X-(i-MPA)-HF intermediate, its derivative F^–^···H^+^···O^μ^ intermediate, or the pre-reaction complex P5-F_X. For example, in Fig. 8, we present the intermediates, transition states and products involved in pentacoordinated P5-F_CO_2_ intermediate dissociation alone, with their important geometry parameters (for those of X = SO_2_, see the ESI‡). The relative energies of these species, calculated from the P5-F_X pre-reaction complex, are given in Fig. 9.

As seen in Fig. 8, at the transition state TS2-F_(i-POH)_CO_2_ associated with the formation of i-POH and MPFA, the activated P–O^5^(Osp^2^) bond extends to 2.19 Å from 1.68 Å in P5-F_CO_2_, with simultaneous formation of a double H-bond network (O^5^–H^1^ = 1.63 Å and O^1^–H^2^ = 1.57 Å) as a precursor to the charge-preserving double proton transfer. In other words, as the proton of the O^μ^H^1^-group moves to Sarin, the proton on the PO^3^H^1^ group of Sarin migrates back to the Cs_8_Nb_6_O_19_/X-core to form [HXO^t^]^q^, where X = CO_2_. As a result, the formed P–O^3^(H_2_O) bond is shortened from 1.79 Å in P5-F_CO_2_ to 1.61 Å in the transition state.

As seen in Fig. 9, the calculated energy barriers from P5-F_X are 7.0/7.6, 5.4/5.4 and 3.6/6.5 kcal mol^–1^ for X = none, CO_2_ and SO_2_. Thus, the presence of these gas molecules in the reaction mixture slightly reduces the pentacoordinated P5-F_X intermediate dissociation barrier (by 2 to 4 kcal mol^–1^). Furthermore, the changes in the i-POH and MPFA formation energy barriers correlate with the Cs_8_Nb_6_O_19_–X complexation energy: the stronger the Cs_8_Nb_6_O_19_–X bond, the smaller the i-POH and MPFA formation energy barriers.

Comparison of the calculated energetics for the dissociation of the pentacoordinated intermediate with those for hydrolysis (*i.e.* formation of the pentacoordinated intermediate) show that, in general, the hydrolysis step is a rate-determining step for all reported species, and the presence of ambient gas molecules increases this energy barrier only slightly, by 2 to 4 kcal mol^–1^. Furthermore, the height of this rate-determining energy barrier correlates with the Cs_8_Nb_6_O_19_–X complexation energy: the stronger the Cs_8_Nb_6_O_19_–X bond, the more difficult the hydrolysis of GB by the Cs_8_Nb_6_O_19_ catalyst.

### D. Catalyst regeneration

Thus, the facile dissociation of the P5_F_X species, as reported previously for P5_F, yields HF and i-MPA and/or i-POH and MPFA products. All these species are initially bound to Cs_8_Nb_6_O_19_, as shown in Fig. 8 for X = CO_2_ and the ESI‡ for X = SO_2_. For example, in the Cs_8_Nb_6_O_19_/CO_2_-(i-MPA)-HF complex, the (i-MPA)-fragment forms a hydrogen bond with the O^μ^ and OH-unit of the HOCOO^t^ fragment, respectively. The HF molecule, on the other hand, in the presence of an X molecule, prefers to form an [F^–^···H^+^] ion-pair prior to dissociation. Similarly, in the Cs_8_Nb_6_O_19_/CO_2_-(MPFA)-(i-POH) complex, the (i-POH) molecule and MPFA-fragment are hydrogen bonded to another O^t^ atom and the OH-unit of the HOCOO^t^ fragment, respectively: the calculated H^1^–O^6^ and O^3^–H^2^ bond distances are 1.73 and 1.67 Å, respectively. In addition, there are electrostatic interactions between the O center of the isopropanol molecule and two Cs cations of the Cs_8_Nb_6_O_19_/CO_2_ core, with Cs^1^–O^5^ and Cs^3^–O^5^ bond distances of 3.19 Å and 3.31 Å, respectively. The formed P–O^3^ fragment also interacts with the Cs^1^-center, with a Cs^1^–O^3^ distance of 3.16 Å.

As seen in Fig. 9, the formed Cs_8_Nb_6_O_19_/X-MPFA-(i-POH) is the energetically lowest structure on the potential energy surface of the entire GB hydrolysis and decontamination reaction by Cs_8_Nb_6_O_19_ both in the absence and presence of ambient gas molecules. The intermediate Cs_8_Nb_6_O_19_/X-(i-MPA)-HF is found to be only slightly higher in energy. As we mentioned previously,^24^ the high stability of these intermediates is due in part to the strong hydrogen bonds between the adsorbates and the Cs_8_Nb_6_O_19_/X-core; however, it is also due to the additional stabilizing interactions between the Cs counter-ions and the electronegative atoms of the nerve-agent fragments.

Desorption of HF and isopropanol from Cs_8_Nb_6_O_19_/X-(i-MPA)-HF and Cs_8_Nb_6_O_19_/X-MPFA-(i-POH) requires 15.2/12.7 and 20.0/5.3 kcal mol^–1^ energy, respectively, for X = CO_2_. Dissociation of HF and isopropanol only slightly modifies the geometries of the products Cs_8_Nb_6_O_19_/X-(i-MPA) and Cs_8_Nb_6_O_19_/X-MPFA fragments (for example, see Fig. 8 for X = CO_2_) compared with the Cs_8_Nb_6_O_19_/X-(i-MPA)-HF and Cs_8_Nb_6_O_19_/X-MPFA-(i-POH) adducts; therefore, this will not be discussed in detail.

However, both i-MPA in Cs_8_Nb_6_O_19_/X-(i-MPA) and MPFA in Cs_8_Nb_6_O_19_/X-MPFA are strongly bound to the protonated [Cs_8_Nb_6_O_19_/X]H^+^-core. Thus, regeneration of the catalyst requires deprotonation of the [Cs_8_Nb_6_O_19_/X]H^+^-core and protonation of the phosphonic acids i-MPA and MPFA. This step of the reaction, which forms a final decontaminated form of the GB and re-generated catalyst, is found to be highly endothermic/endergonic, *i.e.* 6.4/6.5 and 2.8/1.9 kcal mol^–1^ for i-MPAH and MPFAH formation, respectively. Overall, the last steps of the reaction, *i.e.* the reactions Cs_8_Nb_6_O_19_/X-(i-MPA)-HF → Cs_8_Nb_6_O_19_/X + HF + (i-MPAH) and Cs_8_Nb_6_O_19_/X-MPFA-(i-POH) → Cs_8_Nb_6_O_19_/X + (i-POH) + MPFAH, are highly prohibitive and require energies of 80.3/60.2 (X = none), 64.4/36.3 (X = CO_2_), and 66.0/39.4 (X = SO_2_) kcal mol^–1^ and 81.0/60.4 (X = none), 71.2/43.6 (X = CO_2_), and 63.3/36.4 (X = SO_2_) kcal mol^–1^, respectively. Furthermore, deprotonation of the [Cs_8_Nb_6_O_19_/X]H^+^-core and protonation of the phosphonic acids i-MPA and MPFA is expected to be very complex and may proceed *via* several pathways depending on the reaction conditions. One of these may involve any surrounding water molecules, in real experimental conditions, *via* a concerted protonation-deprotonation mechanism involving the hydrogen-bonded water-based network. However, this process depends on multiple factors (including, but not limited to, the concentration of water in the system and the reaction temperature) and was not studied in this paper. We also failed to locate a transition state associated with the direct deprotonation of [Cs_8_Nb_6_O_19_/X]H^+^ and protonation of the phosphonic acids because of the high stability of the corresponding pre-reaction complexes Cs_8_Nb_6_O_19_/X-(i-MPA) and Cs_8_Nb_6_O_19_/X-MPFA. The solution to this issue requires special comprehensive experimental and computational studies, which are in progress.

## Conclusions

This paper, for the first time, addresses the impact of environmentally-significant ambient gas molecules, NO_2_, CO_2_ and SO_2_, on the structure, stability and decontamination activity of a basic polyoxometalate species. Specifically, Cs_8_Nb_6_O_19_ in the presence of these gases has been studied in depth by complementary computational and experimental approaches. It was found that:

(1) Cs_8_Nb_6_O_19_ absorbs ambient gas molecules of X = CO_2_, NO_2_ and SO_2_ more strongly than it absorbs water or Sarin (GB) molecules. The calculated Cs_8_Nb_6_O_19_–X binding energy follows the trend for Δ*G* (X = CO_2_) < Δ*G* (NO_2_) < Δ*G* (SO_2_).

(2) The impacts of the diamagnetic CO_2_ and SO_2_ molecules on polyoxoniobate Cs_8_Nb_6_O_19_ are fundamentally different than that of the NO_2_ radical. At ambient temperatures, weak coordination of the first NO_2_ radical to Cs_8_Nb_6_O_19_ confers partial radical character on the polyoxoniobate and promotes a stronger coordination of the second NO_2_ radical to form a stable diamagnetic Cs_8_Nb_6_O_19_/(NO_2_)_2_ species; meanwhile, at low temperatures, NO_2_ radicals form weakly stable dinitrogen tetraoxide (N_2_O_4_), which interacts weakly with Cs_8_Nb_6_O_19_.

(3) Similar to the case without ambient gas molecules, reported previously,^24^ in the presence of X, GB hydrolysis by Cs_8_Nb_6_O_19_/X proceeds *via* general base hydrolysis involving: (a) adsorption of water and the nerve agent on the Cs_8_Nb_6_O_19_/X catalyst, (b) concerted hydrolysis of the adsorbed water molecule on a basic oxygen atom of the polyoxoniobate and nucleophilic addition of the nascent OH group to the phosphorus center of the nerve agent, (c) rapid reorganization of the resulting pentacoordinated-phosphorus intermediate followed by dissociation of either HF or isopropanol with formation of POM-bound isopropyl methyl phosphonic acid (i-MPA) or methyl phosphonofluoridic acid (MPFA), respectively.

(4) Cs_8_Nb_6_O_19_ adsorbs ambient gas molecules X at its basic O^t^ (or O^μ^) reactive centers, which shields them from involvement in the base hydrolysis. As a result, one of the O centers of the coordinated ambient gas molecule becomes an active hydrolysis center. This increases the energies of the stationary points relative to the asymptote of the reactants and increases the hydrolysis barrier. These changes are closely correlated with the Cs_8_Nb_6_O_19_–X complexation energy; the stronger the Cs_8_Nb_6_O_19_–X bond, the higher the barrier for Sarin hydrolysis.

(5) The most energetically stable products of the GB hydrolysis and decontamination reaction are Cs_8_Nb_6_O_19_/X-MPFA-(i-POH) and Cs_8_Nb_6_O_19_/X-(i-MPA)-HF both in the absence and presence of ambient gas molecules. The high stability of these intermediates is due in part to the strong hydrogen bonds between the adsorbates and the protonated [Cs_8_Nb_6_O_19_/X/H]^+^-core and to interactions between the Cs counterions and the electronegative atoms of the adsorbates.

(6) Desorption of HF or/and (i-POH) and regeneration of the catalyst requires deprotonation of the [Cs_8_Nb_6_O_19_/X/H]^+^-core with protonation of the phosphonic acids i-MPA and MPFA. Regeneration of the catalyst is a highly endergonic process and is the rate-limiting step for GB hydrolytic decontamination, both in the absence and presence of ambient gas molecules.

## Notes

### A. Computational and experimental procedures

#### A1. Computational methodology

A major computational challenge in the present work is to properly describe the non-covalent interactions involving the various ions in the systems studied: the Cs^+^ counter-cations, [Nb_6_O_19_]^8–^ with its large negative charge, and the H^+^ and OH^–^ ions resulting from heterolytic water dissociation. These interactions are expected to be well described by the M06-L density functional,^27^ a pure density functional designed for transition metal bonding and non-covalent interactions. Therefore, all presented calculations have been carried out with the M06-L density functional, as implemented in the Gaussian09 code.^28^ In these calculations, we used the 6-31++G(d,p) basis set for the elements S, P, F, O, C, H and the Lanl2dz basis set with corresponding Hay–Wadt effective core potentials for Nb and Cs, as implemented in Gaussian09. The sets of diffuse functions (++) were added specifically to obtain proper descriptions of the diffuse charge densities and long-range interactions. All reported stationary points were confirmed to have either all real frequencies (minima) or one imaginary frequency (transition states). The latter were further verified to connect the corresponding minima by IRC calculations. All reported enthalpy and Gibbs free energies were computed at a temperature of 298.15 K and 1 atm pressure.

#### A2. Experimental methodology

The synthesis of Cs_8_Nb_6_O_19_·14H_2_O followed a known literature procedure. A solution of cesium hydroxide (14.6 g, 50% by weight) was heated to 90 °C in an Erlenmeyer flask. 5 g of hydrous, amorphous niobium oxide was added in small portions, with full dissolution of each portion before addition of the subsequent portion. Evaporative crystallization yielded giant hexagonal crystals.

Infrared spectroscopic experiments were performed in a stainless-steel high-vacuum chamber with a base pressure of ∼1 × 10^–8^ Torr. The Cs_8_Nb_6_O_19_ sample was pressed, as a 7 mm diameter disk, into a tungsten grid, which was then clamped onto a sample mount coupled to a precision manipulator. An empty region of the grid was used to monitor the gas phase species in the chamber and was also employed as a background for surface adsorption and desorption studies. The grid was resistively heated, and the temperature was monitored *via* a K-type thermocouple spot-welded adjacent to the sample. A PID controller mediated the sample temperature to within ±1 K. Details of the vacuum chamber and sample mount can be found in a previous publication.^29^ An FTIR spectrometer (Thermo, Nicolet, Nexus 470 FTIR) with an external liquid-N_2_-cooled MCT-A detector and a spectral resolution of 2 cm^–1^ was used for collection of the infrared spectra.

## Conflicts of interest

There are no conflicts to declare.

## Supplementary Material

Supplementary information

Supplementary information
