# Supplementary material for: Impact of ambient gases on the mechanism of [Cs8Nb6O19]-promoted nerve-agent decomposition
Source: Chem Sci. 2018 Jan 8;9(8):2147–58. doi: 10.1039/c7sc04997h (PMC5896467; doi:10.1039/c7sc04997h)
Supplement: Supplementary file 1 [file SC-009-C7SC04997H-s001.pdf]

Electronic Supplementary Material (ESI) for Chemical Science.
This journal is © The Royal Society of Chemistry 2018

Supporting Information

Impact of Ambient Gases on the Mechanism of the [$Cs_8Nb_6O_{19}$]-Promoted Nerve-Agent Decomposition

Alexey L. Kaledin,[a)] Darren M. Driscoll,[b)] Diego Troya,[b)] Daniel Collins-Wildman,[c)] Craig L. Hill,[*,c)] John R. Morris,[*,b)] and Djamaladdin G. Musaev,[*,a,b)]

a) Cherry L. Emerson Center for Scientific Computation, Emory University, 1515 Dickey Drive, Atlanta, Georgia 30322, USA

b) Department of Chemistry, Virginia Tech, Blacksburg, Virginia, 24061, USA

c) Department of Chemistry, Emory University, 1515 Dickey Drive, Atlanta, Georgia 30322, USA

* E-mail: dmusaev@emory.edu (DGM), jmorris@vt.edu (JRM) and chill@emory.edu (CLH)

Table of Contents

1. **Figure S1.** The calculated transition states, intermediates and products of the GB hydrolysis and their important geometry parameters (in Å) for X = SO_2,..S2

2. **Figure S2**. The calculated transition states, intermediates and products of the pentacoordinated P5-F_SO_2 dissociation (i.e. reactions: P5-F_SO_2 → $Cs_8Nb_6O_{19}$/SO_2-(MPFA)-(*i*-POH) → $Cs_8Nb_6O_{19}$/SO_2 + MPFAH + (*i*-POH) and P5-F_X → $Cs_8Nb_6O_{19}$/SO_2-(MPA)-HF → $Cs_8Nb_6O_{19}$/SO_2 + MPAH + HF) with their important geometry parameters (in Å)…………………………………………………… S3

3. **Figure S3.** The calculated adsorption energies (in kcal/mol) of NO_2 radicals to $Cs_8Nb_6O_{19}$..........S4

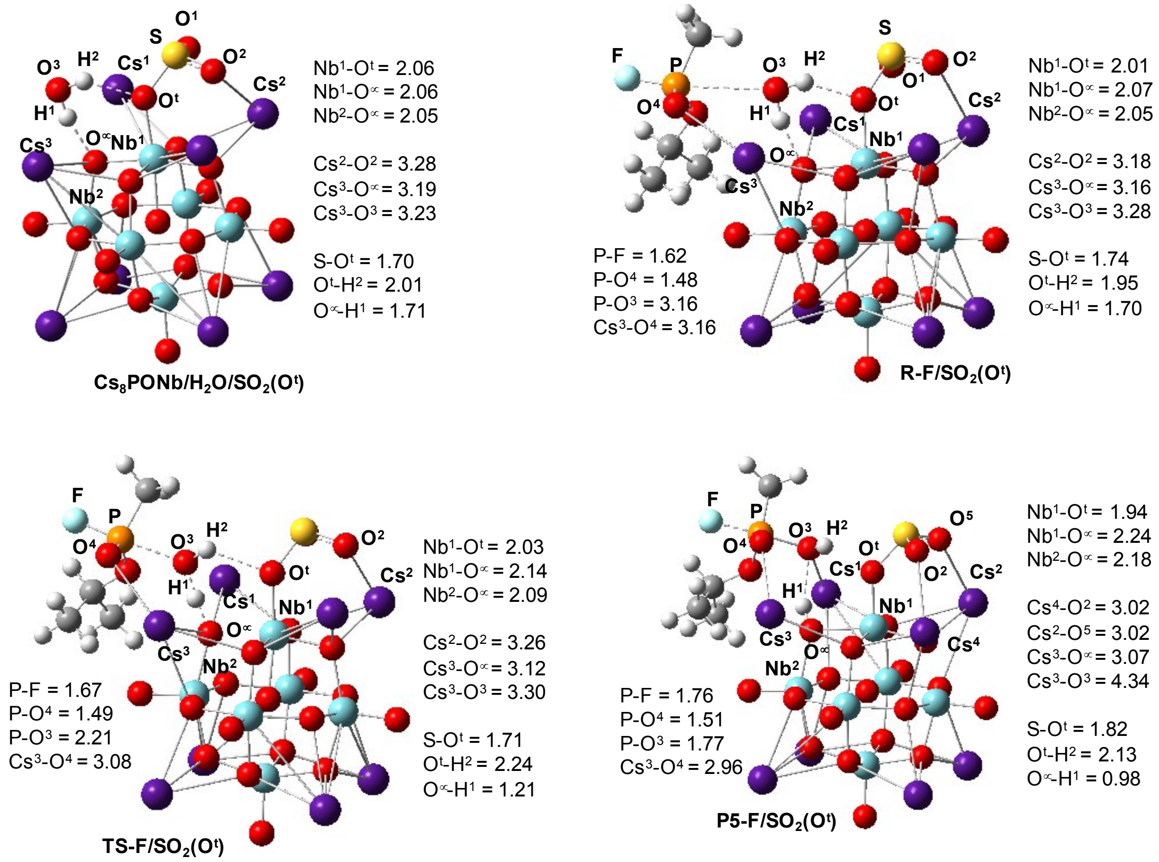

Figure S1. Calculated pre-reaction complexes, transition state and product of the GB hydrolysis by $Cs_8Nb_6O_{19}/SO_2$ (i.e. reaction $Cs_8Nb_6O_{19}/SO_2 + H_2O + GB \rightarrow$ R-F_$SO_2 \rightarrow$ TS-F_$SO_2 \rightarrow$ P5-F_SO_2**)** and their important geometry parameters (in Å).

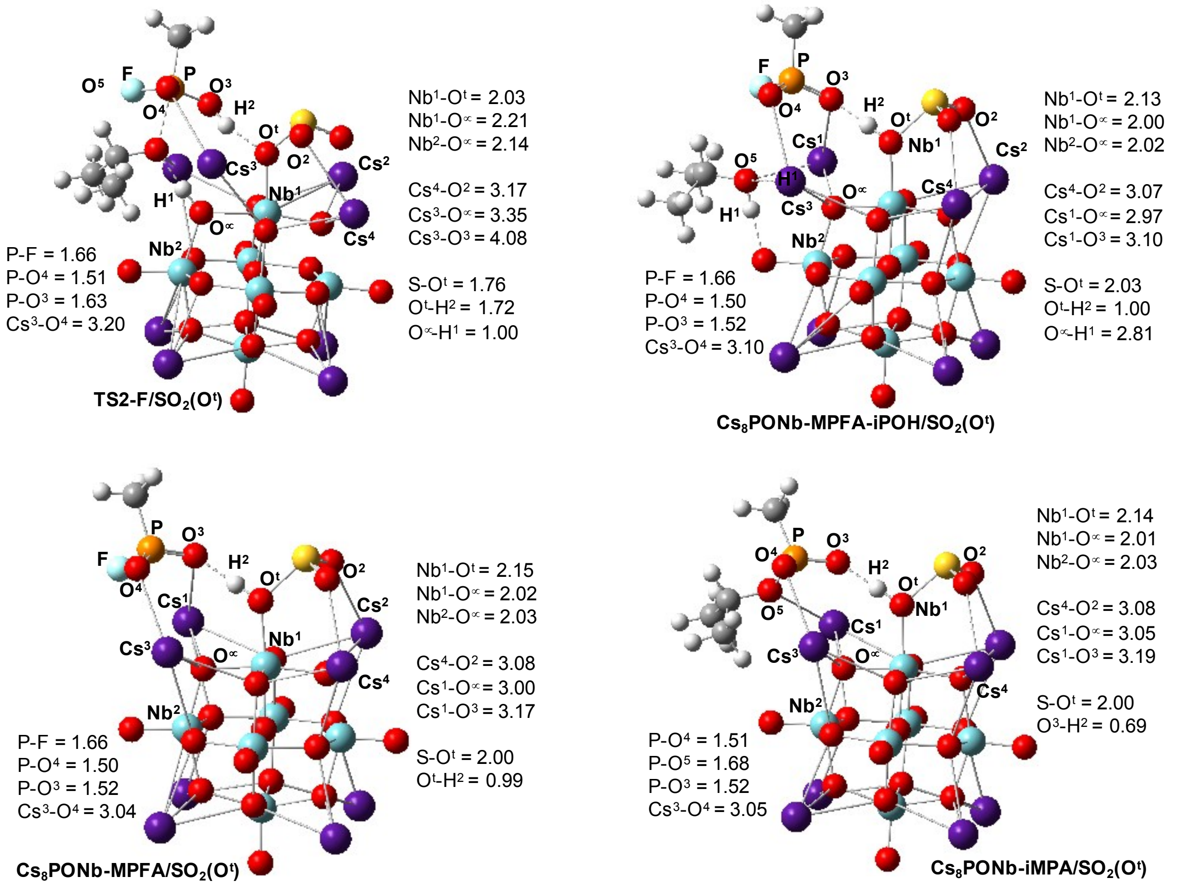

Figure S2. The calculated transition states, intermediates and products of the pentacoordinated P5-F_SO_2 dissociation (i.e. reactions: P5-F_SO_2 $\rightarrow$ $Cs_8Nb_6O_{19}/SO_2$-(MPFA)-(*i*-POH) $\rightarrow$ $Cs_8Nb_6O_{19}/SO_2$ + MPFAH + (*i*-POH) and P5-F_X $\rightarrow$ $Cs_8Nb_6O_{19}/SO_2$-(MPA)-HF $\rightarrow$ $Cs_8Nb_6O_{19}/SO_2$ + MPAH + HF) with their important geometry parameters (in Å).

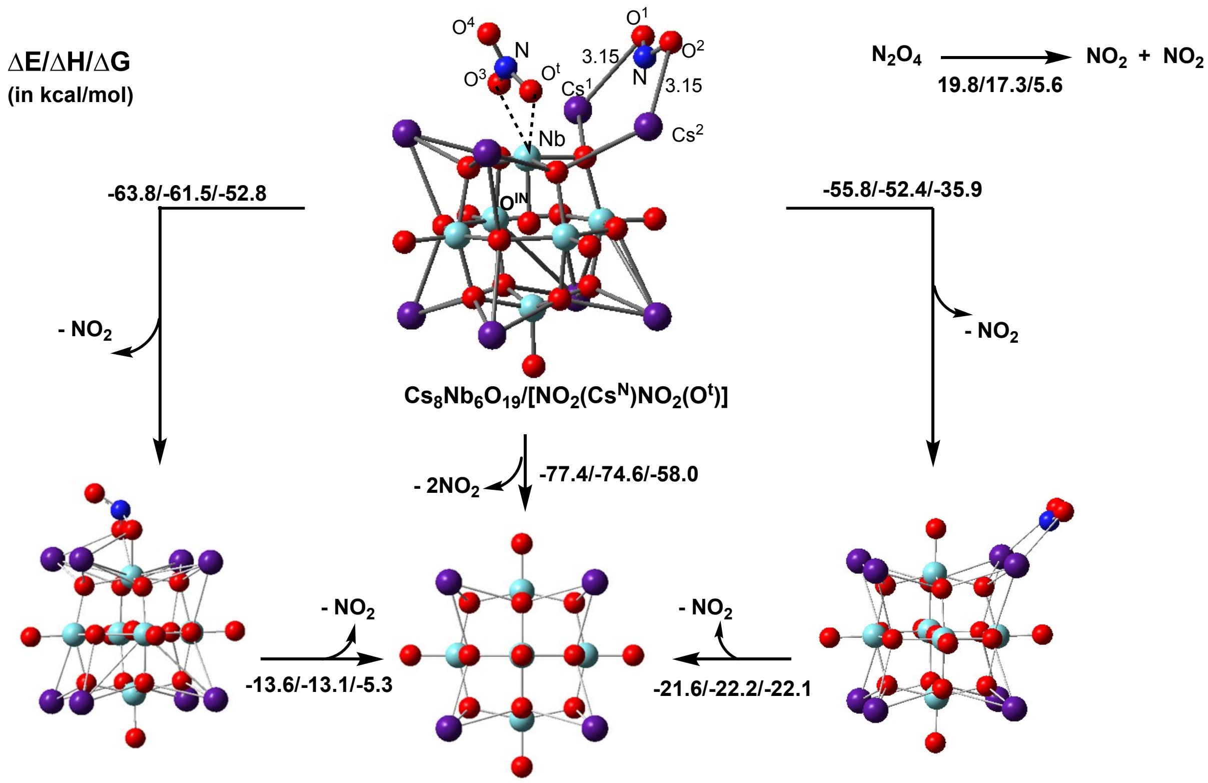

Figure S3. The calculated adsorption energies (in kcal/mol) of NO_2 radicals to $Cs_8Nb_6O_{19}$
