# Supplementary material for: Impact of ambient gases on the mechanism of [Cs8Nb6O19]-promoted nerve-agent decomposition
Source: Chem Sci. 2018 Jan 8;9(8):2147–58. doi: 10.1039/c7sc04997h (PMC5896467; doi:10.1039/c7sc04997h)
Supplement: Supplementary file 2 [file SC-009-C7SC04997H-s002.xyz]

**Supplementary Information I**

The Cartesian coordinates (in Å), total electronic energies E, enthalpies H, and Gibbs free energies G (all in Hartree), the number of imaginary frequencies Nimag and the value of the imaginary frequency ω (cm^-1^) in case the stationary point is a saddle point for all structures reported in the present work. The level of theory is M06L with 6-31++G(d,p) for S, P, F, O, N, C, H and LANL2DZ+ECP for Cs and Nb. The tabular format is:

number of atoms

label E H G Nimag [ω]

atom1 X1 Y1 Z1

3

H2O -76.4198576225 -76.394353 -76.416425 0

H 0.761239 -0.468576 0.000000

O 0.000000 0.117132 0.000000

H -0.761239 -0.468483 0.000000

2

HF -100.434514596 -100.421749 -100.441456 0

H 0.000000 0.000000 -0.828193

F 0.000000 0.000000 0.092021

18

GB -750.137783622 -749.981387 -750.027992 0

P -1.026320 -0.028487 0.153002

O 0.370642 0.616413 -0.294036

C 1.611077 0.100219 0.295668

H 1.454846 0.069010 1.381226

C 2.679913 1.102804 -0.061244

O -1.082959 -0.620665 1.505151

F -1.288203 -1.120053 -1.001257

C -2.209242 1.234592 -0.292334

H -2.027724 1.582920 -1.310038

H -3.217112 0.823090 -0.223760

H -2.120744 2.073240 0.399243

H 2.427071 2.098996 0.308312

H 2.809056 1.159622 -1.146139

H 3.634291 0.803798 0.380643

C 1.889388 -1.292004 -0.224763

H 1.107992 -1.994316 0.076709

H 1.953040 -1.290051 -1.317079

H 2.839635 -1.658180 0.174283

19

iMPA -726.112880705 -725.944397 -725.992670 0

P 1.048414 0.120177 -0.061571

C 1.673689 -1.451882 -0.658099

O 1.969714 0.328564 1.252564

O 1.022784 1.237677 -1.039918

O -0.387032 -0.223337 0.607812

C -1.578663 -0.100014 -0.225766

C -2.147101 1.292496 -0.077035

C -2.520876 -1.193221 0.219521

H 1.932977 1.241754 1.560602

H -1.286089 -0.261216 -1.274130

H -3.442843 -1.165576 -0.368139

H -2.065087 -2.179650 0.101274

H -2.783076 -1.063267 1.273817

H -3.055668 1.398752 -0.677709

H -2.404083 1.487495 0.968896

H -1.422969 2.038676 -0.410805

H 2.669856 -1.321167 -1.083113

H 1.716262 -2.177565 0.155953

H 1.008496 -1.828399 -1.438468

3

CO2 -188.580280957 -188.564871 -188.589143 0

C 0.000000 0.000000 0.000000

O 0.000000 0.000000 1.168775

O 0.000000 0.000000 -1.168775

3

SO2 -548.569938780 -548.559102 -548.588010 0

O 1.257926 -0.373573 0.000000

O -1.257926 -0.373541 0.000000

S 0.000000 0.373557 0.000000

3

NO2 -205.069466707 -205.056673 -205.083937 0

N 0.000000 0.000000 0.328094

O 0.000000 1.106294 -0.143541

O 0.000000 -1.106294 -0.143541

6

N2O4 -410.170566682 -410.140862 -410.176863 0

O -1.382552 1.104772 0.001230

O -1.382629 -1.104769 -0.001218

N -0.926122 -0.000014 -0.000017

N 0.926142 -0.000008 0.000003

O 1.382532 1.104784 -0.001224

O 1.382631 -1.104767 0.001224

12

iPOH -194.339705823 -194.224977 -194.258871 0

C 1.260940 -0.664945 0.097187

C 0.000000 0.039868 -0.370544

O -0.000002 1.412065 0.023922

C -1.260938 -0.664949 0.097187

H 0.000001 0.086019 -1.466206

H -0.000009 1.439740 0.987328

H -2.152874 -0.130251 -0.238285

H -1.309208 -1.690691 -0.282234

H -1.290251 -0.720126 1.192777

H 1.290252 -0.720123 1.192778

H 1.309214 -1.690687 -0.282234

H 2.152875 -0.130244 -0.238283

9

MPFA -632.213416092 -632.146054 -632.182565 0

C 1.667070 0.086508 -0.326852

P -0.093139 -0.095600 -0.092984

O -0.843315 -1.044392 -0.936523

O -0.613806 1.431122 -0.103852

F -0.167056 -0.446521 1.468183

H 2.070746 0.812420 0.380184

H 1.864860 0.423110 -1.345096

H 2.151147 -0.878305 -0.171896

H -1.531615 1.482580 -0.397960

33

Cs8PONb -1928.09359405 -1927.978026 -1928.106544 0

Nb 0.000000 0.000000 2.421271

O 0.000000 0.000000 4.228937

O 0.000000 0.000000 0.000000

O -1.993992 0.000000 1.993992

O 0.000000 -1.993992 1.993992

O 1.993992 0.000000 1.993992

O 0.000000 1.993992 1.993992

Nb 0.000000 0.000000 -2.421271

O 0.000000 -1.993992 -1.993992

O -1.993992 0.000000 -1.993992

O 0.000000 1.993992 -1.993992

O 1.993992 0.000000 -1.993992

O 0.000000 0.000000 -4.228937

Nb 0.000000 2.421271 0.000000

Nb -2.421271 0.000000 0.000000

Nb 0.000000 -2.421271 0.000000

Nb 2.421271 0.000000 0.000000

O 1.993992 -1.993992 0.000000

O 1.993992 1.993992 0.000000

O -1.993992 1.993992 0.000000

O -1.993992 -1.993992 0.000000

O 0.000000 4.228937 0.000000

O 4.228937 0.000000 0.000000

O 0.000000 -4.228937 0.000000

O -4.228937 0.000000 0.000000

Cs 2.770099 -2.770099 2.770099

Cs 2.770099 -2.770099 -2.770099

Cs -2.770099 -2.770099 -2.770099

Cs 2.770099 2.770099 -2.770099

Cs -2.770099 2.770099 -2.770099

Cs -2.770099 -2.770099 2.770099

Cs 2.770099 2.770099 2.770099

Cs -2.770099 2.770099 2.770099

36

Cs8PONb/CO2(Omu) -2116.70070094 -2116.569454 -2116.712880

Nb 2.241953 0.630349 0.098841

O 4.031404 0.929385 0.183177

O -0.050569 0.045990 -0.001232

O 1.602009 1.945462 -1.304870

O 1.500146 1.852739 1.534580

O 2.106367 -0.908102 1.442812

O 2.219750 -0.804613 -1.365658

Nb -2.380674 -0.482829 -0.101652

O -2.364462 0.953728 1.351406

O -2.260687 1.031092 -1.464969

O -1.577612 -1.713448 -1.503871

O -1.699696 -1.782475 1.316720

O -4.123821 -0.950025 -0.192294

Nb 0.409721 -1.508762 -1.828574

Nb -0.403728 1.816466 -1.684189

Nb -0.529613 1.716123 1.751261

Nb 0.256454 -1.614931 1.759349

O -0.172830 0.024854 2.834106

O 0.588816 -2.841712 -0.064871

O 0.031753 0.194046 -2.832454

O -0.689659 2.820510 0.057456

O 0.652409 -2.563918 -3.245345

O 0.498304 -2.736484 3.124450

O -0.873127 2.939490 3.028058

O -0.658553 3.114198 -2.906747

Cs 2.515920 0.488810 4.038957

Cs -2.911070 -0.692181 3.815782

Cs -3.647068 3.268603 -0.044549

Cs -2.304524 -4.313212 -0.183517

Cs -2.625292 -0.524343 -4.021442

Cs 1.764089 4.520486 0.208266

Cs 4.384341 -2.362753 0.079331

Cs 2.775651 0.778950 -3.835038

C 1.175933 -4.158501 -0.114536

O 0.488425 -5.055621 0.412557

O 2.279533 -4.232833 -0.688981

36

Cs8PONb/CO2(Ot) -2116.72032674 -2116.589125 -2116.732632 0

Nb 0.208317 -1.691016 -1.732913

O 0.478336 -2.939123 -2.994358

O -0.173585 0.037607 0.003947

O -0.218701 0.007687 -2.798055

O 2.008023 -0.847081 -1.371802

O 0.487270 -2.699689 0.008402

O -1.787740 -1.874963 -1.394024

Nb -0.721999 1.663540 1.761541

O 1.309140 1.918353 1.417585

O -0.968507 2.669348 -0.001027

O -2.522363 0.817885 1.408719

O -0.218135 0.017345 2.806287

O -1.146078 2.924223 2.970195

Nb -2.599791 -0.658254 0.008616

Nb -0.727449 1.657375 -1.757940

Nb 1.833698 0.614277 -0.000754

Nb 0.214740 -1.682897 1.748969

O 2.011215 -0.843005 1.381348

O -1.784424 -1.868502 1.410725

O -2.524962 0.811841 -1.401396

O 1.306744 1.914409 -1.419178

O -4.333228 -1.128213 0.011366

O 0.481538 -2.929881 3.011739

O 3.807518 1.064089 0.003193

O -1.149223 2.911568 -2.974564

Cs 3.695188 -2.923960 -0.004668

Cs 3.273419 0.736055 3.459893

Cs 1.334150 4.545746 -0.004959

Cs -2.862014 -0.729755 3.974534

Cs -3.920967 3.065169 0.003140

Cs 3.191630 0.717250 -3.505556

Cs -1.846421 -4.536160 0.016617

Cs -2.866169 -0.744041 -3.961289

C 4.850452 0.146606 -0.018779

O 5.262898 -0.243799 1.105990

O 5.231045 -0.228902 -1.158803

36

Cs8PONb/SO2(Omu) -2476.71889261 -2476.591457 -2476.736909 0

Nb 1.599210 -1.697989 -0.033941

O 3.028993 -2.819203 -0.071155

O -0.113734 -0.071044 -0.006945

O 0.453859 -2.413830 1.465294

O 0.306237 -2.509058 -1.354309

O 2.185794 -0.402727 -1.522531

O 2.335359 -0.304361 1.295705

Nb -1.888222 1.563064 0.040931

O -2.617091 0.195129 -1.293966

O -2.472016 0.294262 1.530321

O -0.532184 2.331384 1.334460

O -0.701866 2.235635 -1.463699

O -3.174810 2.830126 0.073584

Nb 1.125436 1.230524 1.682482

Nb -1.217383 -1.270487 1.828602

Nb -1.412524 -1.400728 -1.595840

Nb 0.895091 1.082837 -1.897183

O -0.308924 -0.203466 -2.842318

O 1.854685 2.151455 -0.199093

O -0.004149 0.003902 2.827518

O -2.041670 -2.135886 0.180937

O 1.880635 2.117348 3.029255

O 1.588482 1.903873 -3.324015

O -2.350814 -2.402224 -2.762645

O -2.018802 -2.186059 3.157650

Cs 1.715907 -2.071783 -3.970268

Cs -2.338382 1.715896 -3.895975

Cs -4.840930 -1.048972 0.307013

Cs 0.092013 4.851607 -0.172989

Cs -1.889614 2.014932 3.966160

Cs -0.802628 -4.823938 0.212113

Cs 4.951039 -0.201076 -0.233271

Cs 2.131885 -1.764004 3.903168

S 3.396154 2.990912 -0.555099

O 2.996876 4.442756 -0.488787

O 4.259470 2.611107 0.619383

36

Cs8PONb/SO2(Ot) -2476.74028456 -2476.612984 -2476.758797 0

O 1.295568 1.724348 -1.572963

Nb -0.706627 1.457717 -1.921341

O -2.526566 0.641776 -1.545668

Nb -2.639662 -0.643633 0.019489

O -4.374096 -1.110331 0.030868

Nb 1.863617 0.603751 -0.010931

O -0.220286 0.032663 0.006792

Nb -0.790019 1.838551 1.547359

O -2.600750 0.970840 1.256057

O 3.772171 1.256702 -0.029303

O 2.011424 -0.972784 -1.200453

Nb 0.200577 -1.855830 -1.514386

O -1.789998 -2.024664 -1.203660

O 1.230148 2.048280 1.227113

O 1.944268 -0.665978 1.517067

Nb 0.122560 -1.479783 1.918045

O -1.858740 -1.699483 1.570389

O 0.463658 -2.686765 0.330038

O 0.521217 -3.218916 -2.639650

O -0.173414 -0.306710 -2.781838

O -0.314687 0.327499 2.799688

O 0.377346 -2.561634 3.328660

O -1.079226 2.550391 -3.300670

O -1.005664 2.673578 -0.317104

O -1.209699 3.210101 2.632265

Cs -3.959996 3.034148 -0.452887

Cs 2.752586 1.090304 3.676520

Cs -3.006696 -0.292952 3.952091

Cs -2.767658 -1.181837 -3.953252

Cs 3.689021 -2.874305 0.375028

Cs 2.977306 0.237010 -3.645740

Cs -1.856549 -4.540484 0.468571

Cs 1.364394 4.510727 -0.463382

O 5.195765 -0.439057 -1.215706

O 5.287553 -0.258583 1.274680

S 5.298834 0.517155 -0.033292

36

Cs8PONb/NO2(CsN) -2133.19758522 -2133.069998 -2133.225720 0

Nb 0.188900 0.008897 -2.435772

O 0.259377 0.018137 -4.233716

O 0.205274 0.000150 -0.000689

O 1.610818 1.413750 -1.963836

O 1.606764 -1.405435 -1.976051

O -1.190089 -1.407450 -1.994485

O -1.187137 1.423931 -1.983158

Nb 0.190372 -0.009947 2.433970

O 1.607480 -1.417000 1.961795

O 1.610586 1.401430 1.974107

O -1.186046 1.411449 1.993861

O -1.191195 -1.420161 1.982383

O 0.260847 -0.018823 4.231952

Nb -1.476890 1.768025 0.007863

Nb 1.944901 1.744562 0.006977

Nb 1.940464 -1.749361 -0.009405

Nb -1.481922 -1.763326 -0.008155

O 0.224722 -2.850571 -0.011501

O -2.485475 0.003548 0.000524

O 0.231828 2.850649 0.011567

O 2.994493 -0.003661 -0.001079

O -2.774742 3.007635 0.012157

O -2.782377 -3.000070 -0.013282

O 3.251770 -2.984442 -0.015254

O 3.259325 2.976402 0.013118

Cs 0.182271 -3.903437 -2.846486

Cs 0.186781 -3.918360 2.815847

Cs 4.108895 -0.016613 2.788559

Cs -3.909362 -0.013693 2.593840

Cs 0.192570 3.902526 2.847066

Cs 4.108956 0.005822 -2.788866

Cs -3.914045 0.028110 -2.587671

Cs 0.198650 3.918706 -2.814846

N -6.166311 -0.002178 -0.009985

O -6.769982 -0.009556 1.078463

O -6.791761 -0.003148 -1.086142

36

Cs8PONb/NO2(Ot) -2133.19306455 -2133.064525 -2133.212562 0

Nb 0.790716 -1.450684 -1.686989

O 1.515206 -2.528278 -2.936570

O -0.152926 0.019766 0.009200

O 0.449220 0.271239 -2.731940

O 2.414814 -0.504014 -0.891721

O 0.820383 -2.637877 -0.030842

O -1.189034 -1.852381 -1.858160

Nb -1.174065 1.449368 1.676690

O 0.843519 1.855887 1.846773

O -1.167983 2.636612 0.032405

O -2.800844 0.512721 0.918446

O -0.778017 -0.251656 2.722823

O -1.883293 2.532250 2.934065

Nb -2.391672 -0.826335 -0.568028

Nb -0.411296 1.764495 -1.664740

Nb 1.978456 0.814434 0.555165

Nb 0.073842 -1.763668 1.647344

O 1.841565 -0.771583 1.813099

O -1.782824 -2.113829 0.873173

O -2.179736 0.764751 -1.815336

O 1.431098 2.128547 -0.888345

O -4.030492 -1.455502 -0.977438

O 0.206490 -3.058887 2.894921

O 3.659870 1.505516 0.958904

O -0.630806 3.072714 -2.886898

Cs 3.813278 -2.720080 0.506885

Cs 1.525871 0.550167 4.459142

Cs 0.992699 4.609350 0.685139

Cs -3.515316 -1.334737 3.172589

Cs -4.071515 2.756459 -0.633197

Cs 3.355829 1.257978 -3.063535

Cs -1.325986 -4.605988 -0.689268

Cs -1.801440 -0.563770 -4.481789

N 5.542620 0.461137 0.299880

O 5.888677 -0.334237 1.168225

O 5.544900 0.216266 -0.906584

39

Cs8PONb/[NO2(Ot)NO2(CsN)] -2338.35589720 -2338.210245 -2338.366937 0

Nb -1.470288 2.171538 -0.043639

O -2.314115 3.752657 -0.083720

O -0.222188 0.054782 -0.000486

O -1.223880 1.698184 -1.997207

O -3.001587 0.858077 -0.004303

O -1.210089 1.795867 1.925211

O 0.577114 2.679920 -0.067432

Nb 0.746791 -2.222359 0.052125

O -1.226460 -2.670686 0.064128

O 0.553689 -1.832949 -1.926144

O 2.326895 -0.849255 0.011625

O 0.569951 -1.739574 2.009305

O 1.476591 -3.851063 0.090858

Nb 1.583866 0.960835 -0.035616

Nb -0.363758 -0.105531 -2.469706

Nb -2.554055 -1.133771 0.039814

Nb -0.338052 0.013523 2.476383

O -2.102038 -0.874538 1.986676

O 1.401710 0.888039 1.978164

O 1.388444 0.789525 -2.031232

O -2.124955 -0.962963 -1.925149

O -0.433140 0.051286 4.265756

O -4.158608 -1.926785 0.065560

O -0.472570 -0.148381 -4.258104

Cs -4.030803 1.254782 2.825602

Cs -1.434689 -3.739194 2.895044

Cs -1.483348 -3.839724 -2.722184

Cs 3.781681 -1.139965 2.565430

Cs 3.748620 -1.347818 -2.529418

Cs -4.066736 1.132607 -2.820623

Cs 1.000836 3.741406 2.729997

Cs 0.963713 3.604720 -2.935456

N 3.838214 2.789240 -0.079673

O 3.398112 2.240111 -1.144912

O 3.411952 2.254969 1.000776

O 4.565430 3.774513 -0.090087

N 5.879370 -1.460956 0.013510

O 6.539519 -1.538737 1.087943

O 6.509239 -1.696293 -1.056259

36

Cs8PONb/H2O -2004.55261137 -2004.409975 -2004.550525 0

O 5.309469 0.006248 0.028894

O 2.699033 -0.095955 0.644524

Nb 1.213657 -0.301591 2.038731

O -0.713732 -0.400362 2.700697

Nb -2.123205 -0.180662 1.258156

O -3.660785 -0.315263 2.194559

Nb 1.965869 0.182989 -1.277627

O -0.049946 0.000675 -0.008188

Nb -0.067149 -2.396860 -0.352964

O -1.766718 -2.119184 0.735653

O 3.549161 0.310651 -2.163901

O 1.639924 2.115495 -0.739674

Nb -0.054742 2.396213 0.359543

O -1.757284 1.822882 1.323532

O 1.629725 -1.819276 -1.327582

O 0.594168 0.404478 -2.727908

Nb -1.348141 0.302325 -2.038128

O -2.806886 0.095629 -0.648654

O -1.095890 2.215021 -1.380894

O -0.042599 4.183561 0.619349

O 0.982728 1.715506 1.975482

O -1.104347 -1.708799 -1.978697

O -2.291600 0.546726 -3.558033

O 2.125587 -0.550484 3.572680

O 0.975545 -2.218094 1.375737

O -0.083163 -4.181385 -0.632005

Cs -0.947939 -3.281440 3.380600

Cs 0.852580 -2.204208 -4.170398

Cs -3.877415 -2.623174 -1.284380

Cs -0.978680 2.172636 4.179520

Cs 0.836309 3.303271 -3.363920

Cs 3.761650 2.691866 1.289615

Cs -3.866533 2.878990 -0.508166

Cs 3.755384 -2.942531 0.494855

H 4.387313 -0.054189 0.394111

H 5.071191 0.143087 -0.909869

39

Cs8PONb/H2O/CO2(Ot) -2193.17882351 -2193.019138 -2193.168017 0

O -4.527092 2.733888 0.015277

O -2.005236 1.698783 0.288509

Nb -0.198409 2.134019 1.134858

O 1.761654 1.764304 1.536200

Nb 2.532978 0.051730 0.787842

O 4.230576 0.070696 1.372067

Nb -1.902416 -0.056205 -0.737057

O 0.142120 0.000506 -0.024435

Nb 0.905007 1.184799 -2.019213

O 2.645380 1.005963 -1.016152

O -3.808601 -0.151335 -1.267524

O -2.179723 -1.002597 1.011535

Nb -0.439158 -1.185690 2.040805

O 1.562562 -0.929927 2.276328

O -1.161615 0.929481 -2.285076

O -1.304180 -1.785598 -1.535925

Nb 0.657649 -2.152385 -1.090346

O 2.441225 -1.683122 -0.267318

O 0.016740 -2.702537 0.745275

O -0.881052 -2.051879 3.550149

O -0.667654 0.773475 2.559536

O 1.079022 -0.765067 -2.545563

O 1.028518 -3.701838 -1.923654

O -0.423840 3.724050 1.932937

O 0.405321 2.676616 -0.748455

O 1.398397 2.058039 -3.511491

Cs 3.061115 3.882172 -0.181427

Cs -1.268776 -1.159124 -4.435703

Cs 4.044897 -1.084631 -2.716260

Cs 1.597537 1.117021 4.470761

Cs -2.864288 -3.803449 0.147546

Cs -3.841275 0.863934 2.716200

Cs 2.571161 -3.724640 1.938038

Cs -2.188011 3.794362 -1.908667

H -3.626041 2.368098 0.161031

H -5.041225 1.905711 -0.117234

C -4.894628 -0.688855 -0.560236

O -5.521462 0.138561 0.153354

O -5.096872 -1.910220 -0.709535

39

Cs8PONb/H2O/SO2(Ot) -2553.19625470 -2553.040790 -2553.190712 0

O -3.647265 -3.189994 -1.720651

O -1.382552 -1.790273 -1.392207

Nb 0.621288 -1.670419 -1.793158

O 2.503680 -0.926030 -1.495044

Nb 2.732878 0.511302 -0.109525

O 4.490659 0.868728 -0.166024

Nb -1.802439 -0.410513 0.080378

O 0.255789 -0.008539 -0.015745

Nb 0.764855 -1.670396 1.709445

O 2.606309 -0.941516 1.310885

O -3.783085 -0.961647 0.050995

O -1.912781 1.009707 -1.318315

Nb -0.064292 1.729543 -1.749200

O 1.944917 1.798436 -1.463300

O -1.277596 -1.766280 1.450625

O -1.832918 0.993396 1.431647

Nb 0.082041 1.737749 1.730007

O 2.049166 1.793244 1.343753

O -0.226289 2.755517 0.017656

O -0.295006 3.002378 -2.990719

O 0.219750 0.028142 -2.814920

O 0.394284 0.011097 2.781523

O -0.101024 2.977487 3.013493

O 0.977631 -2.915247 -3.032282

O 0.890058 -2.693117 -0.046764

O 1.110628 -2.953933 2.921274

Cs 3.801600 -3.287842 -0.198661

Cs -2.746634 -0.445376 3.780229

Cs 3.129159 0.562215 3.863014

Cs 2.855148 0.562661 -4.086895

Cs -3.407065 3.202054 0.168083

Cs -3.440722 -0.347391 -3.397602

Cs 2.237255 4.442703 -0.098268

Cs -1.466591 -4.523334 0.249976

H -2.752234 -2.761196 -1.738905

H -4.070865 -2.622386 -1.052218

O -5.260666 0.826612 -0.909541

O -4.971220 0.674677 1.564567

S -5.238901 -0.111872 0.290305

39

Cs8PONb/H2O/NO2(Ot) -2209.65246950 -2209.495373 -2209.647071 0

O 3.996689 3.228922 -0.888398

O 1.643688 1.936024 -0.882003

Nb -0.208526 1.726603 -1.705362

O -2.062397 0.891553 -1.905577

Nb -2.480143 -0.623717 -0.629830

O -4.156899 -1.088202 -1.092482

Nb 1.979676 0.568308 0.637645

O -0.181682 0.003666 0.012246

Nb -1.116366 1.573992 1.614248

O -2.800609 0.784433 0.808016

O 3.712973 1.141621 1.054210

O 2.337795 -0.793834 -0.772624

Nb 0.653612 -1.590061 -1.622162

O -1.342872 -1.818729 -1.844589

O 0.918052 1.782915 1.840960

O 1.658175 -0.933068 1.921733

Nb -0.219853 -1.753061 1.697982

O -2.065214 -1.933872 0.873094

O 0.507866 -2.719934 0.072665

O 1.342076 -2.764797 -2.801704

O 0.525499 0.118769 -2.713444

O -0.932544 -0.131535 2.711352

O -0.252247 -3.036925 2.961156

O -0.300884 3.015957 -2.954957

O -0.933048 2.699401 -0.058494

O -1.761313 2.751720 2.817302

Cs -3.760236 3.111246 -0.871547

Cs 1.389360 0.503596 4.510650

Cs -3.804584 -0.895548 3.067819

Cs -1.769966 -0.513701 -4.515615

Cs 3.417508 -3.156811 0.702692

Cs 3.573645 0.684075 -3.030506

Cs -1.784155 -4.508916 -0.609205

Cs 1.348971 4.531264 0.685643

H 3.098216 2.824008 -1.002590

H 4.319425 2.664277 -0.162468

O 5.478112 -0.532290 -0.599641

O 5.650122 -0.937110 1.531183

N 5.458197 -0.189521 0.579366

54

R-F -2754.72032770 -2754.419124 -2754.585216 0

F -8.386604 -0.036699 -0.378673

P -6.928243 -0.718743 -0.191003

C -7.244209 -1.932773 1.073510

O -4.207668 -2.060317 0.948887

O -6.420113 -1.192387 -1.501061

O -6.136984 0.447389 0.567526

C -6.271250 1.855291 0.134821

C -5.845060 2.029395 -1.304232

C -5.431889 2.664469 1.093178

O -2.150978 -0.458483 0.232431

Nb -1.338605 1.328993 -0.350189

O 0.142644 2.649052 -0.804247

Nb 2.022476 1.894513 -0.640415

O 3.048056 3.306786 -1.097592

Nb -0.694742 -1.895026 0.614374

O 0.625269 -0.010195 -0.002402

Nb 0.556180 -0.710322 -2.326992

O 1.715087 0.970140 -2.424633

O -1.769738 -3.290945 1.078245

O -0.432495 -0.973514 2.408307

Nb 0.715079 0.709224 2.308806

O 1.823845 2.132963 1.373996

O -0.550054 -2.136259 -1.390721

O 1.147731 -2.672952 0.792535

Nb 2.644613 -1.328033 0.334148

O 3.413867 0.456287 -0.238951

O 2.336704 -0.521654 2.176631

O 0.787950 1.235305 4.034808

O -0.935991 1.676639 1.611493

O 2.204238 -1.681035 -1.614844

O 4.128991 -2.320650 0.592347

O -2.788403 2.385165 -0.628754

O -1.058768 0.513468 -2.189850

O 0.514893 -1.239397 -4.050862

Cs -0.168249 2.877905 -3.761159

Cs 1.239212 -4.525536 -1.556781

Cs 4.399640 -0.208173 -2.990642

Cs 0.022959 4.519253 1.509668

Cs 1.421844 -2.880872 3.759373

Cs -3.124318 0.209685 3.058675

Cs 4.572459 1.449831 2.337883

Cs -3.385439 -1.455226 -2.377215

H -3.619516 -1.333247 0.623913

H -3.514942 -2.749578 1.078638

H -7.335581 2.095779 0.257723

H -5.610591 3.729251 0.913613

H -5.706639 2.448987 2.131907

H -4.365559 2.483001 0.901864

H -5.987226 3.076964 -1.589726

H -4.773733 1.814412 -1.401053

H -6.438914 1.407155 -1.979707

H -6.272995 -2.315334 1.396186

H -7.782821 -1.477557 1.906035

H -7.839547 -2.740864 0.645669

57

R-F/CO2(Ot) -2943.34523107 -2943.028194 -2943.199831 0

F -8.296709 -0.918412 0.417343

P -6.890664 -0.136303 0.638227

C -7.409882 1.558699 0.449413

O -4.378294 1.717765 0.607280

O -6.289957 -0.551232 1.928990

O -6.127645 -0.496476 -0.721168

C -6.191073 -1.849650 -1.297838

C -5.777460 -2.904274 -0.297013

C -5.289507 -1.815994 -2.508605

O -2.067617 0.354154 0.123426

Nb -1.258759 -1.447324 -0.427183

O 0.269227 -2.729583 -0.797462

Nb 2.142328 -1.990179 -0.565309

O 3.188793 -3.389775 -0.975602

Nb -0.573614 1.674605 0.559765

O 0.666458 -0.022851 0.033228

Nb 0.712426 -0.816998 2.344789

O 1.848436 -2.210592 1.449096

O -1.716055 3.256883 0.875200

O -0.422798 2.024085 -1.420902

Nb 0.725851 0.628107 -2.327408

O 1.888462 -1.054730 -2.335353

O -0.477450 0.910767 2.373599

O 1.191091 2.565616 0.821201

Nb 2.724414 1.260393 0.426301

O 3.485609 -0.537933 -0.100325

O 2.335729 1.593008 -1.529023

O 0.768769 1.100671 -4.058591

O -0.893524 -0.610998 -2.230614

O 2.297929 0.447361 2.254815

O 4.208777 2.215839 0.759883

O -2.659909 -2.541026 -0.738641

O -0.905721 -1.740266 1.557215

O 0.725190 -1.344040 4.062146

Cs -0.021036 -4.607587 1.522834

Cs 1.318801 2.873294 3.788275

Cs 4.571106 -1.493108 2.540869

Cs 0.074333 -2.952557 -3.802782

Cs 1.242166 4.558465 -1.486956

Cs -3.223589 1.556828 -2.485957

Cs 4.587834 0.150977 -2.843201

Cs -3.313040 -0.360129 2.870767

H -3.572992 1.181592 0.426600

H -4.060844 2.630486 0.403864

H -7.237905 -1.992111 -1.599571

H -5.377529 -2.761826 -3.052173

H -5.579221 -1.008251 -3.189251

H -4.243661 -1.715792 -2.191582

H -5.855764 -3.889157 -0.769543

H -4.728311 -2.757320 -0.016712

H -6.419923 -2.900781 0.587255

H -6.518024 2.183677 0.438096

H -7.975242 1.675591 -0.476195

H -8.040062 1.825114 1.299521

C -2.188016 4.257611 0.019536

O -3.337250 4.041375 -0.453867

O -1.425090 5.222970 -0.177478

57

R-F/SO2(Ot) -3303.36181947 -3303.048250 -3303.224667 0

F -8.182060 -1.375889 0.205906

P -6.819534 -0.561364 0.530984

C -7.369166 1.130349 0.412076

O -4.347396 1.409956 0.629716

O -6.247807 -1.018852 1.820611

O -5.982283 -0.806810 -0.810273

C -5.965063 -2.126389 -1.470529

C -5.568671 -3.225401 -0.512007

C -5.003922 -1.988867 -2.626119

O -2.101601 0.106273 0.040711

Nb -1.144630 -1.635252 -0.463522

O 0.485807 -2.808311 -0.779244

Nb 2.297150 -1.953188 -0.483538

O 3.442301 -3.287447 -0.840444

Nb -0.679831 1.539546 0.481957

O 0.667999 -0.090741 0.035282

Nb 0.695480 -0.833011 2.358886

O 1.974559 -2.154772 1.512637

O -2.023100 2.979567 0.894088

O -0.480961 1.866849 -1.460601

Nb 0.774616 0.527544 -2.337892

O 2.033546 -1.060163 -2.290328

O -0.597243 0.773510 2.322699

O 0.974779 2.547554 0.772707

Nb 2.632863 1.339599 0.465287

O 3.530787 -0.399755 -0.016613

O 2.283086 1.622913 -1.499675

O 0.841219 0.998592 -4.068702

O -0.788203 -0.771418 -2.263309

O 2.187298 0.542728 2.288989

O 4.039271 2.391322 0.832321

O -2.454035 -2.828420 -0.808441

O -0.817071 -1.905663 1.522289

O 0.677538 -1.368025 4.073936

Cs 0.334314 -4.684512 1.560236

Cs 0.836664 3.065392 3.663804

Cs 4.602636 -1.165512 2.694632

Cs 0.347371 -3.070089 -3.784930

Cs 0.906839 4.514194 -1.590503

Cs -3.208842 1.512392 -2.513444

Cs 4.652573 0.328562 -2.747018

Cs -3.271854 -0.756777 2.845990

H -3.626925 0.786090 0.352526

H -3.798021 2.175099 0.879593

H -6.989743 -2.281683 -1.835605

H -5.015198 -2.909072 -3.218178

H -5.295277 -1.162921 -3.284077

H -3.982162 -1.858475 -2.248987

H -5.594458 -4.182429 -1.043695

H -4.538194 -3.070731 -0.172792

H -6.253282 -3.291239 0.337920

H -6.481498 1.765217 0.434133

H -7.928371 1.279242 -0.512787

H -8.008245 1.353899 1.267703

O -2.353717 4.392183 -1.197924

O -1.098557 5.337017 0.731219

S -2.341936 4.588880 0.307629

57

R-F/NO2(Ot) -2959.81868622 -2959.503611 -2959.680443 0

F 8.300209 -0.933707 -0.411595

P 6.873921 -0.209596 -0.670593

C 7.308596 1.515880 -0.605573

O 4.259706 1.789416 -0.706743

O 6.263544 -0.721238 -1.921627

O 6.124291 -0.481336 0.715348

C 6.194551 -1.794465 1.384430

C 5.744159 -2.911029 0.471579

C 5.328794 -1.668561 2.614769

O 2.105452 0.303422 -0.130245

Nb 1.255939 -1.418004 0.557548

O -0.284368 -2.655198 1.050838

Nb -2.142834 -1.915802 0.711825

O -3.208851 -3.266127 1.240184

Nb 0.671930 1.685312 -0.726288

O -0.664907 -0.068138 -0.029486

Nb -0.667388 -1.031564 -2.257626

O -1.848772 -2.335650 -1.265951

O 1.846378 3.035046 -1.273308

O 0.449125 2.137787 1.220342

Nb -0.722188 0.816589 2.234535

O -1.903146 -0.830775 2.408782

O 0.521191 0.639397 -2.426848

O -1.118659 2.494418 -1.084851

Nb -2.667152 1.221743 -0.583228

O -3.480336 -0.503308 0.105872

O -2.313697 1.735475 1.344124

O -0.765764 1.488871 3.904304

O 0.890944 -0.421786 2.291185

O -2.259494 0.243935 -2.318405

O -4.127405 2.186660 -1.004607

O 2.659664 -2.471076 1.000107

O 0.928528 -1.932719 -1.383532

O -0.656683 -1.692589 -3.933308

Cs -0.046979 -4.738055 -1.067774

Cs -1.197416 2.439604 -4.087469

Cs -4.532264 -1.687742 -2.435270

Cs -0.125426 -2.598866 4.055267

Cs -1.300788 4.575767 1.118175

Cs 3.167617 1.663807 2.422795

Cs -4.591275 0.434382 2.750801

Cs 3.273934 -0.660287 -2.832428

H 3.581372 1.112626 -0.450223

H 3.652113 2.496801 -1.005189

H 7.249599 -1.920979 1.663553

H 5.439420 -2.568433 3.227816

H 5.634678 -0.809043 3.220840

H 4.273019 -1.601547 2.320004

H 5.843440 -3.862391 1.004849

H 4.683017 -2.782050 0.227534

H 6.351656 -2.965886 -0.435672

H 6.374060 2.081781 -0.598299

H 7.897424 1.725230 0.288498

H 7.887058 1.767159 -1.495902

O 2.668943 4.570355 1.101579

O 1.376404 5.711086 -0.227293

N 2.243046 4.873262 -0.005743

54

TS-F -2754.70944597 -2754.413083 -2754.575443 1 611i

F -7.702192 -0.604891 -0.039221

P -6.124880 -0.865554 0.502169

C -6.694595 -0.977035 2.220385

O -4.279402 -0.716852 1.377229

O -5.757848 -2.075568 -0.300008

O -5.659028 0.658343 0.090956

C -6.163697 1.318347 -1.101381

C -5.741968 0.564864 -2.346440

C -5.572536 2.710972 -1.070975

O -2.289011 -0.188169 0.042159

Nb -1.226651 1.030116 -1.246866

O 0.435676 1.883077 -2.076117

Nb 2.196057 1.277344 -1.282895

O 3.397768 2.228162 -2.231291

Nb -0.950765 -1.298242 1.294634

O 0.564776 -0.014860 0.011770

Nb 0.701130 -1.778276 -1.646024

O 2.017520 -0.430623 -2.390766

O -2.221675 -2.240251 2.192503

O -0.844985 0.417339 2.383450

Nb 0.477409 1.767876 1.635492

O 1.838992 2.492177 0.299661

O -0.643211 -2.495472 -0.293321

O 0.752254 -1.925466 2.100213

Nb 2.423834 -1.035343 1.232169

O 3.406282 0.183219 -0.029618

O 1.966447 0.596805 2.373383

O 0.354688 3.097793 2.852098

O -0.961055 2.315733 0.290390

O 2.157242 -2.325515 -0.301429

O 3.774362 -1.782048 2.163033

O -2.528376 1.831096 -2.204834

O -0.796602 -0.610925 -2.363659

O 0.797186 -3.113908 -2.853729

Cs 0.484460 0.602097 -4.759097

Cs 0.901645 -4.723373 0.958756

Cs 4.622087 -1.795354 -1.913867

Cs 0.273953 4.684735 -1.027615

Cs 0.648986 -0.591866 4.768299

Cs -3.349279 2.143792 2.026934

Cs 4.393502 2.268590 1.853623

Cs -3.209074 -2.422548 -1.903580

H -3.470253 -0.434908 0.686296

H -3.869028 -1.517693 1.780396

H -7.256481 1.355934 -1.027833

H -5.956035 3.294634 -1.913927

H -5.850515 3.236783 -0.149613

H -4.480991 2.656368 -1.178742

H -6.134169 1.068601 -3.236522

H -4.646490 0.569630 -2.415966

H -6.130034 -0.458292 -2.331382

H -6.034236 -1.615185 2.805920

H -6.690844 0.022387 2.663086

H -7.714181 -1.363026 2.235788

57

TS-F/CO2(Ot) -2943.33284176 -2943.019540 -2943.187910 1 580i

F -7.600443 -0.665560 0.331181

P -6.072595 -0.021560 0.740450

C -6.762071 1.655183 0.601107

O -4.344930 0.890935 0.852104

O -5.866875 -0.725291 2.051925

O -5.413888 -0.616919 -0.660981

C -5.889281 -1.854521 -1.256627

C -5.704197 -3.021014 -0.308232

C -5.081081 -2.027926 -2.526950

O -2.164255 0.077006 0.250436

Nb -1.172463 -1.614224 -0.398155

O 0.475548 -2.693089 -0.901132

Nb 2.267405 -1.787065 -0.727587

O 3.435565 -3.041807 -1.247046

Nb -0.705134 1.577346 0.692510

O 0.611044 0.019708 0.029975

Nb 0.876347 -0.872205 2.288041

O 2.096288 -2.107851 1.289557

O -2.064711 2.960512 1.078888

O -0.717688 1.978271 -1.280551

Nb 0.498332 0.730175 -2.301514

O 1.826119 -0.811592 -2.435185

O -0.482183 0.737255 2.446433

O 0.929729 2.630164 0.916926

Nb 2.582134 1.472934 0.362858

O 3.479724 -0.217517 -0.243781

O 2.060915 1.839209 -1.542784

O 0.385569 1.281533 -4.004482

O -0.974628 -0.662198 -2.171564

O 2.308835 0.560214 2.170723

O 3.974848 2.558075 0.679113

O -2.415123 -2.868953 -0.691492

O -0.679494 -1.927573 1.550693

O 1.020652 -1.429672 3.988357

Cs 0.444545 -4.686819 1.328826

Cs 1.224008 2.758787 3.917506

Cs 4.787504 -1.153661 2.289733

Cs 0.076720 -2.835047 -3.905438

Cs 0.596635 4.661636 -1.376393

Cs -3.458678 1.305971 -2.402198

Cs 4.383148 0.664099 -3.011958

Cs -3.061536 -0.868272 3.183801

H -3.333829 0.423463 0.520944

H -4.279490 1.854204 0.611363

H -6.951229 -1.729847 -1.496327

H -5.375979 -2.955167 -3.029586

H -5.261160 -1.205039 -3.229881

H -4.012985 -2.098186 -2.285124

H -6.071165 -3.940834 -0.778715

H -4.638507 -3.148491 -0.086760

H -6.261810 -2.856579 0.616430

H -6.288140 2.322539 1.320965

H -6.555771 2.056490 -0.395111

H -7.839641 1.623326 0.756467

C -2.776323 3.803527 0.221092

O -3.926999 3.385922 -0.092790

O -2.197834 4.843965 -0.142090

57

TS-F/SO2(Ot) -3303.34840823 -3303.039241 -3303.210693 1 438i

F 7.624229 -1.517030 -0.019269

P 6.232258 -0.681023 -0.402052

C 6.963252 0.926821 -0.017845

O 4.389124 0.533770 -0.349362

O 5.973679 -1.100383 -1.811576

O 5.386912 -1.280222 0.856590

C 5.576334 -2.650825 1.320204

C 5.269297 -3.646512 0.222778

C 4.640458 -2.793242 2.501336

O 2.133570 -0.259632 -0.120052

Nb 0.906424 -1.908995 0.263817

O -0.865208 -2.843835 0.531498

Nb -2.545958 -1.733686 0.353634

O -3.854904 -2.922387 0.643747

Nb 0.819713 1.408897 -0.393090

O -0.655523 -0.054615 -0.053451

Nb -0.845118 -0.568731 -2.436401

O -2.289329 -1.783043 -1.637972

O 2.382247 2.614675 -0.863688

O 0.768034 1.595006 1.557154

Nb -0.662419 0.355655 2.366509

O -2.117014 -1.024174 2.219262

O 0.643931 0.800270 -2.305377

O -0.643215 2.654776 -0.565620

Nb -2.462570 1.655781 -0.311984

O -3.556834 0.019735 0.059774

O -1.995545 1.729551 1.659529

O -0.619329 0.673002 4.130928

O 0.711057 -1.127361 2.126504

O -2.140510 0.977021 -2.192011

O -3.727369 2.907431 -0.499343

O 2.026408 -3.295063 0.473730

O 0.507551 -1.927100 -1.714964

O -0.961669 -1.011936 -4.169323

Cs -1.011313 -4.475058 -2.026928

Cs 0.036369 3.699931 -3.224679

Cs -4.737782 -0.384655 -2.717655

Cs -0.691980 -3.377339 3.499844

Cs -0.255918 4.415374 1.897218

Cs 3.373720 0.781081 2.626241

Cs -4.539959 0.672247 2.847456

Cs 3.128435 -1.006928 -2.981936

H 3.290857 0.075821 -0.228814

H 4.257873 1.387014 -0.783922

H 6.620716 -2.746255 1.642035

H 4.719490 -3.802464 2.917533

H 4.899071 -2.085662 3.298244

H 3.602260 -2.644556 2.178033

H 5.420052 -4.663355 0.601936

H 4.219351 -3.552343 -0.077905

H 5.928532 -3.499896 -0.636660

H 8.048668 0.856511 -0.104426

H 6.578942 1.685316 -0.697754

H 6.699881 1.218118 1.000250

O 2.865791 3.962661 1.230956

O 1.653455 5.004083 -0.683092

S 2.849653 4.153962 -0.273226

57

TS-F/NO2(Ot) -2959.80569825 -2959.495633 -2959.669058 1 568i

F -7.808756 -0.690463 0.387141

P -6.296300 -0.050781 0.698187

C -6.843885 1.650249 0.424818

O -4.347533 0.924339 0.592345

O -6.005957 -0.583652 2.063634

O -5.624045 -0.680884 -0.650632

C -5.978100 -2.016203 -1.118775

C -5.525026 -3.064995 -0.124109

C -5.289511 -2.168613 -2.457262

O -2.158609 0.006190 0.204923

Nb -1.106859 -1.590440 -0.598154

O 0.541637 -2.597400 -1.180305

Nb 2.312412 -1.660489 -0.843347

O 3.521222 -2.832646 -1.473536

Nb -0.840847 1.545371 0.852093

O 0.641065 -0.029318 0.022466

Nb 0.822272 -1.096276 2.192856

O 2.120529 -2.215174 1.111299

O -2.173674 2.658586 1.502862

O -0.746208 2.103419 -1.078499

Nb 0.542403 0.966213 -2.182584

O 1.895360 -0.526518 -2.474637

O -0.531979 0.418230 2.479769

O 0.848258 2.517236 1.210329

Nb 2.514110 1.448524 0.592982

O 3.490604 -0.136392 -0.191215

O 2.063721 2.021375 -1.296195

O 0.443864 1.723772 -3.812013

O -0.907187 -0.450312 -2.266487

O 2.260910 0.343664 2.282104

O 3.869367 2.541711 1.045946

O -2.386413 -2.764856 -1.075166

O -0.689703 -2.134095 1.309161

O 0.931939 -1.839596 3.829364

Cs 0.586945 -4.786675 0.875383

Cs 1.052942 2.287540 4.206178

Cs 4.764379 -1.302146 2.247194

Cs 0.266937 -2.409865 -4.172413

Cs 0.768025 4.696899 -0.926521

Cs -3.412047 1.285660 -2.389931

Cs 4.445392 1.001795 -2.794212

Cs -3.128492 -1.200286 2.910490

H -3.340089 0.405934 0.394607

H -4.104320 1.701440 1.118052

H -7.066952 -2.046740 -1.240786

H -5.559261 -3.132243 -2.901704

H -5.604319 -1.382093 -3.153201

H -4.200734 -2.158872 -2.319896

H -5.791985 -4.061677 -0.492776

H -4.432769 -3.025173 -0.027651

H -6.006465 -2.918077 0.847182

H -6.340432 2.324026 1.116317

H -6.583314 1.960452 -0.588755

H -7.925359 1.705500 0.556817

O -3.954843 3.900604 -0.485083

O -2.221450 5.159983 -0.091810

N -3.065161 4.333571 0.228676

54

P5-F -2754.73113078 -2754.430748 -2754.595440 0

F 7.226826 -0.917217 0.269840

P 5.909701 -0.403373 -0.813932

C 7.238016 0.320515 -1.840789

O 4.726140 0.478786 -1.749802

O 5.359671 -1.802995 -0.997522

O 5.364437 0.590922 0.451859

C 5.778924 0.493410 1.827581

C 5.289110 -0.800276 2.449737

C 5.181140 1.703285 2.521754

O 2.329870 -0.221594 0.081817

Nb 1.030458 0.183292 1.831790

O -0.655356 0.452204 2.827041

Nb -2.353397 0.376392 1.649933

O -3.662570 0.664676 2.846801

Nb 1.081945 -0.391013 -1.744850

O -0.541064 -0.002625 0.003899

Nb -0.717820 -2.361794 0.380233

O -2.123745 -1.633795 1.682727

O 2.350978 -0.671613 -2.979610

O 0.978072 1.614925 -1.671518

Nb -0.408681 2.354796 -0.368814

O -1.857240 2.258483 1.055485

O 0.716218 -2.245017 -1.049297

O -0.567919 -0.456092 -2.830783

Nb -2.298792 -0.165217 -1.730157

O -3.402955 0.173159 -0.065481

O -1.832414 1.807731 -1.729294

O -0.246094 4.127667 -0.664595

O 0.959123 2.064058 1.115611

O -2.081928 -2.081224 -1.113644

O -3.568167 -0.287274 -2.996498

O 2.209134 0.332500 3.174201

O 0.706983 -1.798134 1.727760

O -0.857690 -4.134141 0.673125

Cs -0.757379 -2.193614 4.295729

Cs -0.672569 -3.416216 -3.423030

Cs -4.640756 -2.543849 0.347824

Cs -0.385815 3.376284 3.450194

Cs -0.302539 2.157497 -4.304340

Cs 3.422058 2.936235 -0.514794

Cs -4.309909 2.993223 -0.534301

Cs 3.172549 -3.205417 0.454788

H 3.258905 0.040929 -0.013716

H 4.148690 -0.146761 -2.223362

H 6.872534 0.532431 1.868127

H 5.463826 1.711994 3.579597

H 5.548009 2.634919 2.072157

H 4.084458 1.663672 2.487136

H 5.603363 -0.863872 3.498067

H 4.191906 -0.821002 2.439403

H 5.716533 -1.647535 1.906661

H 6.934565 0.301761 -2.888466

H 7.363390 1.368791 -1.550456

H 8.183414 -0.198828 -1.690051

57

P5-F/CO2(Ot) -2943.33979502 -2943.022898 -2943.194690 0

F -7.649215 -0.695192 0.238291

P -6.110977 -0.000744 0.729348

C -6.953916 1.614799 0.564626

O -4.538718 0.836763 0.892701

O -5.999620 -0.779730 2.020761

O -5.446977 -0.627616 -0.691378

C -5.924953 -1.846026 -1.299896

C -5.743432 -3.024260 -0.363409

C -5.116009 -2.013452 -2.573063

O -2.151693 0.030336 0.178055

Nb -1.082752 -1.693520 -0.482031

O 0.566273 -2.718749 -0.934393

Nb 2.350581 -1.757140 -0.705279

O 3.555313 -2.980474 -1.204685

Nb -0.696829 1.606637 0.683444

O 0.609146 0.024274 0.025492

Nb 0.833680 -0.881221 2.269852

O 2.107253 -2.091843 1.290104

O -2.097423 2.921359 1.048062

O -0.703684 1.975443 -1.292206

Nb 0.549133 0.747971 -2.286389

O 1.902292 -0.784036 -2.402792

O -0.526354 0.710804 2.416255

O 0.900951 2.658220 0.947196

Nb 2.588245 1.490278 0.418092

O 3.521112 -0.177993 -0.185477

O 2.087772 1.868266 -1.485927

O 0.502889 1.307798 -3.988052

O -0.901217 -0.672864 -2.208171

O 2.264063 0.568254 2.198277

O 3.958129 2.587516 0.772263

O -2.312562 -2.940882 -0.812881

O -0.681041 -1.963028 1.489939

O 0.971511 -1.454482 3.963671

Cs 0.501057 -4.707409 1.327447

Cs 1.099867 2.720583 3.985278

Cs 4.748807 -1.119231 2.414962

Cs 0.216693 -2.790318 -3.981438

Cs 0.582922 4.689045 -1.374980

Cs -3.500395 1.263450 -2.375275

Cs 4.436828 0.744531 -2.953392

Cs -3.181818 -0.929842 3.066581

H -3.108699 0.217396 0.378948

H -4.493179 1.778829 0.625732

H -6.986764 -1.725946 -1.540758

H -5.394881 -2.945039 -3.077509

H -5.305866 -1.193378 -3.277713

H -4.045721 -2.067393 -2.336543

H -6.110688 -3.942849 -0.837204

H -4.678409 -3.157472 -0.139293

H -6.299128 -2.856749 0.562034

H -6.468223 2.383166 1.168117

H -6.904664 1.930971 -0.482729

H -8.003483 1.513296 0.837172

C -2.803967 3.798296 0.190268

O -3.938112 3.390537 -0.154455

O -2.196971 4.840780 -0.118181

57

P5-F/SO2(Ot) -3303.36242420 -3303.049506 -3303.226601 0

F 7.207137 -1.086311 -0.957860

P 5.916094 -0.092577 -0.294398

C 7.198858 0.662081 0.764125

O 4.659796 0.662276 0.704677

O 5.498385 0.494326 -1.626881

O 5.236460 -1.507023 0.334447

C 5.485044 -2.823093 -0.192619

C 4.982698 -2.931588 -1.620095

C 4.756921 -3.776214 0.736310

O 2.198289 -0.379604 -0.416183

Nb 0.783042 -2.037898 -0.457532

O -1.016496 -2.883913 -0.379633

Nb -2.592338 -1.645036 -0.023102

O -3.989519 -2.763512 -0.013289

Nb 1.033992 1.482096 0.017369

O -0.579374 -0.051950 -0.019087

Nb -0.922631 0.232645 -2.398376

O -2.418307 -1.083360 -1.951788

O 2.590047 2.631421 -0.039614

O 1.094050 0.888095 1.908342

Nb -0.402018 -0.437047 2.350546

O -1.988742 -1.619849 1.913928

O 0.649072 1.424514 -1.938400

O -0.367042 2.720123 0.345465

Nb -2.257687 1.732311 0.412146

O -3.480163 0.158164 0.330069

O -1.677035 1.147838 2.261875

O -0.156217 -0.719857 4.110298

O 0.787055 -1.885990 1.550508

O -2.107594 1.693316 -1.595202

O -3.395014 3.076183 0.731711

O 1.772837 -3.495691 -0.756474

O 0.378841 -1.333663 -2.293243

O -1.171993 0.449503 -4.161149

Cs -1.338062 -3.540021 -3.338885

Cs -0.482900 4.276641 -2.269084

Cs -4.842502 0.680288 -2.346607

Cs -0.726804 -4.388833 2.204539

Cs 0.135947 3.513220 3.247067

Cs 3.376625 -0.915844 2.899009

Cs -4.236548 -0.092817 3.254727

Cs 3.075307 0.172126 -3.301438

H 3.084282 -0.360354 -0.008216

H 4.340897 1.457887 0.252491

H 6.562914 -3.017041 -0.166035

H 4.915685 -4.812019 0.418134

H 5.131761 -3.682593 1.763681

H 3.675640 -3.591060 0.704794

H 5.175369 -3.935148 -2.017037

H 3.897566 -2.771718 -1.643643

H 5.502601 -2.201902 -2.247299

H 6.927971 1.693913 0.993001

H 7.237968 0.109475 1.708632

H 8.178105 0.608451 0.290066

O 2.124886 5.004582 -0.921404

O 2.335847 4.667786 1.525647

S 2.950168 4.397437 0.179294

57

P5-F/NO2(Ot) -2959.82205087 -2959.507368 -2959.682688 0

F 7.118035 -1.678028 -0.590874

P 5.867641 -0.445742 -0.640356

C 7.201591 0.781224 -0.410620

O 4.689627 0.835700 -0.309444

O 5.321425 -0.863544 -1.992285

O 5.255216 -1.079682 0.798232

C 5.472290 -2.433557 1.240171

C 4.879407 -3.424025 0.256055

C 4.799879 -2.528399 2.596381

O 2.135534 -0.620196 -0.258427

Nb 0.677766 -1.873048 0.820071

O -1.097760 -2.412891 1.499116

Nb -2.629362 -1.106374 1.009125

O -4.058871 -1.893955 1.757662

Nb 1.119194 1.176474 -1.077652

O -0.655149 -0.018451 -0.021793

Nb -1.074454 -1.232771 -2.046612

O -2.556905 -1.931684 -0.835491

O 2.552525 1.996202 -1.829730

O 1.187401 1.857202 0.793296

Nb -0.289567 1.177098 2.019261

O -1.916824 0.069103 2.507994

O 0.548571 -0.098427 -2.517871

O -0.343353 2.414630 -1.524586

Nb -2.178675 1.779375 -0.782775

O -3.459747 0.543306 0.185866

O -1.542467 2.445222 1.022149

O 0.061874 2.072704 3.544962

O 0.842131 -0.501096 2.281433

O -2.195067 0.458066 -2.316552

O -3.282600 3.078047 -1.344273

O 1.686026 -3.227382 1.419763

O 0.195265 -2.470153 -1.040340

O -1.398778 -2.121023 -3.578113

Cs -1.613576 -4.763416 -0.340483

Cs -0.585589 1.840162 -4.486827

Cs -4.955461 -0.678151 -2.123872

Cs -0.705056 -1.902317 4.436586

Cs 0.457789 4.700230 0.370987

Cs 3.532432 1.051230 2.498579

Cs -4.057083 2.183678 2.635800

Cs 2.767720 -2.208139 -2.782067

H 3.053314 -0.442131 0.004458

H 4.250474 1.101738 -1.134857

H 6.550848 -2.603211 1.333297

H 4.937921 -3.528821 3.019554

H 5.232552 -1.806371 3.300194

H 3.718259 -2.367131 2.497943

H 5.052536 -4.450354 0.599963

H 3.793282 -3.277750 0.198089

H 5.351296 -3.299229 -0.722410

H 6.980071 1.661465 -1.017966

H 7.196698 1.100329 0.636528

H 8.180523 0.372306 -0.657161

O 4.769481 4.081206 -1.357996

O 3.694148 3.854316 0.534734

N 4.166691 3.455316 -0.521390

54

TS2-F -2754.71986249 -2754.421892 -2754.58334 1 90i

C -7.610659 -1.104883 1.351435

P -6.240800 -0.494284 0.299477

O -6.457561 -1.228727 -1.000629

O -4.764606 0.640734 -0.694322

C -5.246880 1.538711 -1.662258

C -4.640222 1.282693 -3.042728

F -6.727559 1.072199 0.613615

O -5.069864 -0.811294 1.361021

C -4.972186 2.985012 -1.250467

O -2.869840 -2.090051 0.881972

Nb -1.234056 -1.298500 0.636583

O -0.780478 -2.165894 -1.143546

Nb 0.834995 -1.344182 -2.019667

O 1.941589 -2.374326 -0.593679

Nb 2.044677 -1.550259 1.226470

O 1.522700 -0.172803 2.615324

Nb 0.355189 1.374779 1.939687

O 0.167973 2.377974 3.425529

O 0.094545 -2.391777 1.545060

O -2.160064 0.297129 -0.584983

Nb -0.675536 1.632883 -1.326416

O -1.588273 2.865888 -2.244182

O 0.551168 -0.020325 -0.014220

Nb 2.645978 1.260800 -0.629382

O 2.031289 2.144303 1.096250

O -1.208041 0.057602 2.106579

O 3.037323 -2.714223 2.172213

O 3.398116 -0.252976 0.520140

O -0.305815 0.176772 -2.676491

O 1.172600 2.343628 -1.552143

O -0.711891 2.390620 0.560412

O 1.169513 -2.380969 -3.450968

O 2.434066 -0.055730 -2.126567

O 4.133182 2.170088 -1.056064

Cs 4.125770 1.261352 3.030829

Cs 0.330558 -4.836147 -0.229138

Cs -3.622031 -1.945548 -2.280164

Cs 4.676919 -1.949034 -1.691381

Cs 1.511902 1.653546 -4.487407

Cs 0.919031 4.838639 0.111147

Cs -0.309599 -1.633034 4.439432

Cs -3.526689 1.980876 1.867354

H -3.140492 0.466494 -0.638907

H -4.221275 -1.279905 1.056912

H -6.339759 1.415550 -1.759385

H -5.349448 3.698006 -1.994252

H -5.471718 3.208247 -0.300485

H -3.888780 3.152096 -1.166134

H -4.904252 2.081319 -3.746136

H -3.543318 1.257152 -2.985877

H -5.019722 0.346858 -3.468934

H -7.538936 -2.191722 1.438136

H -7.575998 -0.668462 2.353755

H -8.571058 -0.857655 0.891864

57

TS2-F/CO2(Ot) -2943.33122020 -2943.016324 -2943.186150 1 128i

F -6.521139 0.044646 -1.089441

P -6.130958 0.001579 0.526029

C -7.345929 1.320748 0.871543

O -4.772361 0.795493 0.845274

O -6.509983 -1.149115 1.412717

O -4.628800 -1.301453 -0.384796

C -5.132596 -2.496463 -0.901674

C -4.731253 -3.698740 -0.047606

C -4.695151 -2.695645 -2.353508

O -2.138294 -0.473830 -0.152753

Nb -0.701601 -1.939613 -0.698302

O 1.150829 -2.659318 -0.949029

Nb 2.698554 -1.428834 -0.517423

O 4.138982 -2.427663 -0.870517

Nb -1.012002 1.350342 0.513323

O 0.594229 0.029686 0.028527

Nb 0.697274 -0.884453 2.268506

O 2.281343 -1.816999 1.436818

O -2.542376 2.570467 0.779826

O -0.889365 1.741686 -1.441730

Nb 0.677887 0.773009 -2.296600

O 2.283036 -0.494670 -2.245035

O -0.916432 0.451089 2.256636

O 0.337049 2.634060 0.966911

Nb 2.257047 1.774108 0.641580

O 3.524974 0.325608 0.126504

O 1.876369 2.112734 -1.303660

O 0.767712 1.369410 -3.982217

O -0.498029 -0.870178 -2.386957

O 1.875943 0.796196 2.370842

O 3.357300 3.101186 1.129426

O -1.615895 -3.384076 -1.197157

O -0.491155 -2.191261 1.316970

O 0.749633 -1.466243 3.963965

Cs 1.140521 -4.695182 1.280158

Cs 0.165210 2.698541 3.983459

Cs 4.542485 -0.497324 2.887334

Cs 1.111746 -2.756175 -4.015270

Cs 0.090226 4.658663 -1.333954

Cs -3.625050 0.842740 -2.541097

Cs 4.546654 1.475033 -2.518802

Cs -3.412802 -1.493631 2.544222

H -3.093551 -0.780206 -0.221453

H -4.707505 1.740447 0.473287

H -6.238202 -2.454157 -0.890068

H -5.048439 -3.651289 -2.762755

H -5.110006 -1.901200 -2.988384

H -3.599074 -2.690292 -2.418937

H -5.115339 -4.640696 -0.461463

H -3.637150 -3.778351 -0.002207

H -5.154265 -3.585803 0.957551

H -7.314024 1.569456 1.935106

H -7.133139 2.224123 0.292730

H -8.352515 0.971945 0.629125

C -3.275078 3.487675 0.038717

O -4.433245 3.089029 -0.285481

O -2.727732 4.570872 -0.230084

57

TS2-F/SO2(Ot) -3303.35679851 -3303.045512 -3303.216209 1 76i

F -6.299911 2.402310 0.643276

P -6.131654 0.838204 0.098071

C -7.620098 0.349774 1.038786

O -5.092412 0.111109 1.115389

O -6.434605 0.390107 -1.306551

O -4.385853 1.749134 -0.664623

C -4.619922 2.876410 -1.475717

C -3.955620 2.743429 -2.845032

C -4.132262 4.151911 -0.790137

O -1.895213 0.976005 -0.533688

Nb -0.158400 2.155093 -0.929377

O 1.815503 2.518057 -0.951195

Nb 3.034067 1.032483 -0.309501

O 4.668198 1.734625 -0.492073

Nb -1.165013 -0.951068 0.275733

O 0.655838 0.020767 -0.012691

Nb 0.876430 -0.852231 -2.294020

O 2.671885 0.110720 -2.050879

O -3.091437 -1.550802 0.469443

O -1.137671 -0.005512 2.034749

Nb 0.652841 0.962175 2.236566

O 2.484257 1.538987 1.576456

O -0.938607 -1.482606 -1.629650

O -0.228597 -2.455641 0.912726

Nb 1.883033 -1.994340 0.856995

O 3.435731 -0.833026 0.475196

O 1.446559 -0.910742 2.505159

O 0.618220 1.672785 3.882649

O -0.124330 2.438522 1.083765

O 1.665780 -2.337559 -1.110542

O 2.631763 -3.484277 1.503037

O -0.768686 3.707423 -1.555863

O 0.076417 0.981894 -2.536348

O 1.112010 -1.513692 -3.938313

Cs 2.185454 2.449941 -4.008190

Cs -0.647098 -4.541474 -1.192645

Cs 4.525684 -2.240770 -2.053415

Cs 1.988446 4.522996 1.282111

Cs -1.083016 -2.584425 3.728351

Cs -3.068136 2.385937 2.161701

Cs 4.246251 -0.131809 3.331986

Cs -3.789358 -0.804255 -2.649485

H -2.837603 1.301441 -0.619259

H -4.315262 -0.368941 0.728759

H -5.705601 2.975115 -1.647273

H -4.330119 5.041673 -1.400601

H -4.651157 4.286740 0.165689

H -3.045033 4.106121 -0.635142

H -4.020528 3.681910 -3.408042

H -2.888647 2.504508 -2.742147

H -4.453402 1.976458 -3.448854

H -7.760619 -0.729524 0.945266

H -7.533384 0.602994 2.098942

H -8.498390 0.851511 0.625897

O -3.647262 -3.545141 -0.993278

O -2.823404 -3.957755 1.310571

S -3.798449 -3.166527 0.465384

57

TS2-F/NO2(Ot) -2959.81450326 -2959.502392 -2959.673861 1 84i

C 7.714112 0.083210 1.074283

P 6.237249 -0.406346 0.109333

O 6.521896 0.149720 -1.263317

O 4.560874 -1.367738 -0.718408

C 4.878559 -2.406360 -1.615438

C 4.311716 -2.150846 -3.010624

F 6.489878 -1.994084 0.564004

O 5.175282 0.207436 1.164615

C 4.376654 -3.751176 -1.092737

O 3.039376 1.620421 0.614366

Nb 1.252496 1.034640 0.409095

O 0.896577 1.774148 -1.437115

Nb -0.847936 1.068768 -2.180225

O -1.761887 2.367031 -0.853184

Nb -1.906262 1.797052 1.058741

O -1.459211 0.556365 2.596389

Nb -0.548779 -1.215183 2.095428

O -0.446452 -2.085520 3.666324

O 0.159401 2.400911 1.202399

O 2.002620 -0.758948 -0.642543

Nb 0.332910 -1.985585 -1.160453

O 1.040179 -3.416007 -1.957731

O -0.622827 -0.032454 -0.025512

Nb -2.905844 -1.112905 -0.437649

O -2.329572 -1.839814 1.380011

O 1.175232 -0.115721 2.040741

O -2.708241 3.191598 1.853706

O -3.421129 0.603810 0.553271

O 0.049600 -0.660200 -2.651916

O -1.603530 -2.464092 -1.238781

O 0.347368 -2.493724 0.806643

O -1.120927 1.945335 -3.720734

O -2.607783 -0.005708 -2.075649

O -4.500413 -1.882379 -0.708860

Cs -4.212839 -0.459814 3.279596

Cs 0.151432 4.678033 -0.787440

Cs 3.780111 1.280946 -2.479981

Cs -4.582259 2.208068 -1.822952

Cs -1.994569 -2.101030 -4.234980

Cs -1.642606 -4.719583 0.757473

Cs 0.782632 2.051291 4.085243

Cs 3.269566 -2.302485 1.983136

H 2.962920 -1.022610 -0.694528

H 4.360271 0.689459 0.826901

H 5.974906 -2.468624 -1.718772

H 4.627594 -4.570298 -1.778132

H 4.843269 -3.980447 -0.127348

H 3.281583 -3.739341 -0.998150

H 4.441501 -3.025439 -3.658944

H 3.234244 -1.942512 -2.963478

H 4.837491 -1.317003 -3.488727

H 7.813567 1.170921 1.049819

H 7.643424 -0.240342 2.116547

H 8.609873 -0.355853 0.628374

N 3.373273 3.805261 0.504613

O 3.419999 4.136872 -0.675962

O 2.735046 4.362602 1.383434

54

Cs8PONb-MPFA-iPOH -2754.77587829 -2754.475734 -2754.644150 0

C -7.559729 -2.939859 0.227267

P -6.209652 -1.766669 -0.055464

O -6.194013 -1.288877 -1.478287

O -4.694800 1.800627 -0.193792

C -5.685069 2.814004 -0.180850

C -5.377824 3.883059 -1.219864

F -6.776171 -0.533029 0.899383

O -4.952544 -2.231225 0.648878

C -5.816700 3.412154 1.213732

O -2.387517 -3.004028 -0.110624

Nb -0.982135 -1.630668 -0.055554

O -0.721040 -1.395394 -2.034275

Nb 0.697359 0.013487 -2.435318

O 2.081297 -1.423147 -1.944568

Nb 2.378610 -1.739473 0.032306

O 1.981287 -1.455949 1.999554

Nb 0.575955 -0.029719 2.441951

O 0.608220 -0.041407 4.238434

O 0.602236 -2.825352 -0.026263

O -2.089804 0.008219 -0.069661

Nb -1.007746 1.731323 -0.026915

O -2.303492 3.015289 -0.028976

O 0.611847 -0.056428 0.001870

Nb 2.405029 1.686028 0.065634

O 2.013828 1.359517 2.024762

O -0.830263 -1.419310 1.935899

O 3.622163 -3.039341 0.052862

O 3.475555 -0.045755 0.078563

O -0.701660 1.411809 -2.007589

O 0.683936 2.802737 0.025642

O -0.790297 1.382162 1.959377

O 0.839757 0.035829 -4.226321

O 2.108446 1.393656 -1.913907

O 3.692226 2.937604 0.108496

Cs 4.468238 -0.085027 2.940924

Cs 0.661870 -3.897534 -2.859721

Cs -3.589173 -0.052912 -2.617554

Cs 4.598166 -0.027695 -2.732659

Cs 0.709763 3.902304 -2.805722

Cs 0.567580 3.868273 2.857731

Cs 0.483105 -3.945296 2.777642

Cs -3.691257 0.025629 2.414892

H -3.813507 2.258224 -0.126977

H -3.329344 -2.821298 0.096146

H -6.625524 2.310492 -0.442769

H -6.578192 4.199749 1.252415

H -6.099590 2.639018 1.936243

H -4.857370 3.846310 1.522736

H -6.148069 4.662534 -1.239320

H -4.409356 4.346746 -0.998898

H -5.317345 3.441472 -2.220620

H -7.352866 -3.860246 -0.323759

H -7.646249 -3.181276 1.288971

H -8.505293 -2.526442 -0.129535

57

Cs8PONb-MPFA-iPOH/CO2(Ot) -2943.38695264 -2943.068205 -2943.243528 0

F -6.852438 0.142643 -1.015016

P -6.550276 0.710368 0.511426

C -7.884189 1.927772 0.595760

O -5.201445 1.399641 0.405253

O -6.739932 -0.410423 1.488309

O -4.524555 -1.998077 -0.440954

C -5.566994 -2.960943 -0.522048

C -5.568852 -3.855349 0.707889

C -5.451327 -3.763531 -1.809607

O -1.975035 -0.176544 -0.002988

Nb -0.862985 -1.844002 -0.288917

O 0.885548 -2.809381 -0.453130

Nb 2.575576 -1.673670 -0.277589

O 3.895202 -2.864975 -0.496562

Nb -0.816565 1.400120 0.281699

O 0.699039 0.025354 0.029470

Nb 0.772680 -0.463867 2.441918

O 2.218789 -1.693657 1.719719

O -2.382213 2.795115 0.502070

O -0.730812 1.624994 -1.709710

Nb 0.723980 0.369446 -2.414698

O 2.182084 -1.030989 -2.149010

O -0.714313 0.962682 2.215799

O 0.667968 2.720506 0.483845

Nb 2.461466 1.729098 0.298085

O 3.580911 0.066379 0.019820

O 2.085189 1.723213 -1.686216

O 0.825603 0.634575 -4.182106

O -0.603773 -1.134091 -2.170979

O 2.099046 1.064382 2.195560

O 3.704735 3.002565 0.524781

O -2.073594 -3.179082 -0.492023

O -0.575919 -1.785770 1.721105

O 0.874029 -0.796149 4.198662

Cs 0.861810 -4.352530 2.162564

Cs 0.546825 3.418154 3.384793

Cs 4.670690 -0.345467 2.804872

Cs 0.844031 -3.410835 -3.440458

Cs 0.516439 4.343806 -2.105075

Cs -3.693569 0.441134 -2.323764

Cs 4.632099 0.592594 -2.776783

Cs -3.609955 -0.595555 2.408733

H -3.660950 -2.482332 -0.467842

H -4.542987 2.851716 -0.081778

H -6.494158 -2.375426 -0.532564

H -6.272712 -4.481532 -1.914524

H -5.471102 -3.098953 -2.680532

H -4.504513 -4.316823 -1.828130

H -6.351255 -4.620493 0.648752

H -4.600667 -4.362125 0.808689

H -5.756907 -3.257502 1.604736

H -7.853528 2.425740 1.567458

H -7.768285 2.682351 -0.185438

H -8.854031 1.438281 0.487255

C -2.849039 3.748397 -0.258248

O -4.163037 3.658223 -0.527137

O -2.196140 4.683511 -0.721544

57

PONb-MPFA-iPOH/SO2(Ot) -3303.39439917 -3303.080933 -3303.258183 0

F -6.673601 0.533067 0.957531

P -6.278709 -0.734464 -0.031945

C -7.727825 -1.765934 0.290311

O -5.051439 -1.340004 0.624600

O -6.253357 -0.240385 -1.447171

O -4.370081 2.440214 -0.165707

C -5.325419 3.487766 -0.207813

C -4.956540 4.513496 -1.269275

C -5.472836 4.129653 1.164811

O -2.000461 0.460704 -0.058462

Nb -0.731911 2.031631 -0.041487

O 1.105798 2.864278 -0.004567

Nb 2.682308 1.576386 0.041551

O 4.109834 2.658226 0.072509

Nb -0.978917 -1.262159 -0.040027

O 0.657756 0.019924 -0.002719

Nb 0.780177 0.088175 -2.468730

O 2.342256 1.275096 -1.928307

O -2.749817 -2.439385 -0.143647

O -0.901252 -1.113789 1.954544

Nb 0.665767 0.093968 2.461557

O 2.252563 1.284222 1.992205

O -0.797022 -1.125458 -2.027706

O 0.331129 -2.714055 -0.002577

Nb 2.257413 -1.842029 0.034593

O 3.524651 -0.287879 0.065793

O 1.871641 -1.486075 1.989783

O 0.718384 0.124791 4.252498

O -0.510348 1.653464 1.942462

O 1.961292 -1.495612 -1.935204

O 3.364248 -3.251217 0.064814

O -1.840138 3.470797 -0.049504

O -0.433278 1.641669 -2.008672

O 0.922483 0.118616 -4.253991

Cs 1.260348 3.947731 -2.852216

Cs 0.234224 -3.901168 -2.793839

Cs 4.661078 -0.462745 -2.739150

Cs 1.135759 3.970976 2.837826

Cs 0.130973 -3.878341 2.795523

Cs -3.534627 0.631324 2.476329

Cs 4.521264 -0.454414 2.937915

Cs -3.479446 0.492782 -2.630763

H -3.471643 2.855070 -0.113939

H -3.626824 -2.042210 0.114660

H -6.272792 3.003275 -0.479190

H -6.218283 4.933191 1.162966

H -5.787859 3.383575 1.901768

H -4.513217 4.555332 1.485141

H -5.699555 5.316494 -1.331825

H -3.979918 4.955965 -1.039996

H -4.886870 4.041385 -2.255216

H -7.639946 -2.695082 -0.277070

H -7.799049 -2.011408 1.351958

H -8.636935 -1.250056 -0.025424

O -2.248996 -4.858084 -1.255489

O -2.336377 -4.781064 1.219737

S -3.029931 -4.450677 -0.055469

57

Cs8PONb-MPFA-iPOH/NO2(Ot) -2959.85606948 -2959.544662 -2959.723215 0

C 7.967079 0.601217 1.148874

P 6.482054 -0.100491 0.383768

O 6.474766 0.151369 -1.096001

O 4.292722 -2.046450 -0.770785

C 4.577804 -3.220630 -1.513947

C 4.063611 -3.095826 -2.940000

F 6.819733 -1.690350 0.669361

O 5.276932 0.206277 1.257785

C 4.040091 -4.461373 -0.815063

O 3.102627 1.548476 0.451630

Nb 1.269227 0.918429 0.231991

O 0.942470 1.515536 -1.651385

Nb -0.871534 0.839948 -2.291893

O -1.708593 2.313381 -1.149221

Nb -1.814093 1.984667 0.843586

O -1.430637 0.918871 2.521087

Nb -0.606856 -0.952370 2.257822

O -0.543622 -1.600546 3.930602

O 0.231738 2.456677 0.859076

O 1.877110 -0.916613 -0.526176

Nb 0.286558 -2.110724 -0.858459

O 0.918516 -3.660310 -1.501875

O -0.668335 -0.015825 -0.002006

Nb -2.949610 -1.074044 -0.277328

O -2.423455 -1.575530 1.628419

O 1.148846 0.065612 2.037373

O -2.559541 3.489187 1.477825

O -3.380891 0.790212 0.479883

O -0.019106 -0.978824 -2.533784

O -1.707988 -2.531132 -0.896844

O 0.231833 -2.418875 1.149068

O -1.103957 1.518127 -3.932847

O -2.630767 -0.180279 -2.060553

O -4.578562 -1.799768 -0.455375

Cs -4.225285 0.115829 3.314146

Cs 0.298633 4.517451 -1.363009

Cs 3.802605 0.690167 -2.610063

Cs -4.551912 2.095266 -2.043347

Cs -2.085403 -2.590345 -3.910367

Cs -1.836882 -4.536074 1.336409

Cs 0.899281 2.553034 3.751570

Cs 3.287191 -2.079946 2.172072

H 3.336322 -1.804503 -0.823233

H 3.941285 1.106013 0.743149

H 5.675672 -3.264375 -1.531536

H 4.299013 -5.372801 -1.366682

H 4.478499 -4.550754 0.186657

H 2.945700 -4.411620 -0.745147

H 4.213147 -4.019720 -3.509379

H 2.986596 -2.881289 -2.935911

H 4.606386 -2.295610 -3.456604

H 7.930510 1.689730 1.064859

H 8.020503 0.333915 2.206674

H 8.864919 0.243327 0.640226

N 3.672449 4.129219 0.020871

O 3.532427 4.124408 -1.202260

O 2.885531 4.646669 0.808717

42

Cs8PONb-MPFA -2560.40506017 -2560.221784 -2560.377659 0

H -4.033665 -1.874979 0.609765

O -3.152612 -2.279157 0.470779

Nb -1.518393 -1.204164 0.218955

O -1.340083 -0.548212 2.116527

O -0.221916 -2.667333 0.641158

O -1.184031 -1.533633 -1.724074

O -2.299041 0.550266 -0.253082

Nb 0.520581 -0.595739 -2.364525

O 0.657601 -1.063415 -4.095978

O 1.534718 -2.113985 -1.447344

Nb 1.728339 -1.983113 0.568867

O 2.684198 -3.453696 0.976548

O 1.379658 -1.142814 2.383751

Nb 0.314904 0.610403 2.365480

O 0.283790 1.058470 4.107342

O -0.722271 2.104720 1.464905

Nb -0.855670 1.989911 -0.556731

O -1.782575 3.466414 -0.980359

O -0.556575 1.114583 -2.378293

O 0.366108 -0.041977 0.012979

Nb 2.482218 1.270257 -0.235362

O 2.010791 1.522198 1.720809

O 3.157762 -0.584575 0.268777

O 2.180102 0.548365 -2.109167

O 1.051100 2.659486 -0.646248

O 4.007575 2.205176 -0.407060

Cs 1.149421 4.425358 1.786536

Cs 4.113744 -0.121270 3.082505

Cs 4.320091 -1.534728 -2.344700

Cs 1.362092 3.034678 -3.599733

Cs -3.677671 1.483245 2.217316

Cs -3.487321 0.217412 -2.974535

Cs -0.394302 -4.414358 -1.809433

Cs -0.649492 -3.031187 3.578035

O -5.538057 -0.850662 1.027532

P -6.392254 -0.186135 -0.026597

F -6.349377 1.434918 0.373855

O -6.087019 -0.277207 -1.493566

C -8.149492 -0.473961 0.294115

H -8.393220 -0.259442 1.336621

H -8.381475 -1.521806 0.091081

H -8.762391 0.150668 -0.358906

45

Cs8PONb-MPFA/CO2(Ot) -2749.01539595 -2748.813323 -2748.976209 0

F -6.576017 -1.383570 -1.336598

P -6.584586 -0.780684 0.211523

C -8.198580 0.028748 0.160704

O -5.456764 0.231498 0.218499

O -6.546900 -1.934701 1.166839

O -2.146982 -0.600612 -0.073400

Nb -0.769435 -2.048658 -0.519058

O 1.124938 -2.683842 -0.771288

Nb 2.604683 -1.326055 -0.432246

O 4.106668 -2.251380 -0.746136

Nb -1.229051 1.078317 0.410876

O 0.481930 0.013733 0.040032

Nb 0.633443 -0.743694 2.381740

O 2.241224 -1.632686 1.546882

O -2.968944 2.223607 0.817044

O -1.175493 1.554097 -1.536933

Nb 0.464434 0.631960 -2.354302

O 2.134900 -0.535037 -2.226780

O -1.063612 0.460469 2.290112

O 0.023424 2.606163 0.781217

Nb 1.939859 1.931665 0.535303

O 3.305035 0.502644 0.097400

O 1.581637 2.086329 -1.452830

O 0.507050 1.110325 -4.080086

O -0.608494 -1.079973 -2.310147

O 1.688408 1.002800 2.339211

O 2.976509 3.347175 0.916169

O -1.703740 -3.524251 -0.904222

O -0.493994 -2.170709 1.492572

O 0.773653 -1.255473 4.092421

Cs 1.336466 -4.543627 1.605956

Cs -0.247056 2.950232 3.717717

Cs 4.457513 -0.030828 2.824007

Cs 1.163213 -2.949196 -3.803184

Cs -0.375385 4.479911 -1.597246

Cs -3.717016 -0.137372 -2.522722

Cs 4.284635 1.537223 -2.592666

Cs -3.583002 -1.545639 2.322513

H -5.013514 1.853336 -0.047518

H -8.389833 0.507874 1.123183

H -8.222107 0.792719 -0.619394

H -8.984550 -0.705830 -0.024816

C -3.561200 3.114688 0.070459

O -4.774941 2.752650 -0.390950

O -3.111105 4.220921 -0.226543

45

Cs8PONb-MPFA/SO2(Ot) -3109.02638010 -3108.829592 -3108.994081 0

F -5.875631 2.671373 0.774747

P -6.204426 1.289747 -0.092349

C -7.977180 1.199099 0.242216

O -5.460089 0.205106 0.659864

O -5.930904 1.591543 -1.535727

O -2.039792 1.108033 -0.112320

Nb -0.405931 2.314841 -0.107439

O 1.614718 2.646412 -0.047968

Nb 2.805408 1.030288 0.035064

O 4.463280 1.710297 0.073850

Nb -1.427097 -0.810157 -0.035601

O 0.462339 0.013980 -0.003524

Nb 0.633501 -0.016200 -2.465240

O 2.430551 0.769202 -1.934327

O -3.471645 -1.455147 -0.144664

O -1.342854 -0.667081 1.953646

Nb 0.480291 0.137452 2.459103

O 2.307031 0.880708 1.993822

O -1.206492 -0.791822 -2.027398

O -0.538274 -2.549724 0.047975

Nb 1.552109 -2.193990 0.100904

O 3.154969 -1.002052 0.107734

O 1.253478 -1.708598 2.041361

O 0.503741 0.204471 4.250552

O -0.283409 1.919586 1.888766

O 1.369859 -1.830772 -1.882652

O 2.263089 -3.838333 0.173114

O -1.003876 3.999793 -0.173543

O -0.166843 1.799978 -2.058413

O 0.769105 -0.064894 -4.252179

Cs 1.993952 3.664239 -2.870578

Cs -0.878166 -3.741407 -2.699748

Cs 4.249655 -1.496131 -2.670465

Cs 1.847488 3.807854 2.720080

Cs -1.021175 -3.588070 2.854093

Cs -3.344636 1.623358 2.543553

Cs 4.117068 -1.291730 2.959467

Cs -3.215957 1.355089 -2.873826

H -4.198103 -0.830431 0.120108

H -8.390455 0.318112 -0.253276

H -8.167010 1.118321 1.314369

H -8.477352 2.086863 -0.150461

O -3.498764 -3.950635 -1.100623

O -3.716759 -3.707999 1.357458

S -4.233671 -3.300632 0.020611

45

Cs8PONb-MPFA/NO2(Ot) -2765.49333037 -2765.296679 -2765.464972 0

C 8.097712 -1.186489 0.242329

P 6.348612 -1.341543 -0.177777

O 6.072683 -0.954702 -1.601778

F 6.229114 -3.005161 -0.107567

O 5.471516 -0.863613 0.959003

O 3.444950 0.942357 0.334336

Nb 1.527583 0.552542 0.160972

O 1.159916 1.537758 -1.548657

Nb -0.788637 1.290195 -2.080639

O -1.270496 2.614838 -0.622885

Nb -1.290558 1.909279 1.285699

O -0.933460 0.495117 2.694598

Nb -0.431452 -1.378367 2.030716

O -0.377678 -2.339227 3.542634

O 0.774644 2.072914 1.148391

O 1.804438 -1.140605 -0.961920

Nb 0.043249 -2.046028 -1.320228

O 0.392690 -3.533308 -2.251010

O -0.534224 -0.035296 -0.021542

Nb -2.951582 -0.701426 -0.237208

O -2.364088 -1.621049 1.467080

O 1.448346 -0.622233 1.785387

O -1.787120 3.340257 2.244908

O -3.043171 1.027349 0.872219

O -0.218944 -0.568080 -2.738344

O -1.996415 -2.149343 -1.258732

O 0.057004 -2.716115 0.586464

O -1.032499 2.261437 -3.564403

O -2.642600 0.487586 -1.851769

O -4.678769 -1.159105 -0.389618

Cs -3.728375 -0.054827 3.650948

Cs 1.131017 4.500373 -0.662269

Cs 3.649485 0.342862 -2.902422

Cs -4.193108 2.959028 -1.157537

Cs -2.639905 -1.538179 -4.134856

Cs -2.281852 -4.558922 0.555457

Cs 1.929223 1.555431 3.822249

Cs 3.172520 -3.062402 0.962246

H 4.206531 0.374268 0.577458

H 8.364264 -0.127765 0.265619

H 8.294764 -1.617170 1.225942

H 8.715155 -1.686811 -0.506193

N 4.467134 3.631424 0.523524

O 4.279883 3.791694 -0.685728

O 3.682631 4.052248 1.384055

52

Cs8PONb-iMPA -2654.30103459 -2654.016729 -2654.180483 0

C 7.779282 -1.673803 0.376769

P 6.120209 -1.105777 -0.102903

O 5.101039 -1.947612 0.640436

O 6.052894 -0.891557 -1.597834

O 6.045887 0.396933 0.657910

C 6.253281 1.600825 -0.093197

C 7.732166 1.816682 -0.357301

C 5.619638 2.731843 0.689793

O 2.528098 -2.903385 -0.033617

Nb 1.063812 -1.588349 -0.023961

O 2.192874 0.050824 -0.098975

Nb 1.097364 1.765620 -0.100595

O 2.295043 3.104148 -0.166193

O -0.501240 -2.810463 0.060906

Nb -2.291527 -1.731421 0.100053

O -3.518933 -3.047077 0.170130

Nb -0.500616 0.073419 2.434419

O 0.867010 1.468206 1.899313

O 0.905675 -1.334344 1.961425

O -0.541286 -0.044363 -0.001570

Nb -0.664174 -0.035344 -2.436883

O -2.025397 -1.472048 -1.891049

O 0.773961 -1.429239 -2.003666

O -3.397962 -0.048699 0.099984

Nb -2.339661 1.702054 0.020811

O -3.646853 2.934528 0.034302

O -1.892458 -1.384005 2.058301

O -1.946405 1.432690 1.994895

O -0.646058 2.808500 -0.066222

O -2.074089 1.346272 -1.960222

O -0.485942 0.109756 4.232688

O 0.732976 1.380008 -2.071805

O -0.787655 -0.088527 -4.230337

Cs -0.676805 3.863186 -2.892486

Cs -0.471788 3.986565 2.686816

Cs -0.399856 -3.841744 2.900895

Cs -4.567878 -0.121675 -2.676811

Cs -0.616948 -3.962592 -2.721401

Cs -4.390307 0.009719 2.952315

Cs 3.555121 0.027101 2.589226

Cs 3.393663 -0.089358 -2.861697

H 8.559156 -1.049190 -0.066580

H 3.464715 -2.653406 0.122722

H 7.918835 -2.700552 0.028719

H 7.886169 -1.659974 1.464491

H 5.739036 1.497440 -1.060490

H 5.816505 3.692914 0.203613

H 4.530205 2.628369 0.740897

H 6.043268 2.775865 1.701737

H 7.901629 2.764548 -0.879997

H 8.289870 1.840823 0.586273

H 8.132635 1.010842 -0.979357

55

PONb-iMPA/CO2(Ot) -2842.91171327 -2842.609097 -2842.778721 0

P -6.255509 0.241708 0.503965

C -7.796073 0.636868 -0.373918

O -5.277491 1.396570 0.389803

O -6.559683 -0.372874 1.847239

O -5.560126 -0.897650 -0.507106

C -6.033483 -2.254949 -0.531829

C -5.452332 -3.057572 0.617174

C -5.609351 -2.822215 -1.872641

O -2.099079 -0.037140 0.098716

Nb -1.095129 -1.755777 -0.326913

O 0.615427 -2.789182 -0.597800

Nb 2.370115 -1.776031 -0.436909

O 3.617767 -3.020305 -0.761116

Nb -0.821639 1.439393 0.405295

O 0.601727 0.015247 0.028383

Nb 0.740859 -0.619479 2.402419

O 2.081689 -1.887187 1.572756

O -2.320564 2.894783 0.765276

O -0.777260 1.778928 -1.568209

Nb 0.579406 0.475191 -2.396334

O 1.955479 -1.014697 -2.265378

O -0.658620 0.924315 2.315109

O 0.746531 2.683219 0.605423

Nb 2.461059 1.604585 0.313829

O 3.470534 -0.109171 -0.089979

O 2.035050 1.715197 -1.661912

O 0.598460 0.827875 -4.151858

O -0.840440 -0.949474 -2.175797

O 2.134400 0.849713 2.183287

O 3.787335 2.786580 0.571182

O -2.317231 -3.040966 -0.598655

O -0.720841 -1.814283 1.679071

O 0.876902 -1.036491 4.139639

Cs 0.568579 -4.489546 1.917647

Cs 0.813869 3.229009 3.535542

Cs 4.640183 -0.707112 2.631604

Cs 0.395971 -3.272610 -3.573417

Cs 0.564095 4.443194 -1.889220

Cs -3.601623 0.737134 -2.350075

Cs 4.476972 0.508752 -2.883475

Cs -3.466720 -0.416213 2.714894

H -4.474564 2.855513 0.056294

H -7.134843 -2.245933 -0.456993

H -5.971009 -3.848627 -1.994638

H -6.007777 -2.221358 -2.698510

H -4.513342 -2.843745 -1.922782

H -5.794567 -4.097718 0.561336

H -4.357753 -3.058762 0.537222

H -5.779351 -2.635537 1.570792

H -8.350651 1.390278 0.190048

H -7.587039 1.033325 -1.371726

H -8.429412 -0.250213 -0.466371

C -2.791240 3.807752 -0.041083

O -4.085402 3.657900 -0.379182

O -2.156641 4.759258 -0.496359

55

PONb-iMPA/SO2(Ot) -3202.92023274 -3202.622910 -3202.794065 0

P 6.055726 -0.002274 -0.150781

C 7.767860 0.140070 0.438500

O 5.203481 1.031390 0.568038

O 6.042464 -0.140644 -1.653113

O 5.566097 -1.435639 0.567785

C 5.991248 -2.714573 0.080070

C 5.183024 -3.145333 -1.130062

C 5.815700 -3.683431 1.233868

O 2.112443 -0.521953 -0.085192

Nb 0.810568 -2.073408 -0.086820

O -1.082449 -2.851197 -0.062623

Nb -2.626310 -1.561999 0.017970

O -4.074613 -2.617853 0.031779

Nb 1.069806 1.198917 -0.020840

O -0.576060 -0.034282 -0.002401

Nb -0.733257 -0.054362 -2.469525

O -2.307612 -1.216423 -1.955177

O 2.916611 2.283057 -0.096682

O 0.982145 1.034091 1.969983

Nb -0.599768 -0.164412 2.462405

O -2.204516 -1.310071 1.982988

O 0.863353 1.136447 -2.006034

O -0.199254 2.694381 0.060393

Nb -2.141122 1.862167 0.079767

O -3.430722 0.326935 0.079112

O -1.767114 1.454648 2.027227

O -0.616624 -0.225088 4.254632

O 0.561767 -1.722925 1.906368

O -1.867041 1.548547 -1.901902

O -3.223891 3.290104 0.140787

O 1.801218 -3.567203 -0.135265

O 0.467697 -1.628394 -2.044776

O -0.843080 -0.047663 -4.258980

Cs -1.254082 -3.913478 -2.892862

Cs -0.091912 3.961988 -2.666468

Cs -4.601606 0.579133 -2.690776

Cs -1.114608 -4.055168 2.708371

Cs 0.036765 3.804558 2.863374

Cs 3.437602 -0.615149 2.656047

Cs -4.444376 0.454862 2.941328

Cs 3.285752 -0.418238 -2.917284

H 3.777021 1.815970 0.104887

H 7.059846 -2.656751 -0.190960

H 6.181043 -4.680669 0.969171

H 6.358786 -3.341224 2.120848

H 4.750416 -3.775715 1.478020

H 5.439094 -4.173828 -1.409383

H 4.110187 -3.118201 -0.895418

H 5.408352 -2.492479 -1.976214

H 8.206409 1.060278 0.045830

H 7.801697 0.176452 1.530269

H 8.374040 -0.699911 0.086887

O 2.473990 4.706892 -1.140289

O 2.584525 4.560727 1.331699

S 3.260425 4.247238 0.040016

55

PONb-iMPA/NO2(Ot) -2859.38986061 -2859.091347 -2859.263505 0

P -6.099455 -0.181192 -0.580655

C -7.827808 -0.554111 -0.160501

O -5.242693 -1.397111 -0.282593

O -6.043407 0.528908 -1.912669

O -5.673379 0.878343 0.651416

C -6.140047 2.232369 0.674708

C -5.328608 3.117994 -0.253011

C -6.027719 2.690889 2.115984

O -2.127865 0.391620 -0.075720

Nb -0.916087 1.878246 0.547013

O 0.921540 2.683810 0.954827

Nb 2.521474 1.500340 0.639408

O 3.915326 2.534042 1.097577

Nb -1.084177 -1.245867 -0.589455

O 0.579330 0.022011 -0.025668

Nb 0.798360 0.908261 -2.294656

O 2.289237 1.885699 -1.340510

O -2.751530 -2.257336 -1.099277

O -0.995944 -1.756370 1.337392

Nb 0.489563 -0.735077 2.305926

O 2.051761 0.559302 2.372248

O -0.750001 -0.438157 -2.388265

O 0.325039 -2.584972 -0.945953

Nb 2.209419 -1.724024 -0.532050

O 3.432037 -0.246367 0.060651

O 1.773887 -2.055363 1.418637

O 0.424462 -1.348120 3.991663

O -0.747081 0.867848 2.302953

O 2.005008 -0.758559 -2.297436

O 3.344292 -3.062703 -0.923170

O -1.983587 3.265193 0.949290

O -0.495680 2.186945 -1.435902

O 0.975781 1.501707 -3.981460

Cs 1.074392 4.704687 -1.249795

Cs 0.438907 -2.707366 -3.946789

Cs 4.664179 0.583346 -2.590268

Cs 0.755907 2.811735 3.963654

Cs 0.134536 -4.564579 1.392136

Cs -3.603161 -0.664342 2.369463

Cs 4.382354 -1.310044 2.709508

Cs -3.221525 1.306879 -2.770227

H -3.662121 -1.930846 -0.897043

H -7.199958 2.249776 0.365486

H -6.421667 3.704728 2.239121

H -6.579971 2.022214 2.784468

H -4.972673 2.705907 2.414802

H -5.636199 4.164431 -0.142654

H -4.260984 3.052975 -0.000499

H -5.501478 2.814440 -1.288053

H -8.225734 -1.267528 -0.885849

H -7.897794 -0.995522 0.836950

H -8.445783 0.347617 -0.200325

O -2.523562 -5.119150 -0.535961

O -3.272200 -4.017414 1.199848

N -3.196139 -4.265432 0.012687

39

Cs8PONb_2co2(Ot) -2305.33710888 -2305.188697 -2305.339574 0

Nb -0.555289 -0.352928 2.396454

O -0.883861 -0.633439 4.131377

O 0.006617 0.136590 0.033390

O -0.414769 1.679915 2.272146

O -2.329648 -0.197472 1.506008

O -0.366278 -2.188690 1.560442

O 1.541026 -0.318279 2.349642

Nb 0.565099 0.562078 -2.348365

O -1.522266 0.602889 -2.294988

O 0.454034 2.404759 -1.455445

O 2.335501 0.363543 -1.451919

O 0.371305 -1.442426 -2.269099

O 0.924666 0.963666 -4.052883

Nb 2.202015 0.039540 0.540590

Nb 0.027302 2.563570 0.510010

Nb -2.162077 0.260984 -0.470992

Nb 0.000258 -2.294418 -0.440336

O -1.911944 -1.698479 -0.705336

O 1.922006 -1.860755 0.021252

O 1.967738 1.988749 0.852220

O -1.933966 2.174221 -0.014363

O 4.149132 -0.069699 0.848057

O -0.007275 -4.048567 -0.789138

O -4.116305 0.295234 -0.798393

O 0.036162 4.319005 0.842064

Cs -3.498127 -3.018667 1.492276

Cs -2.622982 -2.047975 -3.503485

Cs -1.920733 3.564458 -2.760434

Cs 3.482532 -2.264341 -2.512114

Cs 4.057088 2.702417 -1.350239

Cs -4.171829 1.973003 2.167179

Cs 2.598591 -3.217687 2.502584

Cs 1.971522 2.201789 3.935773

C -4.983290 -0.746897 -0.414764

O -5.055898 -1.710614 -1.209885

O -5.535272 -0.587054 0.702724

C 5.020299 -0.832875 0.039502

O 5.114234 -2.041754 0.348751

O 5.545672 -0.202350 -0.909755

42

Cs8PONb_3co2(Ot) -2493.94863586 -2493.783729 -2493.943970 0

Nb 0.739457 -0.476681 -2.416096

O 1.305491 -0.676162 -4.087892

O 0.073365 -0.034442 -0.068936

O 0.043816 1.509011 -2.309144

O 2.368967 0.271059 -1.452732

O 1.154527 -2.225431 -1.537553

O -1.216987 -0.988388 -2.370418

Nb -0.637355 0.104706 2.305861

O 1.318298 0.827435 2.252105

O -1.063110 1.874453 1.361368

O -2.281452 -0.565286 1.357941

O 0.127908 -1.731305 2.234171

O -1.174557 0.397446 3.979755

Nb -2.122713 -0.820550 -0.611996

Nb -0.477488 2.071833 -0.517722

Nb 2.160553 0.564413 0.506245

Nb 0.703738 -2.479355 0.420930

O 2.417797 -1.323379 0.757659

O -1.193534 -2.565012 -0.080631

O -2.325991 1.068001 -0.888558

O 1.350443 2.457608 0.002677

O -3.841958 -1.623866 -1.015740

O 1.191454 -4.152085 0.774051

O 4.019104 1.127214 0.874252

O -1.279044 3.850496 -0.826693

Cs 4.333578 -2.143330 -1.521633

Cs 3.376016 -1.238825 3.534366

Cs 0.355836 4.068994 2.636074

Cs -2.679937 -3.415249 2.446339

Cs -4.168221 1.934134 1.463934

Cs 3.595321 2.817380 -2.196958

Cs -1.711588 -4.014800 -2.604268

Cs -2.738842 2.447820 -3.473728

C 5.139709 0.410988 0.397639

O 5.513689 -0.534281 1.127337

O 5.576784 0.784987 -0.715877

C -4.453373 -2.643164 -0.207493

O -4.157332 -3.809841 -0.549414

O -5.141255 -2.219205 0.735811

C -2.461846 4.301580 -0.191815

O -3.505704 4.149722 -0.868372

O -2.315691 4.750985 0.965700

45

Cs8PONb_4co2(Ot) -2682.55012186 -2682.368589 -2682.534149 0

Nb -1.023448 -0.196384 2.050023

O -2.095553 -0.122430 3.642864

O 0.071755 -0.017466 -0.013356

O -0.239239 1.720387 1.990161

O -2.400138 0.530374 0.804071

O -1.383607 -1.990865 1.362040

O 0.796881 -0.757887 2.525251

Nb 1.230847 -0.082347 -2.270975

O -0.662550 0.654137 -2.646862

O 1.564609 1.719837 -1.422375

O 2.634298 -0.754658 -0.955746

O 0.367806 -1.897300 -2.189849

O 2.074700 -0.011120 -3.830300

Nb 2.091267 -0.820078 0.938018

Nb 0.634926 2.103574 0.308794

Nb -1.890049 0.667911 -1.125828

Nb -0.589963 -2.430615 -0.499805

O -2.128693 -1.252799 -1.208091

O 1.154641 -2.549305 0.422043

O 2.319704 1.078416 1.122960

O -1.032130 2.474058 -0.609661

O 3.625464 -1.639089 1.760756

O -1.086266 -4.108466 -0.799219

O -3.591816 1.186353 -1.886899

O 1.447631 3.865922 0.598690

Cs -4.508649 -1.863349 0.809263

Cs -2.253589 -1.601840 -4.178249

Cs 0.541245 4.058738 -2.904763

Cs 3.006391 -3.733686 -1.731394

Cs 4.653043 1.703335 -0.808922

Cs -4.451569 2.622829 1.355130

Cs 0.844505 -3.824770 3.094479

Cs 2.130491 2.654526 3.695641

C -4.797998 0.393236 -1.878709

O -4.871857 -0.438073 -2.797709

O -5.559943 0.651344 -0.925854

C 4.210099 -2.897614 1.335761

O 3.743589 -3.894629 1.917217

O 5.049005 -2.779201 0.429270

C 2.788309 4.217591 0.282681

O 3.600223 4.066893 1.220786

O 2.975739 4.580666 -0.898237

C -3.346133 0.602445 3.728064

O -3.246202 1.757435 4.161852

O -4.331193 -0.033362 3.297294

48

Cs8PONb_5co2(Ot) -2871.14201361 -2870.943980 -2871.116286 0

Nb 1.572071 -0.987626 -1.549363

O 2.893879 -1.496743 -2.829872

O 0.018979 -0.088833 0.033886

O 0.496307 0.409692 -2.576542

O 2.510581 0.575274 -0.750161

O 2.080074 -2.038726 0.039228

O 0.024866 -2.174357 -1.796116

Nb -1.510131 0.632878 1.746976

O 0.054555 1.924198 1.901797

O -1.967249 1.666948 0.107637

O -2.506282 -0.888226 0.950608

O -0.438764 -0.643482 2.766195

O -2.782682 1.374778 2.958381

Nb -1.552424 -1.808626 -0.560336

Nb -0.818830 1.354826 -1.501180

Nb 1.550558 1.548506 0.713842

Nb 1.026712 -1.670084 1.739212

O 2.093921 0.048362 1.870602

O -0.471925 -2.694685 0.874547

O -2.040396 -0.347092 -1.718307

O 0.545549 2.522826 -0.760113

O -2.718120 -3.257418 -0.964571

O 1.697364 -2.785732 2.938929

O 2.914257 2.792106 1.272410

O -1.828758 2.368971 -2.802733

Cs 4.999794 -0.725876 0.635957

Cs 1.816614 1.584563 4.458690

Cs -2.534573 4.618691 0.259244

Cs -2.506086 -3.063364 3.200251

Cs -4.893190 0.676427 -0.561218

Cs 4.006345 2.580351 -2.420395

Cs 0.899534 -4.976898 -0.703790

Cs -1.536245 -0.586815 -4.696016

C 4.287315 2.449763 1.563031

O 4.486951 2.112915 2.741676

O 5.041293 2.503736 0.573658

C -2.859635 -4.508057 -0.193033

O -2.128883 -5.418469 -0.601673

O -3.666093 -4.412313 0.739742

C -3.262205 2.383446 -2.958724

O -3.699359 1.519633 -3.738210

O -3.841549 3.230745 -2.254191

C 3.982688 -0.614048 -3.252351

O 3.743565 0.024096 -4.282310

O 4.949206 -0.607500 -2.467502

C -3.528284 2.612054 2.699719

O -3.019522 3.613598 3.213377

O -4.513790 2.454058 1.958781

51

Cs8PONb_6co2(Ot) -3059.72578979 -3059.512077 -3059.694508 0

Nb -1.375738 -0.548846 -1.979688

O -2.532943 -1.035316 -3.397270

O -0.000460 -0.042853 -0.064459

O 0.021540 -2.014393 -1.964453

O -2.230926 -1.474021 -0.498800

O -2.211234 1.169561 -1.310060

O 0.058959 0.589652 -2.759285

Nb 1.376669 0.597142 1.816247

O -0.080335 -0.629099 2.622361

O 2.147065 -1.092217 1.129806

O 2.266911 1.551530 0.348670

O -0.026121 1.976536 1.820794

O 2.539771 0.911949 3.281255

Nb 1.448362 1.287274 -1.482024

Nb 1.266583 -1.933815 -0.463837

Nb -1.366037 -1.291394 1.364417

Nb -1.378388 1.912591 0.319239

O -2.283411 0.471445 1.349892

O 0.073863 2.598677 -0.870743

O 2.281201 -0.473243 -1.513407

O -0.081213 -2.701419 0.729102

O 2.493723 2.414923 -2.578201

O -2.360287 3.454166 0.745924

O -2.679829 -2.240003 2.385999

O 2.556818 -3.305588 -0.854446

Cs -5.063813 0.048020 -0.123477

Cs -2.320707 1.069262 4.336562

Cs 3.199641 -3.251633 2.951562

Cs 1.540277 4.698495 0.962481

Cs 5.044599 -0.070131 0.071658

Cs -3.167571 -4.326087 -0.634421

Cs -1.350509 3.307784 -3.560431

Cs 2.206840 -1.956686 -4.169140

C -4.115431 -1.986494 2.361340

O -4.477260 -1.070715 3.111200

O -4.726841 -2.701267 1.548163

C 2.312203 3.911404 -2.687790

O 1.554184 4.220115 -3.609797

O 2.933100 4.521896 -1.819146

C 3.998755 -3.143898 -0.946275

O 4.399584 -2.830526 -2.077611

O 4.589205 -3.303430 0.135317

C -3.645485 -2.017040 -3.219518

O -3.317854 -3.167603 -3.516701

O -4.671958 -1.500932 -2.763522

C -2.174121 4.287062 2.032772

O -2.809460 3.809701 2.967180

O -1.414884 5.230859 1.845307

C 3.499752 -0.083938 3.797266

O 4.532961 -0.170587 3.112880

O 3.085483 -0.704031 4.777939

39

Cs8PONb_2so2(Ot) -3025.37922759 -3025.239730 -3025.390822 0

O -0.982314 -0.845372 -4.054187

O -0.005205 -0.107943 0.018613

O -0.463801 -2.358618 -1.531506

O -2.352018 -0.322339 -1.405983

O -0.433330 1.522692 -2.234882

O 1.480930 -0.500768 -2.341555

Nb 0.557306 0.329671 2.372377

O -1.480841 0.300037 2.375522

O 0.482620 -1.707551 2.232531

O 2.349636 0.184343 1.419844

O 0.412543 2.187201 1.581627

O 0.992073 0.585815 4.089246

Nb 2.227861 -0.193095 -0.509545

Nb 0.003093 -2.528854 0.431772

Nb -2.219962 -0.024640 0.537953

Nb -0.004134 2.325924 -0.401935

O -1.925858 1.883842 0.104382

O 1.910550 1.756725 -0.737093

O 1.937720 -2.121594 -0.113902

O -1.920888 -1.983284 0.821521

O 4.125325 -0.333609 -1.013511

O -0.010714 4.084760 -0.720114

O -4.119613 -0.016242 1.069340

O 0.022060 -4.293590 0.734573

Cs -3.561436 2.256116 -2.433834

Cs -2.447735 3.201765 2.667737

Cs -1.996512 -2.277207 3.821372

Cs 3.563080 2.924695 1.569964

Cs 3.714338 -2.209813 2.399456

Cs -3.738454 -2.860786 -1.577093

Cs 2.452266 2.180313 -3.561642

Cs 1.994702 -3.413951 -2.854653

O -5.256192 2.094538 0.271548

O -5.518379 -0.011180 -1.037844

O 5.330114 1.885376 -0.892565

O 5.489848 0.300773 1.025528

S -5.563879 0.614398 0.344079

S 5.583811 0.451579 -0.481525

42

Cs8PONb_3so2(Ot) -3574.01093109 -3573.858020 -3574.022341 0

Nb 0.646685 -0.475183 -2.423158

O 1.159411 -0.710480 -4.109659

O 0.070003 -0.042817 -0.054242

O 0.015900 1.488732 -2.309982

O 2.326560 0.213289 -1.492138

O 1.068323 -2.252085 -1.554708

O -1.305785 -0.970998 -2.327298

Nb -0.603530 0.125033 2.304565

O 1.380420 0.767294 2.224285

O -0.984538 1.929494 1.386804

O -2.289892 -0.507106 1.424605

O 0.120305 -1.732071 2.238939

O -1.049212 0.452158 4.001862

Nb -2.201813 -0.799264 -0.556822

Nb -0.457987 2.088781 -0.501086

Nb 2.208780 0.515738 0.463150

Nb 0.653059 -2.461167 0.400170

O 2.423654 -1.369852 0.726469

O -1.268305 -2.520443 -0.060235

O -2.311910 1.108908 -0.812012

O 1.389808 2.413697 -0.025098

O -3.965426 -1.447638 -0.967726

O 1.087782 -4.151616 0.756302

O 4.021174 1.229992 0.795807

O -1.116326 3.892770 -0.978698

Cs 4.248716 -2.227407 -1.591622

Cs 3.030360 -1.526844 3.598713

Cs 0.672314 3.910907 2.712518

Cs -2.598159 -3.472647 2.451726

Cs -4.008002 2.171464 1.574490

Cs 3.586253 2.721525 -2.294050

Cs -1.439926 -4.028509 -2.628996

Cs -2.779725 2.423050 -3.411122

O 5.486630 -0.820508 1.074412

O 5.702465 0.594153 -0.966313

O -3.971749 -4.016614 -0.805403

O -5.165939 -2.704390 0.927242

O -3.576831 4.321053 -0.812855

O -2.132732 4.820796 1.160756

S 5.617012 0.592334 0.550582

S -4.951503 -2.894770 -0.547044

S -2.297629 4.985441 -0.336645

45

Cs8PONb_4so2(Ot) -4122.63534550 -4122.469615 -4122.641843 0

Nb 0.929145 -0.317531 -2.045288

O 1.849730 -0.428646 -3.705252

O -0.109433 -0.058956 0.078622

O 0.279846 1.604316 -1.989543

O 2.374304 0.304917 -0.816928

O 1.203751 -2.130785 -1.303968

O -0.942252 -0.808680 -2.444407

Nb -1.220061 -0.029323 2.313175

O 0.719859 0.604485 2.661975

O -1.442599 1.825551 1.448946

O -2.699204 -0.596160 1.056219

O -0.481549 -1.883150 2.274842

O -2.015838 0.174426 3.889368

Nb -2.210328 -0.757808 -0.862160

Nb -0.563105 2.108290 -0.285704

Nb 1.917543 0.522356 1.112216

Nb 0.424727 -2.476557 0.553142

O 2.044627 -1.406978 1.239711

O -1.356412 -2.499826 -0.323007

O -2.288732 1.172758 -1.052114

O 1.167220 2.362517 0.570187

O -3.818287 -1.396408 -1.676574

O 0.799037 -4.186734 0.862711

O 3.591290 1.098506 1.905188

O -1.076325 3.949615 -0.712838

Cs 4.385406 -2.076999 -0.762112

Cs 2.246970 -1.783440 4.154476

Cs -0.040549 3.780634 3.131714

Cs -3.185578 -3.604173 1.813581

Cs -4.446682 2.082197 0.988961

Cs 4.154950 2.545418 -1.499012

Cs -0.989004 -3.902420 -2.912407

Cs -2.111905 2.725329 -3.616045

O 4.957670 -0.997140 2.403987

O 5.670908 0.529863 0.570661

O -3.848862 -3.983093 -1.621064

O -5.440506 -2.740571 -0.186086

O -3.515235 4.391318 -1.086053

O -2.561314 4.750621 1.192034

O 3.265634 1.629380 -4.420664

O 4.417087 -0.247741 -3.296609

S 3.463936 0.145953 -4.396126

S 5.236161 0.434429 2.012751

S -2.392502 5.020055 -0.287195

S -4.876258 -2.878798 -1.567492

48

Cs8PONb_5so2(Ot) -4671.24810314 -4671.069976 -4671.253674 0

Nb -0.913031 -0.115630 -2.033353

O -1.837897 -0.219304 -3.667672

O 0.195039 -0.059554 0.090964

O 0.320986 -1.698809 -2.058159

O -2.066775 -1.175875 -0.825208

O -1.707369 1.528806 -1.177163

O 0.697590 0.991443 -2.400928

Nb 1.311003 0.150699 2.288177

O -0.335672 -1.071431 2.643288

O 2.083050 -1.514766 1.350431

O 2.505695 1.201494 1.061202

O 0.023944 1.683393 2.323894

O 2.164213 0.124586 3.846807

Nb 1.950681 1.269905 -0.858986

Nb 1.320759 -1.995539 -0.383445

Nb -1.522129 -1.328012 1.107048

Nb -1.039941 1.985387 0.626426

O -2.228851 0.467423 1.303246

O 0.593688 2.649819 -0.213007

O 2.629849 -0.523700 -1.116493

O -0.235175 -2.791887 0.469443

O 3.256935 2.407914 -1.640992

O -1.985141 3.520856 1.107565

O -2.906410 -2.457308 1.820549

O 2.377648 -3.534849 -0.930382

Cs -4.745187 0.559366 -0.702880

Cs -2.500443 0.825761 4.212445

Cs 1.402922 -3.773714 3.097414

Cs 2.044733 4.225315 1.908129

Cs 5.015889 -0.751960 0.902131

Cs -3.165726 -3.834001 -1.481869

Cs -0.229647 3.986694 -2.793118

Cs 2.950297 -1.935740 -3.745733

O -4.772451 -0.860901 2.589199

O -5.158774 -2.353995 0.634467

O 2.542834 4.886283 -1.358185

O 4.484880 4.058168 -0.062257

O 4.847487 -3.161934 -1.252066

O 4.036452 -3.895379 0.988763

O -2.507056 -2.672579 -4.365911

O -4.230818 -1.269403 -3.284458

S -3.199584 -1.347518 -4.372754

S -4.691960 -2.267233 2.062748

S 3.982322 -4.148504 -0.499116

S 3.851213 4.143220 -1.416968

S -3.346184 4.645493 0.159122

O -2.572090 5.033319 -1.052886

O -4.395814 3.628027 -0.113747

51

Cs8PONb_6so2(Ot) -5219.86423781 -5219.673531 -5219.868657 0

Nb -1.154360 0.258501 -2.118971

O -2.213583 0.418047 -3.649559

O 0.155972 -0.020322 -0.094969

O 0.066838 -1.277950 -2.504474

O -2.199768 -0.982045 -0.996697

O -1.835076 1.751220 -0.937829

O 0.436963 1.436635 -2.447838

Nb 1.437015 -0.163043 1.935428

O -0.134189 -1.424874 2.248651

O 2.114753 -1.657104 0.730191

O 2.537302 1.056153 0.853483

O 0.204468 1.316769 2.355922

O 2.447049 -0.446638 3.481883

Nb 1.808078 1.462369 -1.012068

Nb 1.207582 -1.866802 -1.000025

Nb -1.488070 -1.442137 0.834043

Nb -1.028498 1.904790 0.849946

O -2.152822 0.294852 1.395407

O 0.560849 2.692824 -0.006975

O 2.448387 -0.273918 -1.568156

O -0.279543 -2.781803 -0.153090

O 3.095746 2.676397 -1.689315

O -1.876002 3.360950 1.623991

O -2.813154 -2.651752 1.476478

O 2.203562 -3.263804 -1.879917

Cs -4.862208 0.634944 -0.314269

Cs -2.078933 0.033837 4.408889

Cs 1.601301 -4.208617 2.175429

Cs 2.172313 3.916406 2.249891

Cs 5.045789 -0.741171 0.078901

Cs -3.367580 -3.555360 -1.904355

Cs -0.505523 4.491426 -2.210883

Cs 2.482904 -1.143522 -4.463179

O -4.638393 -1.162838 2.580809

O -5.209378 -2.512971 0.573235

O 2.419325 5.054110 -0.845390

O 4.477661 4.018383 0.065969

O 4.600782 -2.722684 -2.508419

O 4.169509 -3.829891 -0.321456

O -3.004280 -1.931846 -4.635478

O -4.569446 -0.739930 -3.142820

S -3.691939 -0.638777 -4.353267

S -4.598899 -2.520884 1.946012

S 3.898951 -3.858138 -1.800872

S 3.716685 4.366515 -1.173999

S -3.374587 4.596389 0.988262

O -2.695853 5.249540 -0.163244

O -4.391313 3.593291 0.582508

S 2.049760 -1.275691 5.240111

O 0.695753 -0.719428 5.520211

O 2.016053 -2.712899 4.846172

55

Cs8PONb-iMPA/CO2(Ot) -2842.91171327 -2842.609097 -2842.778721 0

P -6.255509 0.241708 0.503965

C -7.796073 0.636868 -0.373918

O -5.277491 1.396570 0.389803

O -6.559683 -0.372874 1.847239

O -5.560126 -0.897650 -0.507106

C -6.033483 -2.254949 -0.531829

C -5.452332 -3.057572 0.617174

C -5.609351 -2.822215 -1.872641

O -2.099079 -0.037140 0.098716

Nb -1.095129 -1.755777 -0.326913

O 0.615427 -2.789182 -0.597800

Nb 2.370115 -1.776031 -0.436909

O 3.617767 -3.020305 -0.761116

Nb -0.821639 1.439393 0.405295

O 0.601727 0.015247 0.028383

Nb 0.740859 -0.619479 2.402419

O 2.081689 -1.887187 1.572756

O -2.320564 2.894783 0.765276

O -0.777260 1.778928 -1.568209

Nb 0.579406 0.475191 -2.396334

O 1.955479 -1.014697 -2.265378

O -0.658620 0.924315 2.315109

O 0.746531 2.683219 0.605423

Nb 2.461059 1.604585 0.313829

O 3.470534 -0.109171 -0.089979

O 2.035050 1.715197 -1.661912

O 0.598460 0.827875 -4.151858

O -0.840440 -0.949474 -2.175797

O 2.134400 0.849713 2.183287

O 3.787335 2.786580 0.571182

O -2.317231 -3.040966 -0.598655

O -0.720841 -1.814283 1.679071

O 0.876902 -1.036491 4.139639

Cs 0.568579 -4.489546 1.917647

Cs 0.813869 3.229009 3.535542

Cs 4.640183 -0.707112 2.631604

Cs 0.395971 -3.272610 -3.573417

Cs 0.564095 4.443194 -1.889220

Cs -3.601623 0.737134 -2.350075

Cs 4.476972 0.508752 -2.883475

Cs -3.466720 -0.416213 2.714894

H -4.474564 2.855513 0.056294

H -7.134843 -2.245933 -0.456993

H -5.971009 -3.848627 -1.994638

H -6.007777 -2.221358 -2.698510

H -4.513342 -2.843745 -1.922782

H -5.794567 -4.097718 0.561336

H -4.357753 -3.058762 0.537222

H -5.779351 -2.635537 1.570792

H -8.350651 1.390278 0.190048

H -7.587039 1.033325 -1.371726

H -8.429412 -0.250213 -0.466371

C -2.791240 3.807752 -0.041083

O -4.085402 3.657900 -0.379182

O -2.156641 4.759258 -0.496359

55

Cs8PONb-iMPA/SO2(Ot) -3202.92023274 -3202.622910 -3202.794065 0

P 6.055726 -0.002274 -0.150781

C 7.767860 0.140070 0.438500

O 5.203481 1.031390 0.568038

O 6.042464 -0.140644 -1.653113

O 5.566097 -1.435639 0.567785

C 5.991248 -2.714573 0.080070

C 5.183024 -3.145333 -1.130062

C 5.815700 -3.683431 1.233868

O 2.112443 -0.521953 -0.085192

Nb 0.810568 -2.073408 -0.086820

O -1.082449 -2.851197 -0.062623

Nb -2.626310 -1.561999 0.017970

O -4.074613 -2.617853 0.031779

Nb 1.069806 1.198917 -0.020840

O -0.576060 -0.034282 -0.002401

Nb -0.733257 -0.054362 -2.469525

O -2.307612 -1.216423 -1.955177

O 2.916611 2.283057 -0.096682

O 0.982145 1.034091 1.969983

Nb -0.599768 -0.164412 2.462405

O -2.204516 -1.310071 1.982988

O 0.863353 1.136447 -2.006034

O -0.199254 2.694381 0.060393

Nb -2.141122 1.862167 0.079767

O -3.430722 0.326935 0.079112

O -1.767114 1.454648 2.027227

O -0.616624 -0.225088 4.254632

O 0.561767 -1.722925 1.906368

O -1.867041 1.548547 -1.901902

O -3.223891 3.290104 0.140787

O 1.801218 -3.567203 -0.135265

O 0.467697 -1.628394 -2.044776

O -0.843080 -0.047663 -4.258980

Cs -1.254082 -3.913478 -2.892862

Cs -0.091912 3.961988 -2.666468

Cs -4.601606 0.579133 -2.690776

Cs -1.114608 -4.055168 2.708371

Cs 0.036765 3.804558 2.863374

Cs 3.437602 -0.615149 2.656047

Cs -4.444376 0.454862 2.941328

Cs 3.285752 -0.418238 -2.917284

H 3.777021 1.815970 0.104887

H 7.059846 -2.656751 -0.190960

H 6.181043 -4.680669 0.969171

H 6.358786 -3.341224 2.120848

H 4.750416 -3.775715 1.478020

H 5.439094 -4.173828 -1.409383

H 4.110187 -3.118201 -0.895418

H 5.408352 -2.492479 -1.976214

H 8.206409 1.060278 0.045830

H 7.801697 0.176452 1.530269

H 8.374040 -0.699911 0.086887

O 2.473990 4.706892 -1.140289

O 2.584525 4.560727 1.331699

S 3.260425 4.247238 0.040016
